# Complement Regulates Nutrient Influx and Metabolic Reprogramming during Th1 Cell Responses

**DOI:** 10.1016/j.immuni.2015.05.024

**Published:** 2015-06-16

**Authors:** Martin Kolev, Sarah Dimeloe, Gaelle Le Friec, Alexander Navarini, Giuseppina Arbore, Giovanni A. Povoleri, Marco Fischer, Réka Belle, Jordan Loeliger, Leyla Develioglu, Glenn R. Bantug, Julie Watson, Lionel Couzi, Behdad Afzali, Paul Lavender, Christoph Hess, Claudia Kemper

**Affiliations:** 1 Division of Transplant Immunology and Mucosal Biology, MRC Centre for Transplantation, King’s College London, Guy’s Hospital, Great Maze Pond, London SE1 9RT, UK; 2 Department of Biomedicine, Immunobiology, University of Basel, 20 Hebelstrasse, 4031 Basel, Switzerland; 3 Department of Dermatology, University Hospital Zurich, 31 Gloriastrasse, 8091 Zürich, Switzerland; 4 Biomedical Research Centre, King’s Health Partners, Guy’s Hospital, Great Maze Pond, London SE1 9RT, UK; 5 MRC and Asthma UK Centre in Allergic Mechanisms of Asthma, King’s College London, Guy’s Hospital, Great Maze Pond, London SE1 9RT, UK; 6 Nephrology Transplantation, CHU Bordeaux, Hospital Pellegrin, CNRS UMR 1564, 146 rue Leo Saignat, 33076 Bordeaux, France; 7 Molecular Immunology and Inflammation Branch, National Institute of Arthritis and Musculoskeletal and Skin Diseases, National Institutes of Health, 10 Center Drive, Bethesda, MD 20892, USA

## Abstract

Expansion and acquisition of Th1 cell effector function requires metabolic reprogramming; however, the signals instructing these adaptations remain poorly defined. Here we found that in activated human T cells, autocrine stimulation of the complement receptor CD46, and specifically its intracellular domain CYT-1, was required for induction of the amino acid (AA) transporter LAT1 and enhanced expression of the glucose transporter GLUT1. Furthermore, CD46 activation simultaneously drove expression of LAMTOR5, which mediated assembly of the AA-sensing Ragulator-Rag-mTORC1 complex and increased glycolysis and oxidative phosphorylation (OXPHOS), required for cytokine production. T cells from CD46-deficient patients, characterized by defective Th1 cell induction, failed to upregulate the molecular components of this metabolic program as well as glycolysis and OXPHOS, but IFN-γ production could be reinstated by retrovirus-mediated CD46-CYT-1 expression. These data establish a critical link between the complement system and immunometabolic adaptations driving human CD4^+^ T cell effector function.

## Highlights

• CD46 regulates GLUT1 and LAT1 and enhances glucose and AA uptake in T cells
• LAMTOR5 mediates Ragulator-Rag-mTORC1 assembly in activated T cells
• Complement drives glycolysis and oxidative phosphorylation critical to Th1 cell induction

## Graphical Abstract

**Figure:**
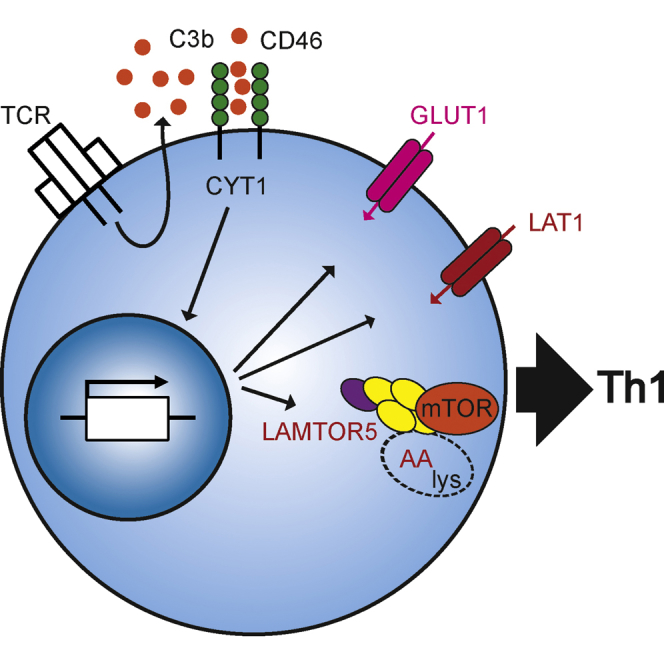

## Teaser

The in vivo signals that drive metabolic reprograming of activated T cells remain poorly understood. Kemper and colleagues demonstrate that complement C3b enhances nutrient uptake and sensing in human T cells, enabling increased glycolysis and respiration required for Th1 responses.

## Introduction

Naive T cells are metabolically quiescent, primarily depending on oxidative phosphorylation (OXPHOS) for homeostatic adenosine triphosphate (ATP) generation (Gubser et al., 2013; Pearce et al., 2013; Rathmell, 2012; van der Windt et al., 2012, 2013). Ligation of the T cell receptor (TCR) and costimulatory molecules initiates significant changes in nutrient uptake and usage of metabolic pathways, jointly supporting bioenergetic and non-bioenergetic requirements of activated T cells (Gerriets and Rathmell, 2012; Jacobs et al., 2008; Pearce et al., 2013; Wang et al., 2011). Enhanced cellular uptake of amino acids (AA) is mediated by increased expression of several system L amino-acid transporters—particularly SLC7A5 (which together with SLC3A2 forms the neutral AA transporter LAT1). *SLC7A5*-deficient T cells, or T cells with compromised SLC3A2 expression, demonstrate broad metabolic defects and fail to proliferate and acquire effector functions (Sinclair et al., 2013; Wang et al., 2011). Likewise, upregulation of the glucose transporter GLUT1 (SLC2A1) and increased glycolytic flux (aerobic glycolysis) are needed for growth, expansion, and effector functionality (Macintyre et al., 2014) as is upregulation of OXPHOS, which supports T cell proliferation, interleukin (IL)-2 production, and enhanced glycolysis (Sena et al., 2013). Thus, activation of T cells is highly dependent on increasing AA-uptake, glycolytic flux, and OXPHOS.

The metabolic-checkpoint kinase mechanistic target of rapamycin (mTOR) senses and integrates environmental signals to regulate metabolic activity in cells. Activation of mTOR triggers glycolysis, OXPHOS, and lipid synthesis (Cunningham et al., 2007; Düvel et al., 2010; Shi et al., 2011) and mTOR-deficient CD4^+^ T cells are unable to adjust metabolically and differentiate into effector cells (Delgoffe et al., 2009; Zheng et al., 2007). mTOR signals via two distinct complexes, mTOR complex 1 (mTORC1) and mTOR complex 2 (mTORC2), with mTORC1 activity inducing the enzymes needed for glycolysis and being specifically required for normal Th1 and Th17 cell induction (Pollizzi and Powell, 2014). Cytokines, availability of oxygen, and cellular energy levels all impact mTORC1 activity via the tuberous sclerosis 1 (TSC1)-TSC2 axis (Bar-Peled and Sabatini, 2014). By contrast, sensing sufficiency of AA by mTORC1 occurs via the RAS-related GTP-binding protein (Rag) family of small GTPases (Bar-Peled et al., 2012; Groenewoud and Zwartkruis, 2013; Long et al., 2005; Sancak et al., 2010). Specifically, in the presence of AA within the lysosomal lumen, the Rag A-B heterodimer becomes GTP-loaded and induces mTORC1 translocation to lysosomes, bringing it into close proximity of its activator Rheb (Groenewoud and Zwartkruis, 2013; Long et al., 2005; Sancak et al., 2010). The Rags are bound to lysosomes via the pentameric Ragulator complex, which consists of the “late endosomal or lysosomal adaptor and MAPK and mTOR activator” (LAMTOR 1–5) proteins (Sancak et al., 2010), and this LAMTOR-Rag-Rheb-mTORC1 complex then permits metabolic activation.

While the importance of metabolism in enabling T cell activation and differentiation is established, the in vivo pathways directing those events remain poorly defined. This is particularly true with regard to our understanding of metabolic reprogramming in human T cells. CD46, initially discovered as a complement regulator that binds and inactivates C3b and C4b (Liszewski et al., 1991), is also a key costimulatory molecule on human CD4^+^ T cells (Astier et al., 2000; Cardone et al., 2010; Kemper et al., 2003; Le Friec et al., 2012). Importantly, CD46 is not expressed on hematopoietic cells in rodents and has no known functional homolog (Cope et al., 2011). In humans, CD46 is ubiquitously expressed in four distinct isoforms, with one of two alternatively spliced cytoplasmic tails, termed CYT-1 and CYT-2 (Liszewski and Atkinson, 1996), each mediating distinct signaling events (Cope et al., 2011). CD46-transduced signals are critically required for the induction of IFN-γ in human CD4^+^ T cells, with indications that CYT-1 is driving Th1 cell polarization (Le Friec et al., 2012; Ni Choileain et al., 2011). Together with IL-2, CD46 also mediates IL-10 coexpression in expanded Th1 cells, and via this the switch toward a (self)regulatory contraction phase (Cardone et al., 2010). Unexpectedly, CD46-mediated activation of T cells is independent of systemic C3, but driven in an autocrine manner by the C3 activation fragment C3b generated by the T cell itself upon TCR activation (Cardone et al., 2010; Liszewski et al., 2013). Thus, lack of autocrine CD46 activation, such as in CD46- and C3-deficient patients, results in subnormal Th1 cell responses (Le Friec et al., 2012), whereas uncontrolled autocrine C3 activation and dysregulated CD46 engagement contribute to hyperactive Th1 cell responses in autoimmune pathologies (Astier et al., 2006; Cardone et al., 2010; Liszewski et al., 2013).

In this report we postulate a link between complement and key metabolic events regulating the human Th1 cell response. We characterize the dominant role of CD46 over CD28 in regulating GLUT1, LAT1, and nutrient uptake, define LAMTOR5 as part of the AA sensing machinery in human CD4^+^ T cells, and develop a model interlinking metabolic reprogramming with CD46-mediated Th1 cell activation and contraction.

## Results

### Autocrine CD46 Activation Is Required for Normal Glycolysis and OXPHOS

**Figure 1:**
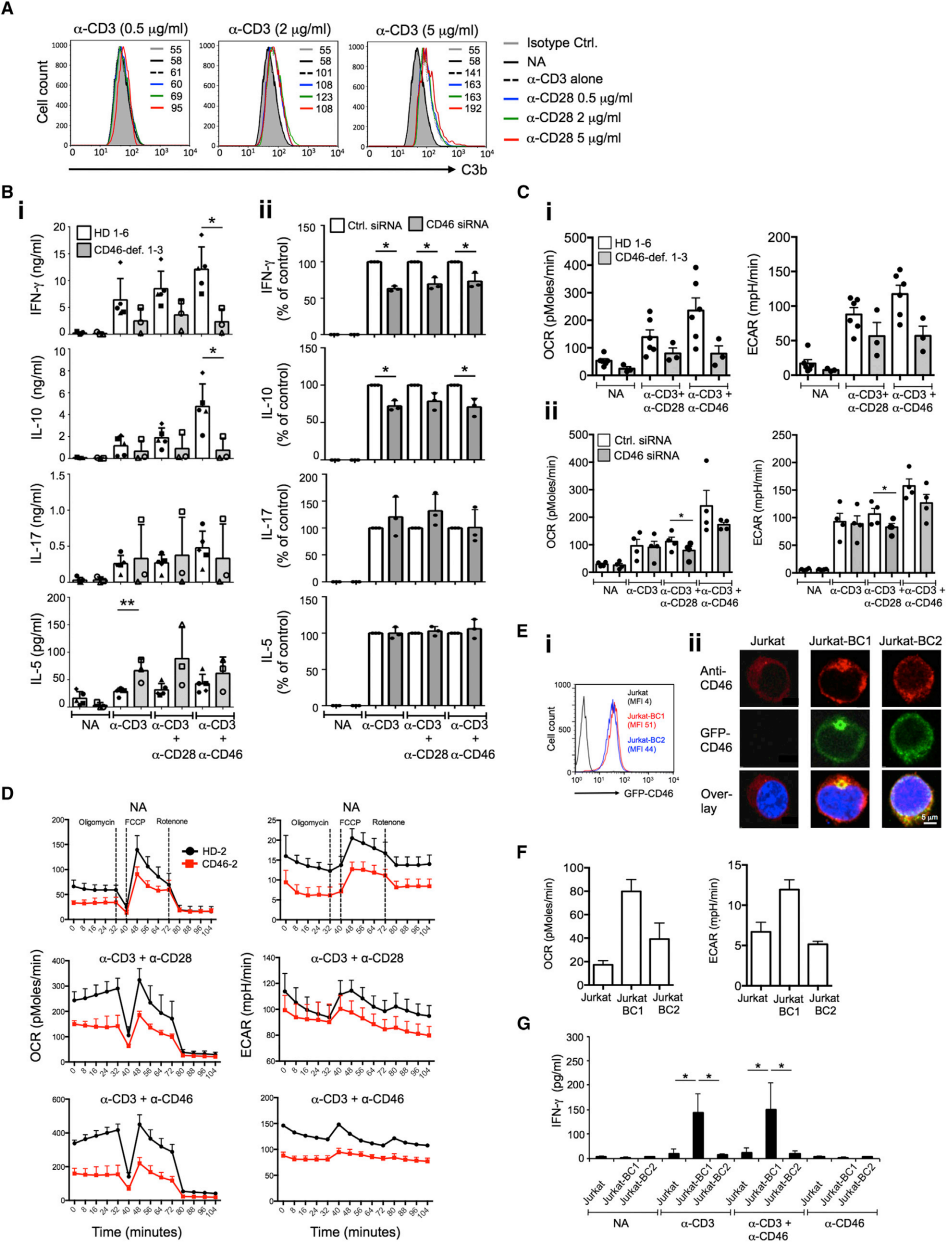
Autocrine CD46-CYT-1 Activation Drives Glycolysis and Oxidative Phosphorylation in CD4^+^ T Cells (A) TCR and CD28-induced Th1 cell cytokine production correlates with CD46 ligand C3b generation as assessed 1 hr post activation. (B) Cytokines produced by (Bi) CD4^+^ T cells from age- and sex-matched healthy donors (HD1 to HD6) and patients CD46-1 (open circle), CD46-2 (open square), and CD46-3 (open triangle) or by (Bii) T cells from HDs treated with CD46 siRNA (n = 3 with duplicate samples [mean]). (C) Basal glycolysis (ECAR) and oxidative phosphorylation (OXPHOS, OCR) rates in resting and activated CD4^+^ T cells (Ci) from CD46-deficient patients (n = 3) and HDs (n = 6) or from (Cii) HD T cells after CD46-specific siRNA treatment. (D) Respiratory capacity and glycolysis in T cells from a HD and from patient CD46-2, basally and following mitochondrial perturbation. (E) CD46 expression in Jurkat T cells transfected with GFP-tagged CD46-CYT1 (Jurkat-BC1) or CD46-CYT2 (Jurkat-BC2) isoforms. (Ei) FACS-assessed surface expression of GFP-tagged CD46 and (Eii) endogenous (red) and recombinantly overexpressed CD46 (green) by confocal microscopy (n = 3). (F) Basal glycolysis and OXPHOS levels in Jurkat, Jurkat-BC1, and Jurkat-BC2 cells (n = 3). (G) CD46-BC1 isoform overexpression restores IFN-γ upon TCR activation in Jurkat cells (n = 3, IFN-γ measured 3 days post activation). Magnification (Eii) × 100. ^∗^p < 0.05; ^∗∗^p < 0.01. Error bars represent mean ± SEM. See also Figure S1.

Autocrine CD46 activation by TCR-driven generation of C3b is an integral and non-redundant part of human Th1 cell induction and contraction (Liszewski et al., 2013). Activation of T cells with increasing amounts of monoclonal anti-CD3 and/or anti-CD28 mAbs was associated with larger surface deposition of C3b (Figure 1A), as well as IFN-γ production and IL-10 switching (Figure S1A). This suggested that modulation of Th1 cell cytokine production by varying TCR and costimulatory signal strength (Viola and Lanzavecchia, 1996) is impacted by CD46-mediated signaling. IFN-γ production by murine CD4^+^ T cells is accompanied by specific metabolic changes (Chang et al., 2013), which led us to interrogate whether CD46 regulates human Th1 cell responses via modulation of key metabolic pathways. We used T cells from three patients with absent or severely reduced CD46 expression (CD46-1, CD46-2, and CD46-3 [Couzi et al., 2008; Fremeaux-Bacchi et al., 2006; and Figure S1B, legend]), T cells from healthy donors (HDs) in which CD46 protein expression was reduced by siRNA technique, and Jurkat T cell lines overexpressing specific CD46 isoforms. Whole-exome sequencing of DNA samples from patients CD46-2 (sibling of CD46-1) and CD46-3 confirmed the expected mutations in *CD46* but did not identify additional mutations in candidate genes mediating T cell function or genes known to cause monogenic immune defects (Table S1 and S2). While expression of CD3 and CD28 on T cells from all three patients was within normal range (Figure S1B), their CD4^+^ T cells demonstrated impaired acquisition of Th1 cell effector function in response to TCR ligation and costimulation via either CD46 or CD28 (Cardone et al., 2010; Le Friec et al., 2012) (Figure 1Bi). The phenotype of T cells from HDs treated with CD46-specific siRNA (Figure S1Ci) was comparable, with a specific reduction in IFN-γ and IL-10, but normal IL-5 production (Figure 1Bii), and reduced upregulation of CD25 (Ni Choileain et al., 2011), but unaltered expression of CD69 (Figure S1Cii) (Le Friec et al., 2012). Only T cells from patient CD46-3, which lacked CD46 expression entirely (Figure S1B, legend), were unable to produce IL-17.

As upregulation of mitochondrial respiration (OXPHOS) and aerobic glycolysis is central to T cell effector function, we assessed the metabolic profile (mitochondrial respiration (oxygen consumption rate – OCR)), and aerobic glycolysis (extracellular acidification rate – ECAR) of T cells from the CD46-deficient patients and six HDs (Figure 1Ci). Compared to controls, non-activated and CD3+CD28-activated CD4^+^ T cells from patients showed a trend toward reduced respiration and glycolysis (Figure 1Ci), and the increase in OCR and ECAR levels upon CD3+CD46 activation—which was present in all HDs—was absent in CD46-deficient individuals (Figure 1Ci). Notably, by 36 hr of activation, similar OCR and ECAR levels were observed in CD3 and CD3+CD28-activated HD CD4^+^ T cells (Figures S1Ei and S1Eii). A comparable reduction in OCR and glycolysis was observed using HD-derived T cells in which CD46 expression was knocked down (Figure 1Cii), with the reduction of respective CD46 expression corresponding with reduction in OCR and ECAR (not shown). We also observed a parallel reduction in ATP-coupled and maximal respiration, as well as maximal glycolytic rate of activated cells, when comparing CD46-deficient CD4^+^ T cells to those from HDs (Figure 1D, patient CD46-2; Figure S1F, summary for all three patients; and Figure S1G for HD T cells after CD46-specific siRNA treatment).

CD46 is expressed in distinct isoforms with two potential intracellular domains, CYT-1 or CYT-2 (Figure S1H), and CYT-1 is required for IFN-γ production in CD4^+^ T cells (Le Friec et al., 2012). To assess whether CYT-1 also mediated the metabolic changes observed during T cell activation, we used non-manipulated Jurkat T cells (which mostly express CD46 CYT-2-bearing isoforms and do not produce IFN-γ upon activation), or Jurkat cells stably overexpressing either a GFP-tagged CD46 isoform bearing CYT-1 (Jurkat-BC1) or CYT-2 (Jurkat-BC2) (Le Friec et al., 2012) at equivalent levels (Figure 1Ei), and with normal cellular distribution (Figure 1Eii). Jurkat-BC1 cells indeed had increased basal OCR and ECAR compared to non-transfected or Jurkat-BC2 cells (Figure 1F) and, importantly, this increase was accompanied by the induction of IFN-γ production upon activation (Figure 1G).

Thus, autocrine C3b-driven activation of CD46 induced the metabolic changes in human CD4^+^ T cells that drive Th1 cell induction.

### CD46 Costimulation Is Non-redundant and Operates Differently from CD28

**Figure 2:**
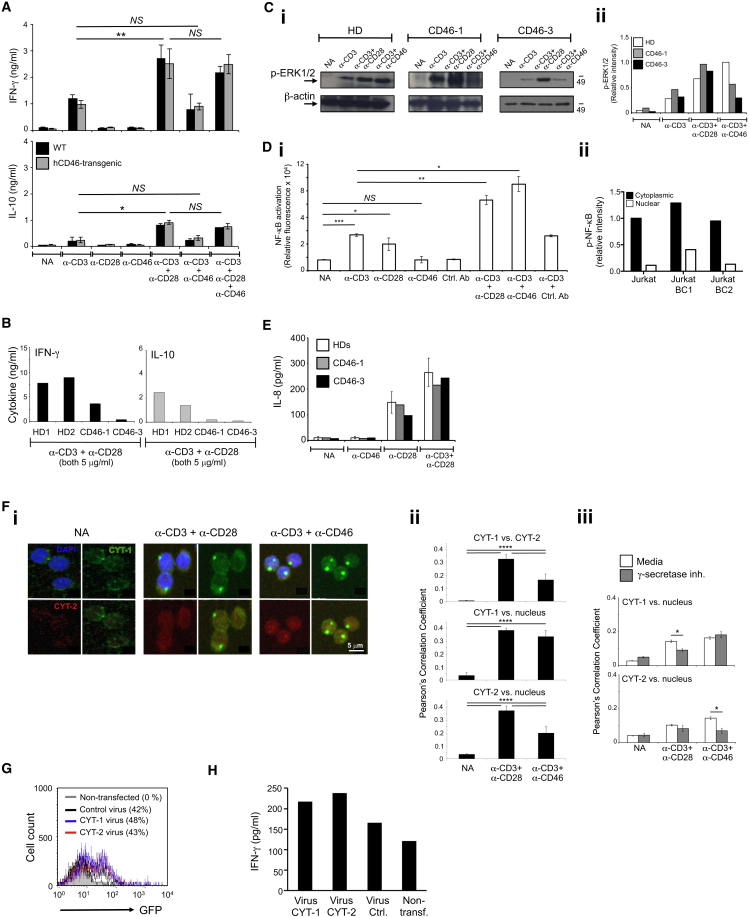
CD46 Costimulation Is Human Specific and Operates Differently from CD28 (A) CD4^+^ T cells from hCD46-transgenic or wild-type (WT) mice were stimulated with anti-mouse CD3 and CD28 and anti-human CD46 and cytokines measured 72 hr post activation (n = 3). (B) Increased TCR and CD28 activation cannot rescue defective Th1 cell induction in CD46-deficient T cells. Cells from HD1 and HD2 and patients CD46-1 and CD46-3 were activated as indicated and cytokines measured at 36 hr. (C) TCR and CD28-driven ERK1/2 phosphorylation occurs optimally in CD46-deficient T cells as assessed by (Ci) western blot and (Cii) densitometric analyses 30 min post activation. (D) CD46 induced canonical NF-κB activation utilizing (Di) T cells transfected with a NF-kB luciferase reporter plasmid and NF-κB activation measured at 1 hr post activation and measuring (Dii) NF-κB activation in Jurkat, Jurkat-BC1, and Jurkat-BC2 cells (n = 4). (E) CD28 induces normal IL-8 secretion in T cells from patients at 36 hr post activation. (F) CD46 CYT-1 and CYT-2 translocate to the nucleus upon cleavage by γ-secretase as assessed by (Fi) confocal microscopy using CYT-1 and CYT-2-specific antibodies with analyses of colocalization events in (Fii) the absence or (Fiii) presence of γ-secretase inhibition (n = 3). (G and H) Transfection of CD46 intracellular domains rescues IFN-γ production in T cells from patient CD46-3. (G and H) Transfection efficiency (G) of T cells isolated from patient CD46-3 transfected with retroviruses expressing either CYT-1 or CYT-2 (or the GFP reporter gene) and (H) IFN-γ production by CD4^+^ T cells from patient CD46-3 after retroviral transfection at 24 hr post CD3+CD28 activation. ^∗^p < 0.05; ^∗∗^p < 0.01; ^∗∗∗^; p < 0.005; ^∗∗∗∗^p < 0.001; NS, statistically not significant. Error bars represent mean ± SEM. See also Figure S2.

Mice lack CD46 expression on lymphocytes (Cope et al., 2011), and CD4^+^ T cells isolated from transgenic mice expressing human CD46 in a human-like pattern (Kemper et al., 2001) did not increase IFN-γ or IL-10 production upon activation (Figure 2A). Thus, mouse T cells are not equipped with the machinery for CD46-induced signal transduction, and CD28 is the critical costimulatory molecule in this species.

Defective Th1 cell induction in CD46-deficient patients could not be overcome by increasing TCR and CD28 signal strength (Figure 2B), while TCR- and CD28-driven phosphorylation of extracellular signal-regulated kinase 1/2 (ERK1/2) was unaffected (Figures 2Ci and 2Cii). Furthermore, although CD46 stimulation, and specifically CYT-1, potentiated TCR-induced NF-κB activation (Figures 2Di and Dii), it did not induce NK-κB activation on its own (Figure 2Di) and failed to induce IL-8 secretion (Figure 2E)—two events that are driven by TCR-independent CD28 signals (Marinari et al., 2004) and that similarly occurred in T cells from HDs and CD46-deficient patients (Figure 2E). These data demonstrated that TCR- and CD28-mediated signals did function properly in CD46-deficient patients but that these signals were not sufficient for normal Th1 cell induction.

Upon CD46 activation, CYT-1 and CYT-2 are cleaved and released intracellularly by γ-secretase (Ni Choileain et al., 2011), and inhibition of γ-secretase activity prevents CD46-driven Th1 cell induction (Figure S2A) (Le Friec et al., 2012). Because both CYT-1 and CYT-2 of CD46 contain nuclear targeting signals (Figure 1H) we assessed whether the tails translocate into the nucleus upon activation. Using confocal microscopy (Figures 2Fi and 2Fii) and Image Stream (Figure S2B), we indeed observed nuclear translocation of both CYT-1 and CYT-2 in activated T cells. CYT-1 translocation was significantly inhibited by γ-secretase inhibitor treatment in CD3+CD28-activated T cells, while this treatment prevented CYT-2 nuclear translocation in CD3+CD46-activated cells (Figure 2Fiii), suggesting that the coordinated CD46 cytoplasmic domain processing and/or nuclear translocation may be impacted by both CD28 and CD46 stimulation. To mimic CD46 cytoplasmic domain release in CD46-deficient T cells, we transfected T cells from patient CD46-3 with retroviruses expressing either CYT-1 or CYT-2 only (Figure 2G), which induced substantially increased IFN-γ production (but not IL-4 or IL-5 production, data not shown) in these cells (Figure 2H). The unexpected observation that CYT-2 transfection also rescued IFN-γ production in CD46-deficient T cells was likely due to the fact that cleaved CYT-2 positively regulated CYT-1 expression, as demonstrated by assessment of both CYT-1 and CYT-2 protein expression of parental Jurkat T cells after transfection with each virus alone (Figure S2C).

Together these data demonstrated that CD46 costimulation in human CD4^+^ T cells was non-redundant and required nuclear translocation of its cytoplasmic tails to the nucleus.

### CD46 Induces Nutrient Influx via Glucose and Amino Acid Transporter Upregulation

**Figure 3:**
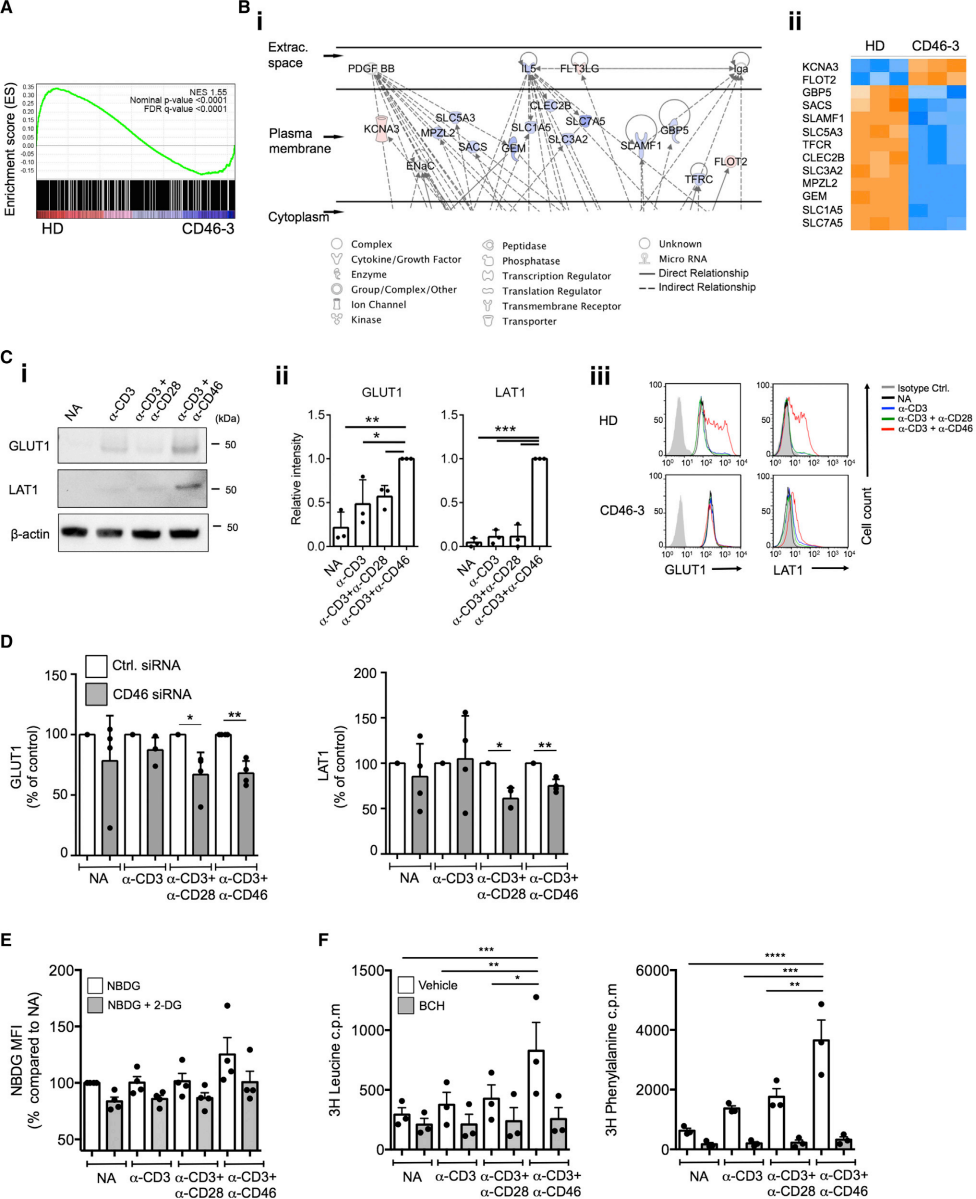
CD46 Mediates Glucose and AA Channel Expression and Nutrient Influx in CD4^+^ T Cells (A and B) Gene expression array and Ingenuity Pathway Analysis (IAP) comparison of CD46-sufficient and -deficient CD4^+^ T cells activated for 2 hr with anti-CD3+CD46 mAb with (A) gene set enrichment analysis (GSEA) and (B) extract of IPA output showing (Bi) membrane-associated genes involved in AA metabolism (full figure in Figure S2B) and (Bii) a heatmap of those genes. (C) GLUT1 and LAT1 expression on T cells from HDs 36 hr post activation assessed by (Ci) western blotting with (Cii) the corresponding statistical analyses via densitometric measurement, and (Ciii) from patient CD46-3 measured by FACS (n = 3). (D) CD46 silencing prevents normal GLUT1 and LAT1 expression (n = 4; 72 hr post activation). (E and F) Glucose and AA uptake upon CD46 activation with (E) glucose uptake assessed with or without addition of competing unlabeled 2-DG and (F) AA uptake measured with or without addition of a LAT1 inhibitor (BCH) (n = 3). ^∗^p < 0.05; ^∗∗^p < 0.01; ^∗∗∗^p < 0.005; ^∗∗∗∗^p < 0.001. Error bars represent mean ± SEM. See also Figure S3.

To identify the CD46-induced molecular pathways driving the observed metabolic events, we performed gene expression arrays on mRNA isolated from CD3+CD46-activated T cells from patient CD46-3. Aligning with the functional T cell phenotype (severely reduced glycolysis and OXPHOS, Figure 1Ci), genes associated with regulation of metabolic processes were significantly enriched in the HD compared to the patient by gene set enrichment analysis (GSEA) (Subramanian et al., 2005) (Figure 3A). Similarly, Gene Ontology analysis of the 403 transcripts differentially expressed between CD3+CD46-activated T cells of the HD and patient CD46-3 (Table S3) revealed that almost 30% of these genes (118 genes, Table S4) were functionally involved in the regulation of metabolic processes (Figures S3Ai and S3Aii). Furthermore, Ingenuity Pathway Analysis suggested that T cells from patient CD46-3 lacked activation-induced expression of several key AA transporters. Specifically, of the top five gene networks modeled by the software, two were functionally involved in AA metabolism (Figures 3Bi and 3Bii and Figures S3Bi and S3Bii). Upregulation of the glucose transporter GLUT1 (Macintyre et al., 2014) and L-type AA transporter LAT1 (SLC7A5; reduced in T cells from patient CD46-3 [Figure 3Bii]) are required for successful CD4^+^ T cell activation in mice (Sinclair et al., 2013) and humans (Hayashi et al., 2013). When assessing activated T cells 36 hr post stimulation for the expression of GLUT1 and LAT1, we found that CD3+CD46 activation induced strongest expression of these channels when compared to CD3 or CD3+CD28-activated cells (Figures 3Ci and 3Cii), while CD46 activation alone had no effect on GLUT1 or LAT1 expression (data not shown). Accordingly, T cells isolated from patient CD46-3, and T cells from HDs in which CD46 expression was decreased by siRNA treatment, had impaired GLUT1 and LAT1 upregulation (Figures 3Ciii and 3D). Whereas CD46 potentiated TCR-driven GLUT1 expression, LAT1 upregulation seemed to require CD46-driven signals, as only CD46 co-engagement induced significant LAT1 expression in T cells (Figure 3Cii). This aligns with the observation that CD3+CD46 activation moderately increased glucose uptake over CD3 and CD3+CD28-activated T cells (Figure 3E), but that LAT1-dependent uptake of the AAs Leucine and Phenylalanine at 36 hr post activation was significantly enhanced by CD46 costimulation (Figure 3F, and Figure S3C for a time course of GLUT1 and LAT1 expression and glucose and AA uptake). Consistent with the role of CD46 CYT-1 in driving glycolysis and OXPHOS, CYT-1, but not CYT-2, induced GLUT1 and LAT1 upregulation as demonstrated by the increased steady-state expression specifically in Jurkat-BC1 cells (Figures S3Di–S3Diii), and their concurrently increased basal uptake of glucose, Leucine, and Phenylalanine (Figures S3E and S3F).

These data demonstrated that CD46 CYT-1 costimulation during TCR activation was required for GLUT1 and LAT1 expression, upregulation, and subsequent glucose and AA uptake in human CD4^+^ T cells.

### CD46 Is Required for mTORC1 Activity

**Figure 4:**
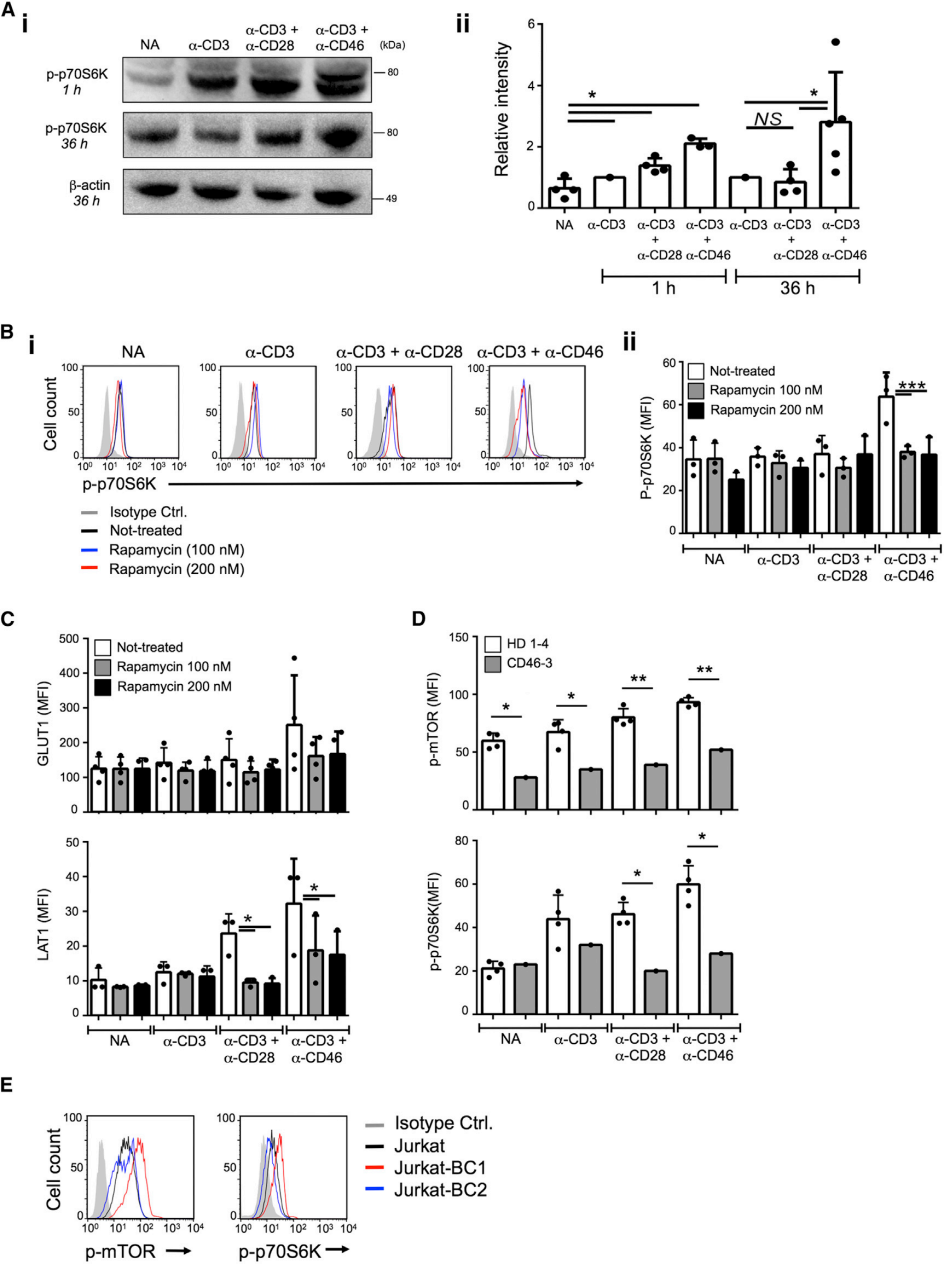
CD46 Regulates mTORC1 Activity in CD4^+^ T Cells (A) CD46 activation sustains p70S6K phosphorylation as assessed by (Ai) western blotting with (Aii) the corresponding statistical analyses via densitometric measurement of band intensities (n = 4–5). (B) Effect of rapamycin on p70S6K phosphorylation (p-p70S6K) at 36 hr with (Bi) a representative FACS analysis of n = 3, and (Bii) depicting their statistical analysis. (C) Effect of Rapamycin on GLUT1 and LAT1 expression. T cells were activated as under (B) and expression of GLUT1 (upper panel) and LAT1 (lower panel) measured (n = 4). (D) mTOR (p-mTOR) and p70S6K phosphorylation in T cells from HD1-4 and patient CD46-3 36 hr post activation. (E) p-mTOR and p-p70S6K levels in Jurkat, Jurkat-BC1, and Jurkat-BC2 cells (n = 3). ^∗^p < 0.05, ^∗∗^p < 0.01; ^∗∗∗^p < 0.005. Error bars represent mean ± SEM. See also Figure S4.

Since glucose and AA uptake induces mTORC1 activation (Bachar et al., 2009; Sancak et al., 2010), we assessed whether CD46 impacts mTORC1 activity by measuring the phosphorylation of the mTORC1 downstream target p70S6K (position T389) (Matheny and Adamo, 2009). At 1 hr post activation, CD3, CD3+CD28, and CD3+CD46 activation each induced significant increases in p70S6K phosphorylation when compared to resting cells. Notably, CD46 costimulation not only resulted in the highest levels of p-p70S6K at 1 hr post activation, but also sustained p70S6K phosphorylation consistently up to at least 36 hr post activation (Figures 4Ai and 4Aii). The observed CD46-mediated p70S6K phosphorylation was dependent on mTORC1, as the mTORC1 inhibitor Rapamycin abrogated CD46-mediated p70S6K phosphorylation (Figures 4Bi and 4Bii). Furthermore, mTORC1 activation supports upregulation of GLUT1 (Bhaskar et al., 2009) and LAT1 (Roos et al., 2009), and in accordance with these data, the addition of Rapamycin during CD3+CD46 stimulation reduced the expression of these nutrient transporters (Figure 4C). The dependence of CD4^+^ T cells on CD46 costimulation for normal mTORC1 function was further underscored by the inability of T cells from patient CD46-3 to induce either mTOR or p70S6K phosphorylation at substantial levels under any activation condition tested (Figures 4D and S4), and by a significant reduction in mTOR and p70S6K phosphorylation in T cells from HDs treated with CD46-specific siRNA (not shown). In keeping with the fact that CD46 CYT-1 was the driver of nutrient influx, glycolysis, and OXPHOS in T cells, Jurkat-BC1 cells had higher mTOR and p70S6K phosphorylation levels compared to Jukat-BC2 cells or the parental Jurkat line (Figure 4E).

### CD46 Activation Supports LAMTOR5-Driven Assembly of Ragulator-Rag-mTORC1

**Figure 5:**
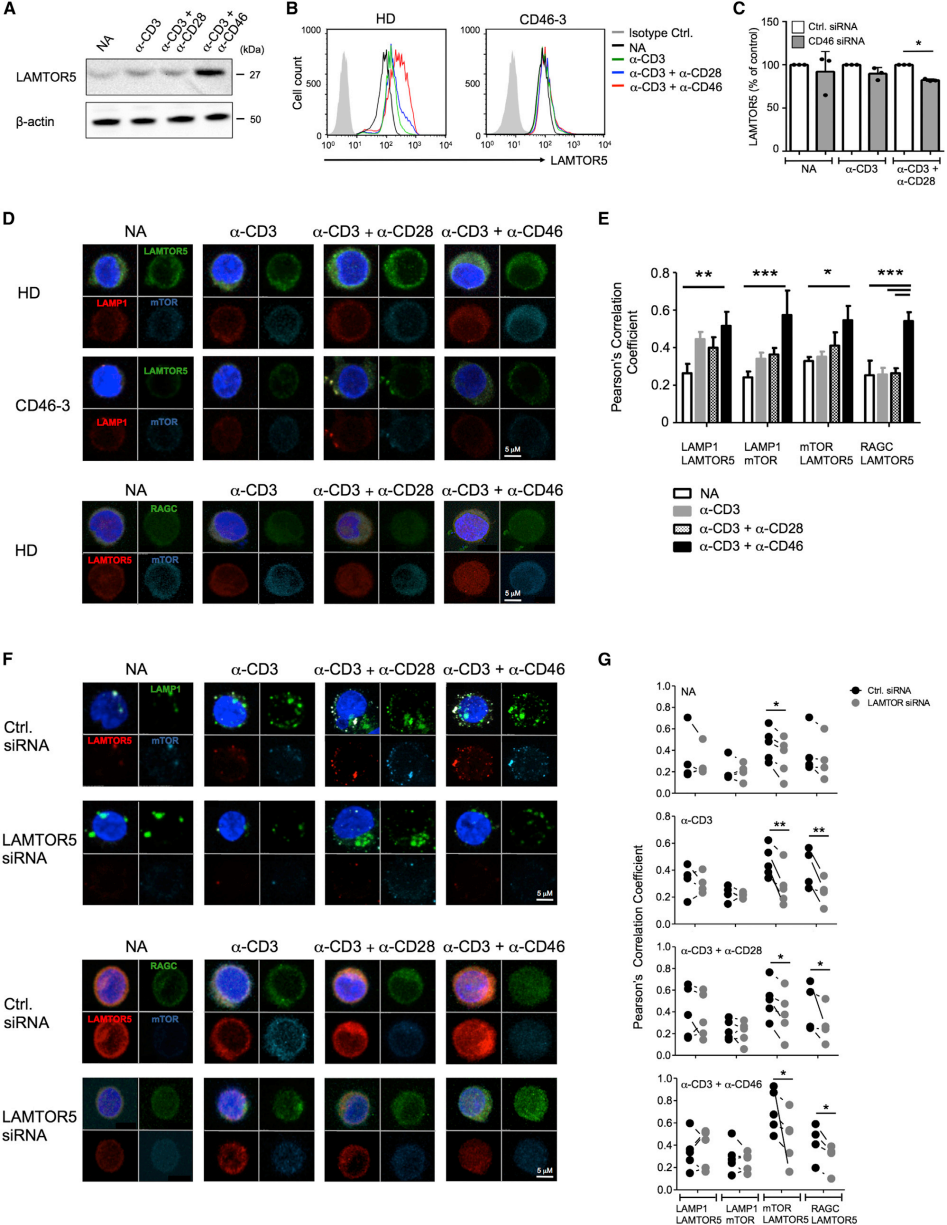
LAMTOR5 Is Required for mTORC1 Complex Assembly in Human CD4^+^ T Cells (A–C) LAMTOR5 expression in T cells from (A) a healthy donor (HD) assessed by western blotting, (B) in patient CD46-3 and a HD by FACS analysis, and in (C) HD T cells treated with CD46-specific siRNA at 72 hr post activation. (D) CD46 activation increases LAMTOR5-dependent assembly of the lysosome-based machinery enabling amino acid sensing via mTORC1 as assessed at 36 hr post activation by confocal microscopy. For the HDs, one representative example is shown for n = 7. Staining of RAGC could not be performed on cells from patient CD46-3. (E) Statistical analysis for the colocalization events in HD T cells of the proteins assessed under (D) (n = 7). (F) Reduction of LAMTOR5 expression prevents normal mTORC1 assembly measured at 36 hr post activation by (F) confocal microscopy, and (G) colocalization of proteins measured with the Pearson’s Correlation Coefficient method. Results shown in (F) and (G) are representative n = 5. Magnification (C and E) × 100. ^∗^p < 0.05; ^∗∗^p < 0.01; ^∗∗∗^p < 0.005. Error bars represent mean ± SEM. See also Figure S5.

The nature of the Ragulator complex activating mTORC1 in human CD4^+^ T cells is undefined. LAMTOR5 is a recently discovered member of the Ragulator complex (Bar-Peled et al., 2012) and, although LAMTOR5 has previously not been described in T cells, the corresponding mRNA was induced in gene arrays performed using non-activated and CD3+CD46-activated CD4^+^ T cells from HDs (data not shown). Indeed, measurement of LAMTOR5 protein in purified healthy CD4^+^ T cells established that CD46 costimulation induced a significant increase in LAMTOR5 (Figures 5A and S5A for a time-course), while this increase was absent in T cells from patient CD46-3 (Figure 5B), and in T cells from HDs where CD46 expression was decreased by siRNA technique (Figure 5C). Confocal microscopy studies with analyses of protein colocalization coefficients demonstrated that TCR activation, specifically with CD46 costimulation, drove colocalization of LAMTOR5, the Ragulator complex partner GTPase RagC, and mTOR on lysosomes (LAMP1) (Figures 5D and 5E), with reduction of these events in T cells from patient CD46-3 (Figure 5D). Results obtained from CD46 isoform-transfected Jurkat cells confirmed that LAMTOR5 expression was increased by CYT-1 (Figures S5Bi and S5Bii), while the expression of RagC remained largely unaffected by CD46 signaling (Figure S5C).

The central role for LAMTOR5 in mTORC1 lysosomal translocation was further confirmed by the finding that knockdown of LAMTOR5 protein in T cells from HDs reduced colocalization for all assessed proteins of this complex (Figures 5F and 5G) and led to a significant decrease in LAT1 expression (by about 50%, Figure S5D) and p70S6K phosphorylation by about 75% in CD3+CD46-activated T cells (Figure S5E).

These results identified Ragulator LAMTOR5 as a critical mTORC1 assembly platform in human CD4^+^ T cells.

### CD46 Isoform Expression Correlates with Metabolic Changes in the Th1 Cell Life Cycle

**Figure 6:**
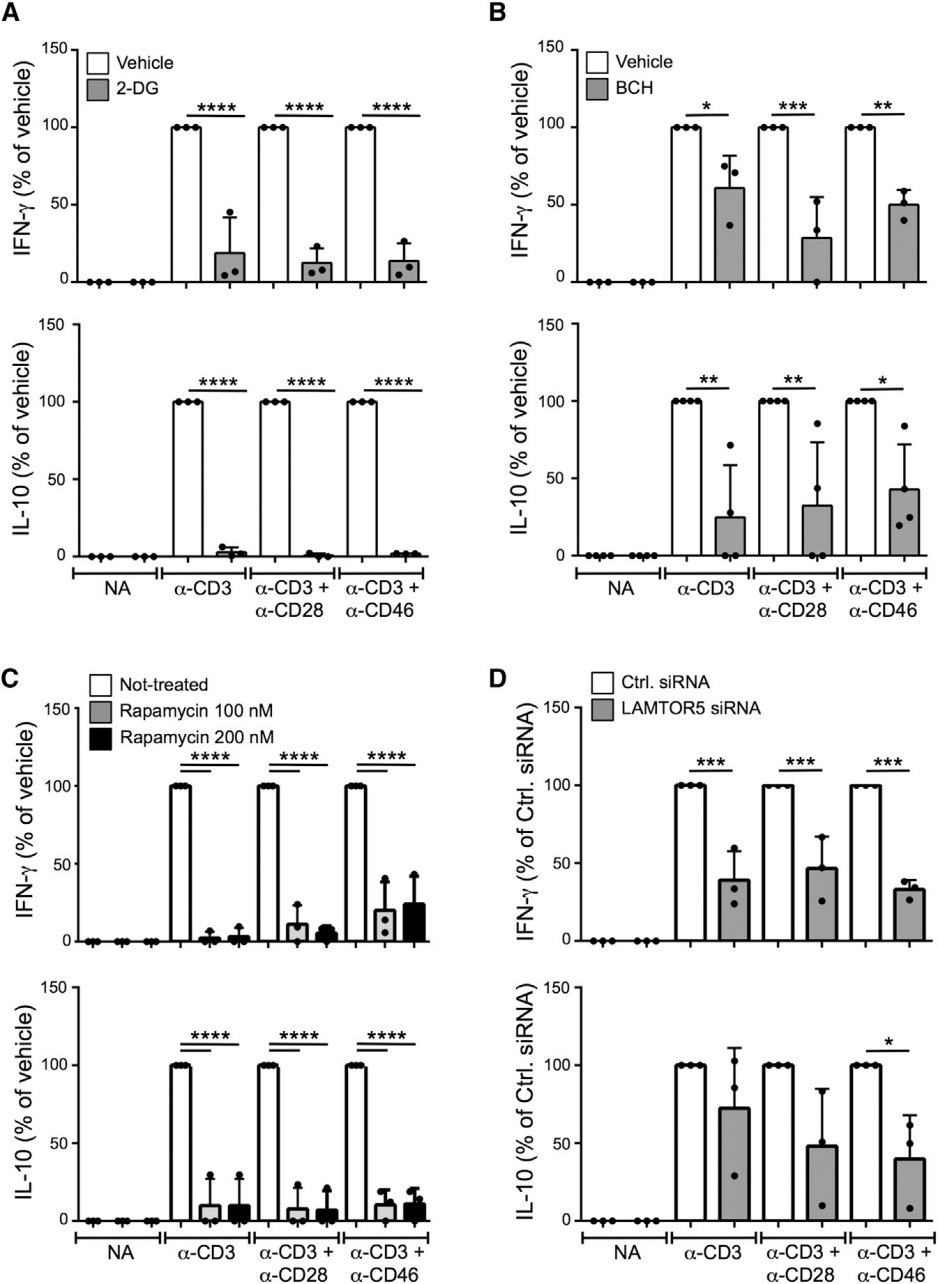
CD46-Driven Glucose and Amino Acid Influx and mTORC1-Activity Are Critical to Human Th1 Cell Induction (A and B) Th1 cell induction (upper panels) and IL-10 switching (lower panels) assessed at 36 hr post activation in the presence of 2-deoxyglucose (2-DG) and BCH. (C) Effect of mTORC1 inhibition on Th1 cell induction at 36 hr post activation. (D) Impact of LAMTOR5-silencing on Th1 cell induction. CD4^+^ T cells transfected with siRNAs as shown were activated as depicted for 36 hr and analyzed for IFN-γ and IL-10 production. Data shown in (A)–(D) are n = 3. NA, non-activated. ^∗^p < 0.05; ^∗∗^p < 0.01; ^∗∗∗^p < 0.005; ^∗∗∗∗^p < 0.001. Error bars represent mean ± SEM.

CD46 costimulation is not only key for IFN-γ production in human Th1 cells but also for the induction of IL-10 coproduction and the switch toward their (self)regulating and contracting “life cycle” phase (Cardone et al., 2010). Thus, following T cell activation via CD3+CD46 four populations of cells are sequentially induced with distinct cytokine profiles: IFN-γ^+^, IFN-γ^+^IL-10^+^, IL-10^+^, and IFN-γ^–^IL-10^–^. Accordingly, T cells from CD46-deficient patients have impaired production of both IFN-γ and IL-10 but have no defect in Th2 responses (IL-4 and IL-5) (Figure 1B and Le Friec et al., 2012). In line with the requirement of glucose and AA uptake for early IFN-γ production (Pearce et al., 2013; Sinclair et al., 2013), when we activated human CD4^+^ T cells from healthy donors via CD3, CD3+CD28, or CD3+CD46, we found that the presence of 2-DG (which inhibits glycolysis) or the LAT1 inhibitor BCH reduced production of IFN-γ and IL-10 under all activation conditions (Figures 6A and 6B, upper and lower panels, respectively). Also, inhibition of mTORC1 with Rapamycin and LAMTOR5 protein knockdown through mRNA silencing reduced both IFN-γ and IL-10 production (Figures 6C and 6D)—but did not significantly affect IL-4 and IL-17 secretion (data not shown).

**Figure 7:**
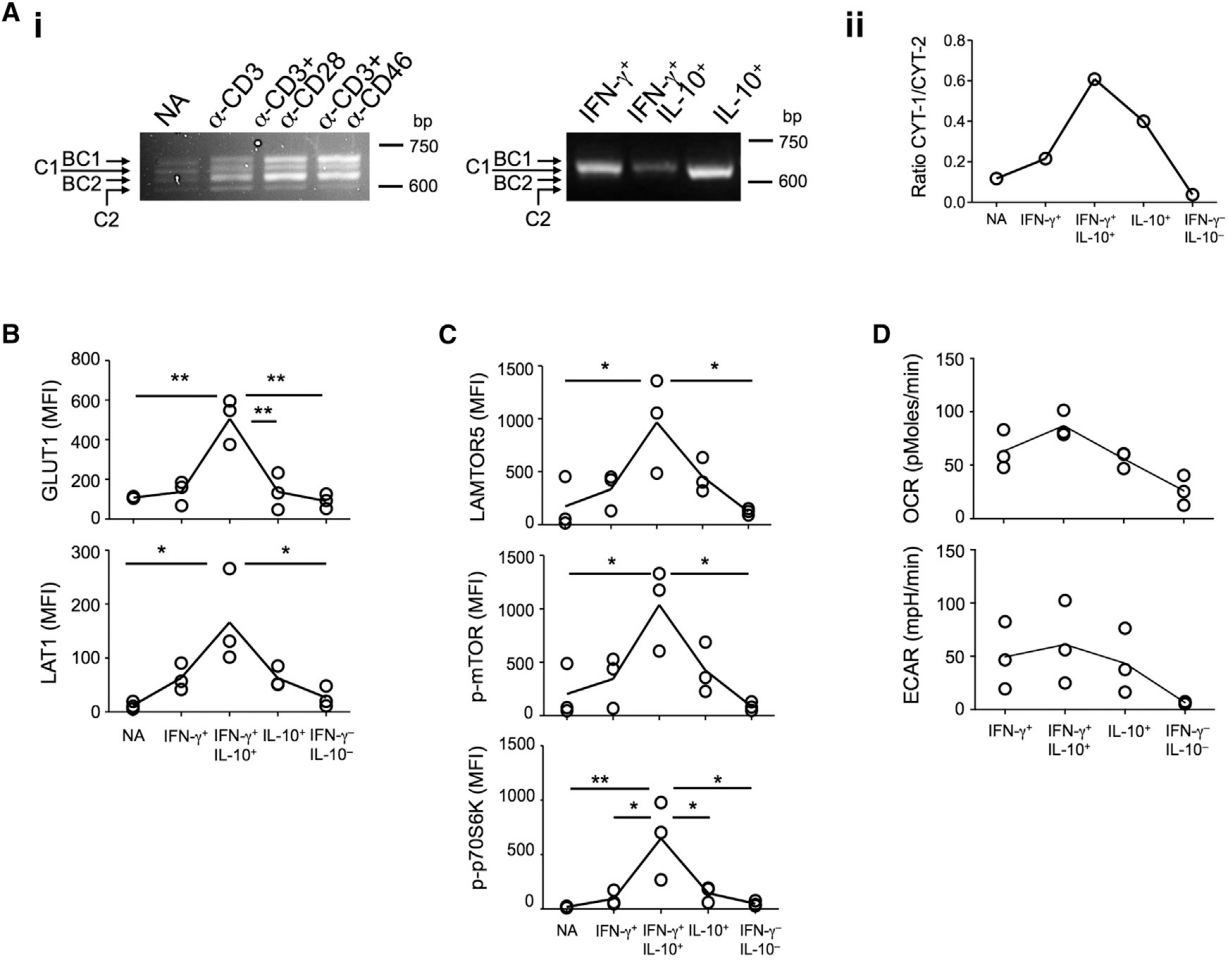
Switches in CD46 Isoform-Expression Correlate with Expected Metabolic Changes during the Th1 Cell Life Cycle (A) CD46 isoform mRNA levels in (Ai) non-activated (NA) and activated T cells (36 hr, left panel) and in sorted IFN-γ^+^, IFN-γ^+^IL-10^+^, and IL-10^+^ Th1 cell subpopulations (activated for 36 hr, right panel), and (Aii) ratio of CYT-1 to CYT-2 tail mRNA expression. (B–D) Nutrient channel expression, mTORC1 activity and glycolysis and OXPHOS levels in IFN-γ^+^, IFN-γ^+^IL-10^+^, and IL-10^+^ Th1 cell subpopulations. Data in (A)–(D) are derived from n = 3. ^∗^p < 0.05; ^∗∗^p < 0.01. See also Figure S6.

Resting CD4^+^ T cells expressed all four isoforms of CD46: BC1, C1, BC2, and C2 (Figure S1H). Upon activation, however, the isoform expression pattern changed, with an increase in CYT-1-bearing forms (i.e., BC1 and C1) (Liszewski et al., 2013) (Figure 7Ai). Furthermore, several studies have implicated CYT-1 of CD46 as a Th1 cell “driver” (Le Friec et al., 2012; Ni Choileain et al., 2011). Because this parallels our observation that specifically CYT-1 of CD46 was required for mTORC1 activation, we hypothesized that CYT-1 versus CYT-2 expression is different in IFN-γ^+^, IFN-γ^+^IL-10^+^, IL-10^+^, and IFN-γ^–^IL-10^–^ subpopulations and that expression changes with their progression through the Th1 cell life cycle. To address this, we sorted the Th1 cell subpopulations resulting from CD3+CD46 activation and assessed respective CD46 mRNA CYT-1 versus CYT-2 expression patterns. CYT-1 expression increased over CYT-2 expression in IFN-γ^+^ and IFN-γ^+^IL-10^+^ populations, while IL-10^+^ T cells switched back toward a CYT-2 predominant profile (Figures 7Ai and 7Aii). Moreover, the expression of GLUT1, LAT1, and LAMTOR5 and the phosphorylation of mTOR and p70S6K, as well as OXPHOS and glycolysis levels, all paralleled the expression kinetics of CYT-1—with all being also increased in IFN-γ^+^ and IFN-γ^+^IL-10^+^ subsets, while returning to basal levels in IL-10^+^ and IFN-γ^–^IL-10^–^ cells (Figures 7B–7D).

These results demonstrated that the temporal changes in CD46 isoform expression induced upon T cell activation mediated the metabolic events specific to the induction, effector function, and contraction phases of Th1 cells and, thus, demarcated the human Th1 cell life cycle phases (Figures S6A and S6B).

## Discussion

Glucose metabolism, OXPHOS, AA influx, and differential activation of the metabolic checkpoint kinase mTOR each play important roles in enabling successful T cell immunity (Jones and Thompson, 2007; MacIver et al., 2013; Pollizzi and Powell, 2014). However, in humans the receptor(s) triggering, and the molecular events mediating, distinct immune-metabolic activities in vivo are not well defined. Here we provide a molecular framework that integrates the complement receptor CD46 with metabolic reprogramming required for human Th1 cell induction, and we demonstrate dysregulation of this key metabolic program in patients with reduced CD46 expression. We suggest a model in which TCR activation, which induces the local generation of CD46 ligand C3b (Liszewski et al., 2013), increases expression of CD46 isoforms bearing CYT-1. CD46 CYT-1-driven signals then mediate upregulation of GLUT1 and, more importantly, LAT1, allowing for increased glucose and AA influx into the cell. Increased expression of LAMTOR5 and assembly of the lysosome-based machinery simultaneously enables AA sensing via mTORC1. mTORC1 activation and downstream events, including further induction of glycolysis and OXPHOS, then support Th1 cell maturation and IFN-γ production. During Th1 cell contraction and induction of IL-10 coexpression, CD46 isoform expression of CD4^+^ T cells reverts to a CYT-2 predominant pattern, accompanied by reduced expression of GLUT1 and LAT1, and downregulation of OXPHOS and glycolysis. This model aligns with the facts that the *SLC2A1* gene is hypermethylated in patient CD46-3; that high GLUT1 expression is selectively required for effector but not regulatory T (Treg) cell responses (Macintyre et al., 2014); that Treg cells do not upregulate CD46 CYT-1 upon activation, and that Treg cell numbers and functions are normal in CD46-deficient patients (Liszewski et al., 2013).

A complement receptor or regulator serving as the murine “CD46 homolog” with regard to Th1 cell regulation has not been identified, and published work indicates that CD28 drives glycolysis and OXPHOS as required for effector T cells function in mice. CD28 can also regulate GLUT1 expression and glycolysis (Frauwirth et al., 2002; Jacobs et al., 2008) and LAT1 induction (Hayashi et al., 2013) in activated human CD4^+^ T cells. However, because costimulation via CD28 potentiates TCR-induced autocrine generation of C3b, and CD28 signals are not sufficient to induce Th1 cell responses in CD46-deficient T cells, increased autocrine CD46 engagement plays a key role in the CD28-driven nutrient uptake in CD46-sufficient cells. Nonetheless, a cooperation between CD28- and CD46-intrinsic molecular events jointly supporting optimal Th1 cell induction remains an important possibility, perhaps with CD28 as an upstream regulator of the autocrine “C3-CD46” axis in T cells.

CD4^+^ T cell subsets have distinct metabolic requirements. Effector T cells demand high levels of glycolysis, whereas Treg cells are more dependent on OXPHOS, and mTORC1 activity is required for Th1 and Th17 cell responses, whereas mTORC2 drives Th2 cell function (Delgoffe et al., 2009; Michalek et al., 2011; Shi et al., 2011). Congruent with this, decreased expression of CD46 impacted proportionally on mTORC1 activity and Th1 cell induction, whereas Th2 cell responses remained unaffected. Interestingly, only complete absence of CD46 led to failure of Th17 cell induction, suggesting metabolic threshold differences between induction of Th1 and Th17 cell effector populations. As T cells from CD46-deficient patients proliferate normally (not shown), these differences likely relate to non-bioenergetic aspects of subset-specific metabolic reprogramming. Indeed, recent work shows that expression of the pyruvate dehydrogenase (PDH) kinase 1 in Th17, but not Th1, cells is a hallmark of their distinct metabolic programs (Gerriets et al., 2015). Our observation that cleaved cytoplasmic domains of CD46 translocated to the nucleus makes it a possibility that they function within transcription factor- or regulator-complexes, directly controlling metabolism.

Complement is among the evolutionary oldest effector immune systems and preceded the appearance of B and T cells (Le Friec and Kemper, 2009). The recent discovery that complement activation and function also occurs within cells evokes the possibility that complement evolved initially as an intracellular stress detection system (Kolev et al., 2014). A link between complement and metabolic checkpoint kinases, such as mTOR, is thus plausible. The possibility for such a functional cooperation is further supported by the fact that CD46 is a key constituent of a sensory network integrating signals to respond to variable nutrient environments. For example, Notch regulates OXPHOS and glycolysis in cancer cells, pre-T cells, and memory cells (Ciofani and Zúñiga-Pflücker, 2005; Landor et al., 2011; Maekawa et al., 2015), and we have previously shown that interaction of CD46 and the Notch-family member Jagged1 regulates Th1 cell activation (Le Friec et al., 2012). Furthermore, CD46 facilitates the assembly of the IL-2 and IL-7 receptor complexes in T cells (Le Friec et al., 2012), which contributes to increased GLUT1 expression (Wofford et al., 2008) and integrates “high environmental IL-2” signals into a Th1 cell shutdown program.

Our results may also suggest reevaluating the role of CD46 in infection. CD46 serves as receptor for several pathogens (Cattaneo, 2004) and dogma states that pathogens binding CD46 abuse the receptor’s ability to promote IL-10 switching, thus furthering an infection-promoting environment (Cope et al., 2011). However, recent work demonstrates that, in epithelial cells, adenoviruses induce glycolysis, thereby supporting the metabolic demands of viral replication (Thai et al., 2014). Thus, the interplay of pathogens and CD46 may have a “metabolic dimension,” and understanding the signals regulating *CD46* mRNA splicing and/or autocrine C3b generation may also deliver novel tools to therapeutically exploit CD4^+^ T cell metabolic reprogramming.

## Experimental Procedures

### Donors and Patients

Blood samples were obtained with ethical and institutional approvals (Wandsworth Research Ethics Committee, REC number 09/H0803/154). T cells were purified from buffy coats (NHSBT, Tooting, UK; Blood Donor Centre, Basel, Switzerland) or blood samples from healthy volunteers after informed consent. Three adult CD46-deficient patients with confirmed diagnosis of hemolytic uremic syndrome (HUS) and with clinically low Ig (IgG1 and IgG2) levels and recurrent chest infections (CD46-1 and CD46-3) were recruited in France and blood samples were obtained with local ethical approval (Couzi et al., 2008; Fremeaux-Bacchi et al., 2006; Le Friec et al., 2012). The patients had neither infection nor active hemolytic uremic syndrome at the time of blood sampling. In all experiments that involved T cells from CD46-deficient patients, T cells from age- and sex-matched healthy volunteers were used as controls. For details on T cell isolation, activation, and cytokine measurements, see Supplemental Experimental Procedures.

### Antibodies, Proteins, and Inhibitors

Details are included in the Supplemental Experimental Procedures.

### OCR and ECAR Measurements

For analysis of the OCR (in pMoles/min) and ECAR (in mpH/min), the Seahorse XF^e^-96 (primary cells) or Seahorse XF-24 (Jurkat cell lines) metabolic extracellular flux analyzers were used (Seahorse Bioscience, North Billerica, MA) with detailed instructions in Supplemental Experimental Procedures and metabolic parameters calculated as described in Figure S1.

### Glucose and Amino Acid Uptake Assays

Details are included in the Supplemental Experimental Procedures.

### Confocal Microscopy and Colocalization Analyses

Assays were performed as previously described (Liszewski et al., 2013), with further details in Supplemental Experimental Procedures.

### mRNA Silencing

siRNA targeting human LAMTOR5 (SR307168), CD46 (SR302841), and negative control scrambled siRNA were purchased from Origene (Rockville, MD) and delivered into primary human CD4^+^ T cells by transfection using Lipofectamine RNAiMAX (Life Technologies, Paisley, UK) according to the manufacturer-provided protocol. LAMTOR5 and CD46 protein knockdown were consistently about 70% and between 50%–35%, respectively.

### CD46 Isoform-Specific RT-PCR and Lentiviral Transfection of T Cells with CD46 CYT-1 or CYT-2

Details are included in the Supplemental Experimental Procedures.

### Gene Arrays and Array Analyses

Transcriptome profiling was performed using Illumina HT12V4 microarrays (Illumina, Great Chesterford, UK) on technical triplicates using CD3+CD46-activated T cells isolated from patient CD46-3 and an age- and sex-matched healthy donor. Expression data were analyzed using Partek Genomics Suite (Partek, St. Louis, USA) version 6.6, Ingenuity Pathway Analysis (QIAGEN) and Gene Set Enrichment Analysis, GSEA (Subramanian et al., 2005) (Broad Institute of MIT and Harvard). For details, see Supplemental Experimental Procedures. The raw data of all arrays are deposited with the Gene Expression Omnibus (GEO) repository under the accession number GEO: GSE69090.

### Statistical Analysis

Analyses were performed on GraphPad Prism (La Jolla, CA). Data are presented as mean ± SD or median (interquartile range, IQR) for parametric and non-parametric data, respectively, and compared using paired t tests with Bonferroni correction for multiple comparisons, Wilcoxon signed rank tests, the two-tailed Mann-Whitney test, one-way or two-way ANOVA with a Tukey multiple comparison post hoc test, as appropriate. p values < 0.05 denoted statistical significance throughout.

## Author Contributions

M.K., S.D., and G.L.F. designed and performed GLUT1, LAT1, and LAMTOR5 expression assays, metabolic flux experiments and nuclear translocation experiments and wrote and edited the manuscript. G.A.P. helped with the retroviral transfection “rescue” experiments. A.N. performed the exome sequencing, and G.A. analyzed CD46 and cytokine expression in Jurkat T cells. P.L., G.A., and J.W. performed the gene arrays and P.L. and B.A. analyzed the data and edited the manuscript. M.F., R.B., J.L., L.R., and G.R.B. contributed to metabolic assays. L.C. provided the blood samples from the CD46-deficient patients and discussed the data. C.K and C.H. perceived, designed, and coordinated the study and wrote the manuscript.

## Accession Numbers

The raw data of all arrays are deposited with the Gene Expression Omnibus (GEO) repository under the accession number GEO: GSE69090.

## Supplemental Information

Document S1. Figures S1–S6 and Supplemental Experimental Procedures

Table S1. Related to Figure 3 Whole-exome sequencing was used to screen for mutations in candidate genes known to cause monogenic immune defects (list of genes in supplementary table Table S2). We extracted genomic DNA from peripheral blood from two affected individuals (Cd46-deficient patients CD46-2 [sibling of CD46-1] and CD46-3), performed whole-exome capture by using in-solution hybridization (Agilent All Exon Kit V5) and generated sequencing on the Illumina HiSeq 2000. Resulting reads were aligned to the reference human genome (UCSC Genome Browser hg19, GRCh37) with the Novoalign software package (Novocraft Technologies). Duplicate reads, resulting from PCR clonality or optical duplicates, and reads mapping to multiple locations were excluded from downstream analysis. The only gene containing rare and damaging variants common to both subjects was CD46, as had been previously shown for both unrelated, Caucasian individuals. The respective mutations of patient CD46-2 were heterozygous variants c.175C>T (p.R59X) and c.G104G>A (p.C35Y), and patient CD46-3 had a homozygous variant in c.286+1G>C (RefSeq NM_172359) in exon 2. These mutations were confirmed by Sanger sequencing (not shown). NOTE: Positions with normalized SIFT probabilities less than 0.05 are predicted to be deleterious, those greater than or equal to 0.05 are predicted to be tolerated. Also, columns EVS and 1KG indicate the minor allele frequencies in two large population genetics databases, and all positions observed are not rare and are thus not suggestive of causing a rare immune defect. The seemingly rare mutation in TARP 38299727 occurs very frequently in our inhouse database of normal donors and is therefore also not suggestive of being causal.

Table S2. Related to Figure 3 Shown is a list of genes in which mutations have been known to cause monogenetic immune defects and which have been screened for mutations in the CD46-deficient patients.

Table S3. Related to Figure 4 Gene list of the 403 genes differently expressed between CD3+CD46-activated T cells (2 hr) isolated from Patient CD46 3 and an age- and sex-matched healthy donor. Arrays were performed as technical triplicates.

Table S4. Related to Figure 3 Genes differently expressed in T cells from Patient CD46-3 and a healthy donor identified by GMO that specifically functioning in metabolic processes of the cells.

Document S2. Article plus Supplemental Information
