# Supplementary material for: Complement Regulates Nutrient Influx and Metabolic Reprogramming during Th1 Cell Responses
Source: Immunity. 2015 Jun 16;42(6):1033–47. doi: 10.1016/j.immuni.2015.05.024 (PMC4518498; doi:10.1016/j.immuni.2015.05.024)
Supplement: Document S1. Figures S1–S6 and Supplemental Experimental Procedures [file mmc1.pdf]

Immunity

Supplemental Information

Complement Regulates Nutrient Influx and Metabolic Reprogramming during Th1 Cell Responses

Martin Kolev, Sarah Dimeloe, Gaelle Le Friec, Alexander Navarini, Giuseppina Arbore, Giovanni A. Povoleri, Marco Fischer, Reka Belle, Jordan Loeliger, Leyla Razik, Glenn R. Bantug, Julie Watson, Lionel Couzi, Behdad Afzali, Paul Lavender, Christoph Hess, and Claudia Kemper

Figure S1, related to Figure 1

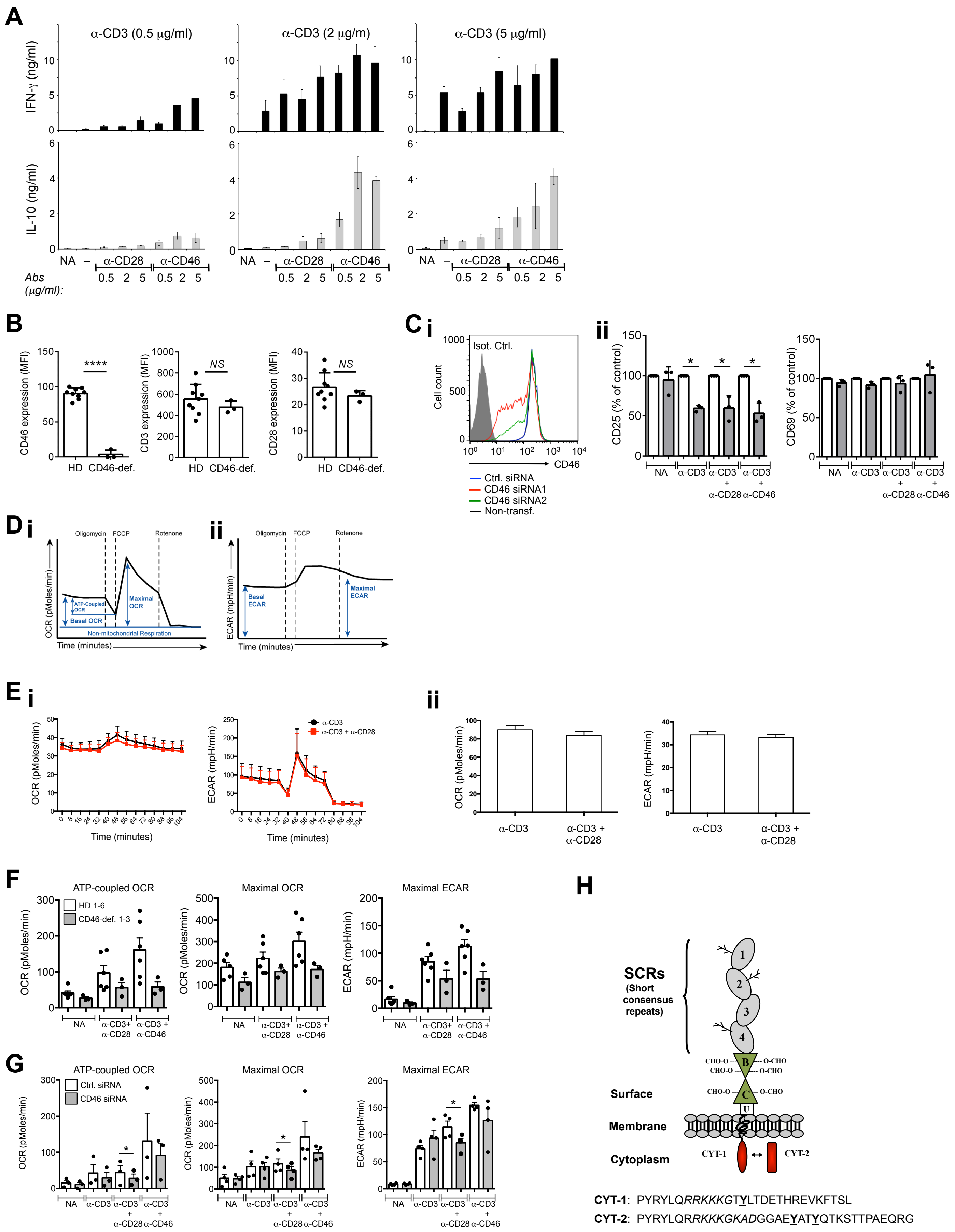

CYT-1: PYRYLQ*RRKKKG*TYLTDETHREVKFTSL

CYT-2: PYRYLQR*RKKKGKAD*GGAEYATYQTKSTTPAEQRG

Figure S1, related to Figure 1. CD46 isoforms bearing cytoplasmic domain 1 are required for Th1 induction (IFN-γ secretion) in CD4$^+$ T cells. (A) TCR and CD28-induced Th1 cytokine production correlates with CD46 ligand generation (Figure 1A). Effects of anti-CD3 and anti-CD28 antibody titration on IFN-γ production and IL-10 switching in T cells from healthy donors. Freshly purified CD4$^+$ T cells were either left non-activated (NA) or activated by plate immobilized Abs as depicted and cytokine production measured at 36 h post activation. Shown are results ± SD obtained in n = 3 independently performed experiments using a different donor each time. (B) TCR (CD3), CD28 and CD46 expression levels on T cells isolated from nine age- and sex-matched healthy donors (HD) and from CD46-deficient patients CD46-1, -2 and -3. Patient CD46-1 and CD46-2 had 10% of normal CD46 expression levels on T cells and surface CD46 expression was undetectable in patient CD46-3. (C) Reduced CD46 protein expression in T cells from HDs leads to a significant decrease in CD25 expression upon activation. Purified T cells were (Ci) transfected with a CD46-specific siRNA or a scrambled control siRNA and (Cii) CD25 and CD69 expression measured at 36 h post activation. Shown are results ± SD obtained in n = 4 independently performed experiments. (D) Schematic diagram of oxygen consumption rate (OCR) and extracellular acidification rate (ECAR) profiles as generated by the Seahorse extracellular flux analyzer. (D, left panel) Schematic OCR time course under basal conditions and following perturbation of mitochondrial respiration with oligomycin, FCCP and rotenone. Using this perturbation profiling technique, four OCR rates are directly measured: the non-corrected basal OCR [OCR(basal-nc)], the rate following inhibition of ATP synthase [OCR(oligomycin)], the peak rate following mitochondrial uncoupling [OCR(peak-FCCP)], and the rate following inhibition of mitochondrial respiration [OCR(rotenone)]. The following respiratory parameters (indicated by blue double-ended arrows in the diagram) are calculated using the formulas below:

(1) basal respiration = [OCR(basal-nc)] – [OCR(rotenone)]

(2) ATP coupled respiration = [OCR(basal-nc)] – [OCR(oligomycin)]

(3) maximal respiratory capacity = [OCR(peak-FCCP)] – [OCR(rotenone)]

(D, right panle) Schematic ECAR time course under basal conditions and following inhibition of mitochondrial respiration. Basal ECAR is the initial rate measured by the extracellular flux analyzer. The maximal ECAR is the rate following addition of rotenone. Blue double-ended arrows in the diagram indicate the respective glycolytic parameters. (E) Oxidative phosphorylation (OCR) and glycolysis (ECAR) rates in T cells activated with anti-CD3 or anti-CD3 and anti-CD28 mAb for 36 h. The right panel depicts basal OCR and ECAR. Data shown

are mean ± SD derived from three donors (n = 3). (F) ATP coupled and maximal OCR and maximal ECAR in non-activated and activated $CD4^+$ T cells isolated from CD46-deficient patients CD46-1, -2, and -3 compared to T cells from six HDs, as assessed using the XF Seahorse Analyzer and calculated as described in S1D. (G) Experiments performed as under (F) but using T cells from four different HDs treated with either control siRNA or CD46-specific siRNA. (H) Schematic of the CD46 protein structure. The four major isoforms of CD46 are generated by alternative splicing of the extracellular 'BC' region (either 'C' or 'BC') and the intracellular domains (CYT-1 or CYT-2) of an mRNA transcribed from a single gene. The amino acid sequence for CYT-1 and CYT-2 is shown below with putative nuclear targeting signals depicted with italic letters and tyrosine phosphorylation sites within CYT-1 and CYT-2, 'Y', marked in bold. Candidate kinases include casein kinase 2 (CK-2) and protein kinase C (PKC) for CYT-1, and Src kinases and CK-2 for CYT-2. *, $p < 0.01$; ****, $p < 0.001$; NS, not statistically significant.

Figure S2, related to Figure 2

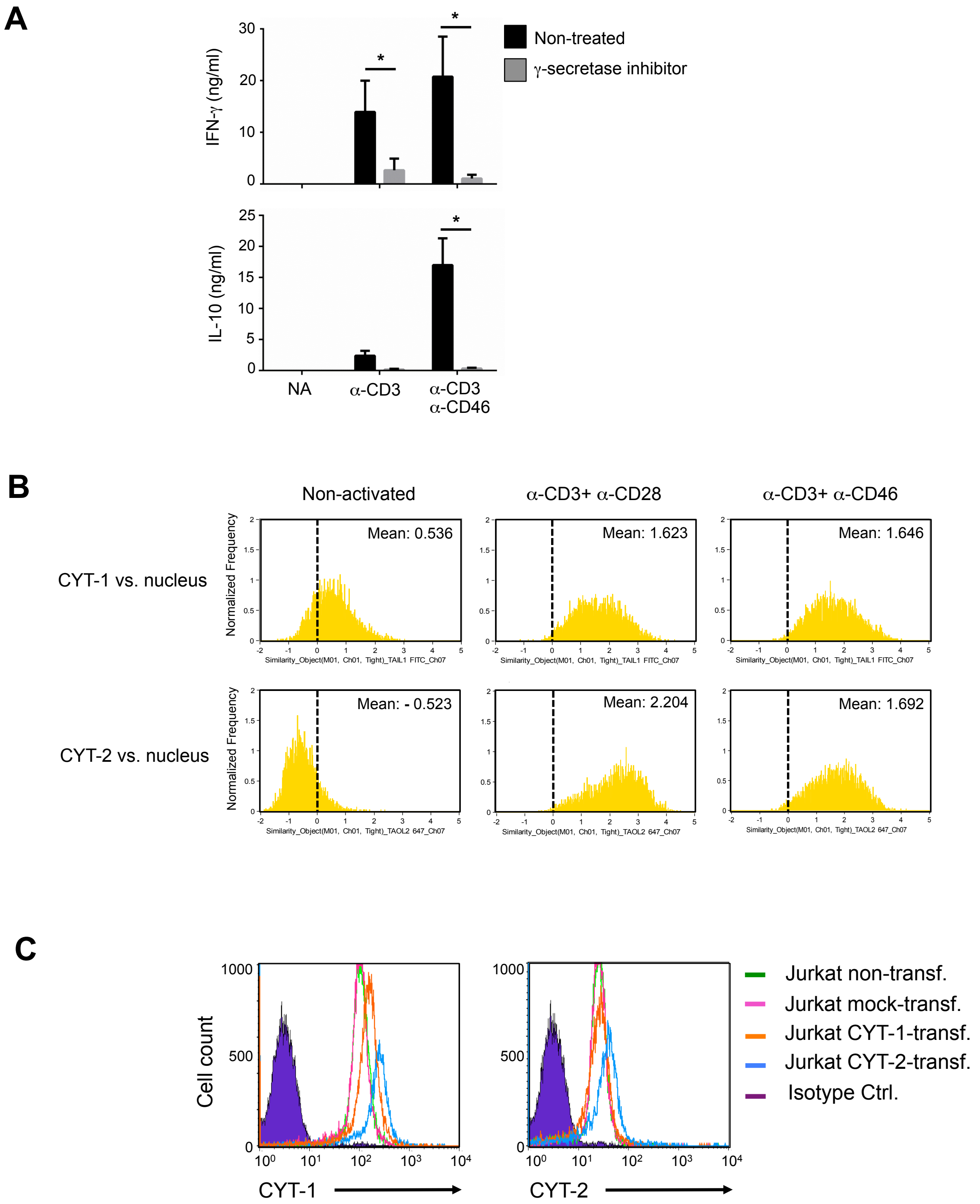

Figure S2, related to Figure 2. CD46 function requires processing and nuclear translocation of its intracellular domains. (A) Inhibition of γ-secretase activity prevents Th1induction. $CD4^+$ T cells were activated as depicted with or without addition of a γ-secretase inhibitor (100 nM) and cytokine production measured 36 h post activation. Shown are data ± SD derived from three (n = 3) independently performed experiments. (B) CD46 nuclear domains CYT-1 and CYT-2

translocate into the nucleus upon T cell activation. T cells were left non-activated (NA) or activated as indicated for 2 hrs, permeabilized and stained with CYT-1- and CYT-2-specific antibodies and nuclear translocation of the CD46 tails assessed using Image Stream. FACS plots present correlations between CYT-1 and CYT-2 pixel intensity and intensity of nuclei as measure of colocalization. Shown is one representative data set of n = 3 similarly performed experiments. (C) CYT-1 and CYT-2 expression in non-activated Jurkat T cells 48 h post transfection with retrovirus expressing either CYT-1 or CYT-2 (n = 3). *, $p < 0.05$.

Figure S3, related to Figure 3

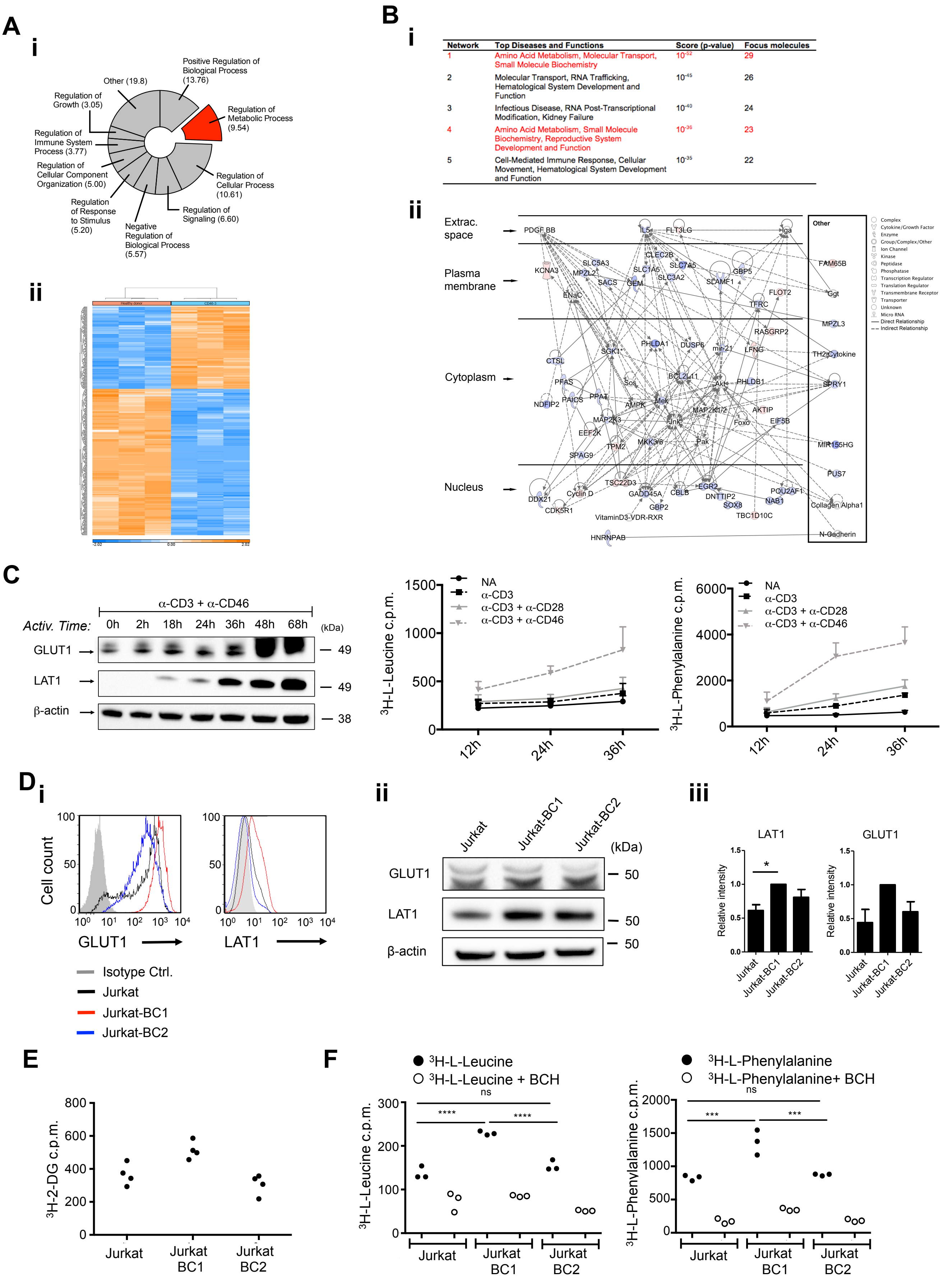

Network	Top Diseases and Functions	Score (p-value)	Focus molecules
1	Amino Acid Metabolism, Molecular Transport, Small Molecule Biochemistry	10^{-52}	29
2	Molecular Transport, RNA Trafficking, Hematological System Development and Function	10^{-45}	26
3	Infectious Disease, RNA Post-Transcriptional Modification, Kidney Failure	10^{-40}	24
4	Amino Acid Metabolism, Small Molecule Biochemistry, Reproductive System Development and Function	10^{-36}	23
5	Cell-Mediated Immune Response, Cellular Movement, Hematological System Development and Function	10^{-35}	22

Figure S3, related to Figure 3. CD46 CYT-1 mediates glucose and amino acid influx in $CD4^+$ T cells via GLUT1 and LAT1. (A-B) Analysis of 403 differentially expressed genes between healthy donor (HD) and patient CD46-3, showing (Ai) GO enrichment scores for GO terms under "Regulation of Biological Process" (GO ID 50789) - highlighted is "Regulation of Metabolic Process" (GO ID 19222; p<0.01), (Aii) heatmap of genes in the highlighted segment of (Ai) and (B) Ingenuity Pathway Analysis (IPA) output showing (Bi) the top 5 gene networks and (Bii) schematic of merged gene networks 1 and 4, which are functionally associated with amino acid metabolism (Bi). (C) Time course of GLUT1 and LAT1 expression and amino acid uptake in activated T cells. Purified T cells were activated with antibodies to CD3 and CD46. At indicated time points, expression of GLUT1 and LAT1 was measured by Western blotting (left panel) and amino acid uptake was assessed (right panel). The Western blot is representative of three independently performed experiments and data shown in the lower panel are mean ± SD derived from n = 3 different donors. (D) Expression of GLUT1 and LAT1 in resting Jurkat, Jurkat-BC1 and Jurkat-BC2 cells assessed by (Di) FACS analysis and by (Dii) Western blotting, with (Diii) densitometric analyses of the band intensities obtained in (Dii). Data shown are representative of n = 3 independently performed experiments. (E and F) CD46-BC1 over-expression increases glucose and amino acid uptake in Jurkat T cells. Jurkat cells, Jurkat-BC1, and Jurkat-BC2 cells were incubated with (E) tritium labeled 2-deoxyglucose (^{3}H 2-DG) or (F) L-Leucine (^{3}H L-Leucine) or L-Phenylalanine (^{3}H L-Phenylalanine) in the presence or absence of the LAT1 inhibitor BCH and glucose and amino acid uptake measured, respectively. Data shown are from four (E, n = 4) or three (F, n = 3) experiments. *, $p < 0.05$; *** $p < 0.005$; ****, $p < 0.001$; ns, statistically not significant.

Figure S4, related to Figure 4

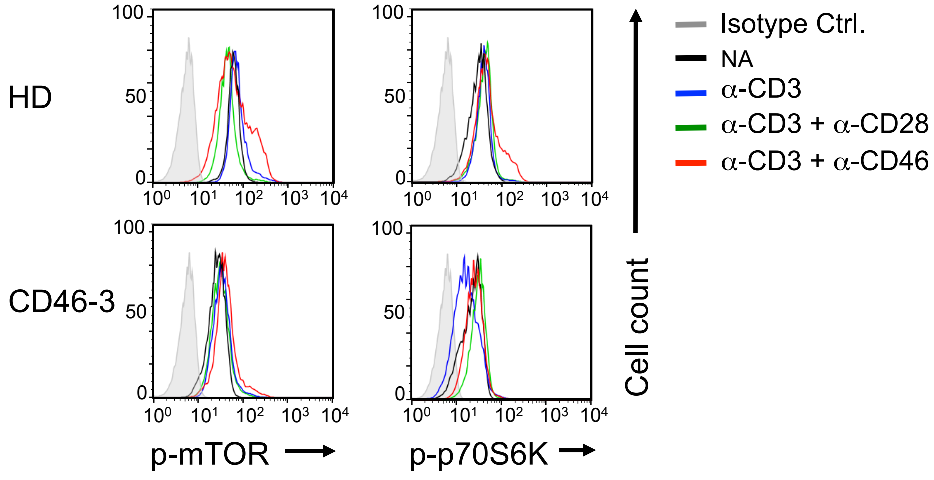

Figure S4, related to Figure 4. CD46 is required for normal p70S6K phosphorylation in activated T cells. Abundance of phosphorylated mTOR (p-mTOR) and p70S6K (p-p70S6K) in non-activated (NA) and activated T cells isolated from four different healthy donors (HD1-4) and from patient CD46-3 at 36 h post activation.

Figure S5, related to Figure 5

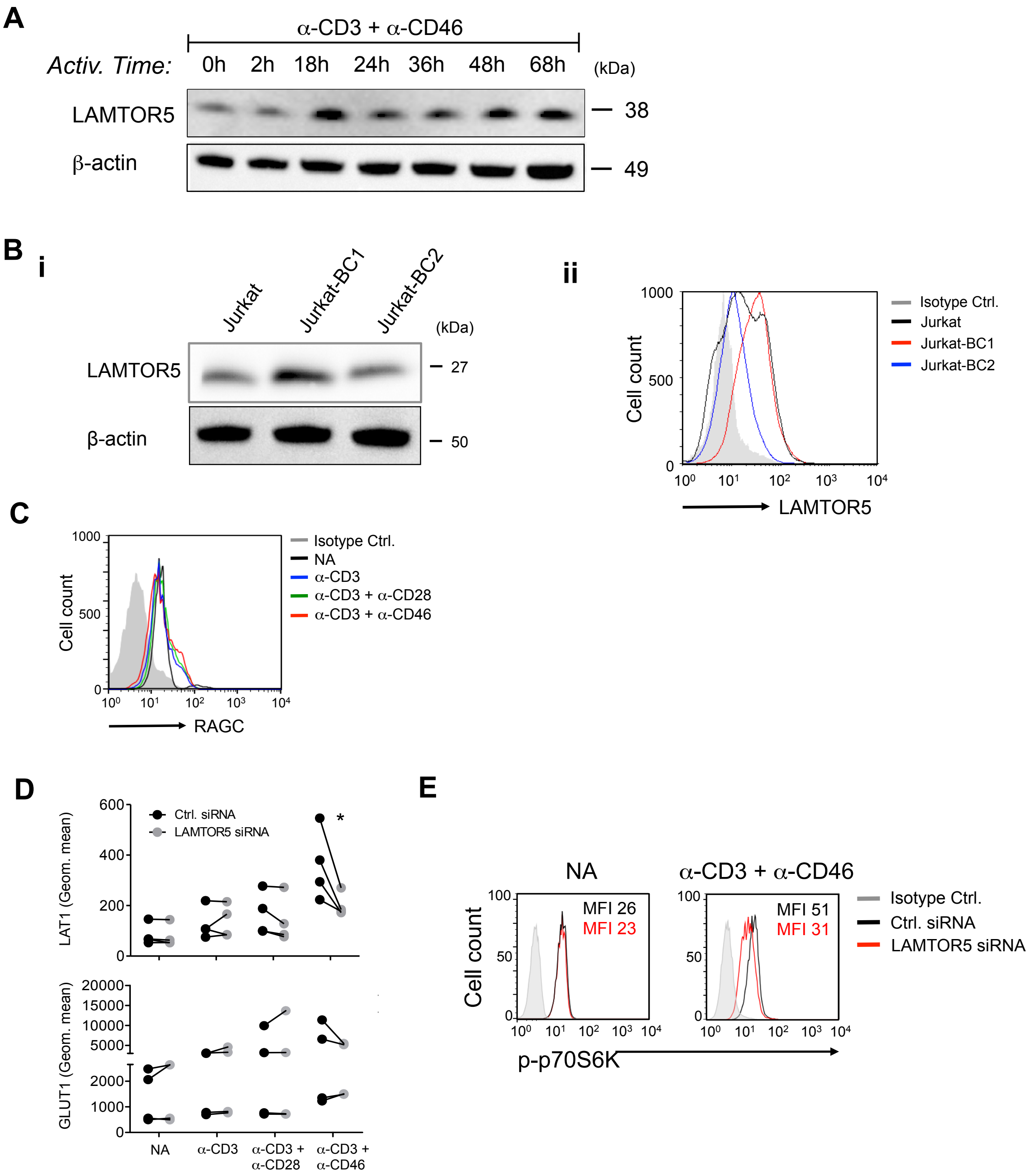

Figure S5, related to Figure 5. Activation-induced LAMTOR5 expression is required for TORC1-mediated p70S6K phosphorylation. (A) Time course of LAMTOR5 expression in activated CD4^{+} T cells. Purified human CD4^{+} T cells were activated with antibodies to CD3 and CD46, and LAMTOR5 expression analyzed by Western blotting at indicated time points. Shown is one representative result of n = 3 similarly performed experiments. (B) Comparison of LAMTOR5 expression in resting Jurkat, Jurkat-BC1 and Jurkat-BC2 cells assessed by (Bi)

Western blotting, and by (Bii) FACS analysis. Data shown are representative of n = 3 independently performed experiments. (C) Expression of RAGC in activated T cells at 36 h post activation. Shown is one representative result of n = 2 similarly performed experiments. (D and E) Effect of reduction of LAMTOR5 protein expression by RNA silencing technique on (D) GLUT1 and LAT1 expression and (E) p70S6K phosphorylation in CD3+CD46-activated primary T cells 36 h post activation Data shown are representative of n = 3 or n = 4 independently performed experiments.

Figure S6, related to Figure 7

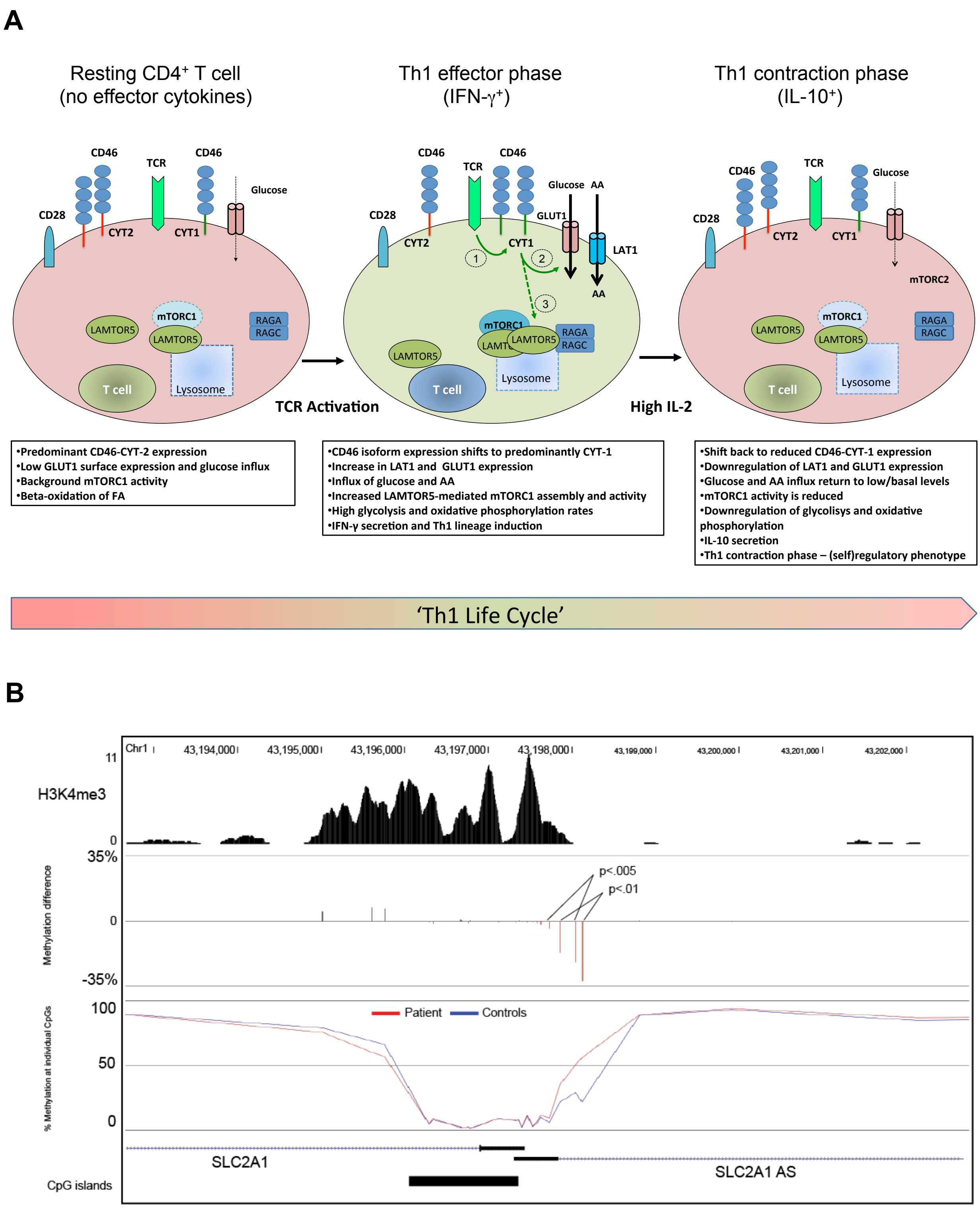

Figure S6, related to Figure 7. The role of CD46 isoforms in the metabolic switch during the Th1 life cycle. (A) Model of the CD46-mediated contributions to key metabolic events required for normal Th1 effector induction. T cell receptor activation of resting T cells induces the local generation of CD46 ligand C3b (not shown) as well as (1) increased expression of CD46 isoforms bearing CYT-1. CD46 CYT-1-driven signals then lead to (2) upregulation of the

glucose transporter GLUT1 and, more importantly, the amino acid channel LAT1, allowing for increased influx of glucose and amino acids into the cell. In parallel, CD46-activated T cells induce increased expression of LAMTOR5 either via CD46-mediated direct or indirect signals (indicated by dotted arriws), and via this assembly of the lysosome-based machinery enabling amino acid sensing via mTORC1, which then leads to key downstream events such as the phosphorylation of p70S6K and induction of glycolysis and oxidative phosphorylation (OXPHOS) required for IFN-γ production. During 'Th1 contraction' and induction of IL-10 co-expression, CD46 isoform expression reverts to a CYT-2 predominant pattern, accompanied by reduced expression of GLUT1 and LAT1, and downregulation of glycolysis and OXPHOS. (B) Increased DNA methylation around the SLC2A1/SLC2A1-AS (GLUT1) bidirectional promoter in CD46-deficient patient CD46-3. DNA methylation was analyzed using bisulphite conversion of cytosine residues within genomic DNA isolated from resting T effector cells from three healthy controls and from T cells collected from CD46-3 at two different times (one year apart). Methylation was quantified on the Illumina Infinium 450K platform. A cluster of CpG dinucleotides showed differential methylation (cg06792911, 7% Control and 11% Patient, $p<0.005$; cg12656391, 18% Control and 35% Patient, $p<0.01$; cg00102166, 27% Control and 50% Patient, $p<0.005$; cg09824328, 22% Control and 54% Patient, $p<0.01$) and coincided with the SLC2A1/SLC2A1-AS bidirectional promoter, the extent of which was marked with H3K4me3 in ChIPseq experiments using mononucleosomal chromatin from polarized primary T cells.

SUPPLEMENTAL EXPERIMENTAL PROCEDURES

Human CD46-transgenic mice

Mice transgenic for human CD46 were previously generated using a YAC containing the human *CD46* gene (Mrkic et al., 1998) with confirmed human CD46-like expression on all assessed nucleated cells including lymphocytes (Kemper et al., 2001).

Antibodies, proteins and inhibitors. Cell-stimulating mAbs were bought from BD Biosciences, San Diego, CA (anti-CD28, CD28.2), purified from a specific hybridoma (anti-CD3; OKT-3) or generated in-house (anti-CD46; TRA-2-10 (Wang et al., 2000). The antibodies to CD46 CYT-1 and CYT-2 were a gift from Dr. Maggie So from the University of Arizona (Weyand et al., 2006), the rabbit polyclonal anti-CD46 serum used in confocal microscopy studies was generated in-house, the PE-anti CD46 (12-0469-42) from eBiosciences (San Diego, CA) and anti-CD69 (561932), anti p-ERK1/2 (612359) and anti-CD25 (555434) from BD. Monoclonal antibodies to LAMP1 (ab24170), GLUT1 (ab115730), HBXIP/LAMTOR5 (ab157480) and β-actin (ab8226) were purchased from Abcam (Cambridge, UK), as was the polyclonal anti-serum to the green fluorescence protein (GFP, ab290). Antibodies recognizing phosphorylated mTOR (2971S), phosphorylated p70S6 Kinase (9205S), human RAGC (3360), Akt phosphorylated at serine 473 (4058), LAT1 (5347) and the mTOR Regulation Antibody Sampler Kit (9864) and the NF-κB Antibody Sampler Kit (9936S) were bought from Cell Signaling Technology (Danvers, MA). mTOR (7C10) conjugated to Alexa Fluor 647 used in confocal microscopy experiments was obtained from Cell Signaling (5048), while the anti-RAGC conjugated to Alexa Fluor 488 (ABIN914033) was from Antibodies Online (Aachen, Germany). Anti-Rabbit IgG (H+L chain Alexa Fluor 594) was from Abcam (ab150076), while anti-mouse IgG Alexa Fluor 488 (A11001), anti-mouse IgG Alexa Fluor 594 (A21125), anti-Rabbit IgG (H+L) Alexa Fluor 488 (A11034), anti-Rabbit IgG (H+L) Alexa Fluor 594 (A11037) and RPE-conjugated anti-Rabbit F(ab')2 fragment (A10542) were obtained from Molecular Probes/Life Sciences (Paisley, UK). Rapamycin was a gift from Dr Cristiano Scotta, King's College London, UK. The amino acid transport inhibitor BCH (2-Amino-2-norbornanecarboxylic acid, A7902), the 2-DG glucose analogue (2-Deoxy-D-glucose, D6134) and the γ-secretase inhibitor (L-685,458) were purchased from Sigma-Aldrich (Saint Louis, MO).

NF-κB reporter system

Jurkat T cells were cotransfected with a luciferase NF-κB reporter plasmid and a renilla control plasmid (E8491; Promega, Madison, WI) for normalization of transfection efficiency between samples. Cells were activated with different stimulating antibodies and luciferase activity assessed in cell lysates 1 - 3 h post activation using the Dual-Luciferase Reporter Assay System (E1919) from Promega.

T cell isolation and activation. PBMCs were separated to $CD4^+$ T cells using the MACS human $CD4^+$ Positive T cell Isolation Kit (Miltenyi Biotech Ltd, Bisley, UK) according to manufacturer's instructions. Purity of isolated lymphocyte fractions was typically >97 %. $CD4^+$ T cells were activated in 48-well culture plates (2.5 – 3.0 x 10^5 cells/well) coated with mAbs to CD3, CD28 or CD46 (2.0 μg/ml PBS each) and addition of 25 U/ml rhIL-2. Cell viability and/or apoptosis was monitored by FACS analysis using Annexin V-APC and Propidium Iodide (both from BD Biosciences, Oxford, UK) staining. The Jurkat T cell lines stably transfected with either the CD46 CYT-1 or CYT-2-bearing isoform have been described previously (Cardone et al., 2010).

Cytokine measurements. Cytokine production by T cells was measured using either the Th1/Th2/Th17 Cytokine Bead Array (560484, BD Biosciences) or the the Human Cytokine Secretion Assay Kits for IFN-γ (130-090-433) and IL-10 (130-090-761) from Miltenyi (Surrey, UK) in combination. IL-8 was measured using the Human IL-8 ELISA Kit (555244) from BD.

OCR and ECAR measurements. For analysis of the OCR (in pMoles/min) and ECAR (in mpH/min), the Seahorse XF-96 (primary cells) or Seahorse XF-24 (Jurkat cell lines) metabolic extracellular flux analyzers were used (Seahorse Bioscience, North Billerica, MA). $CD4^+$ T cells were resuspended in serum-free unbuffered RPMI-1640 medium (R1383, Sigma-Aldrich) and were plated onto Seahorse cell plates (Primary Cells: 2.5 × 10^5 cells/well; Jurkat cell lines: 1.0 x 10^5 cells/well) coated with Cell-Tak (CB-40241, Corning, Reinach, Switzerland) to enhance T cell attachment. Perturbation profiling of the use of metabolic pathways by $CD4^+$ T cells was achieved by the addition of oligomycin (O4876) (1 μM), Carbonyl cyanide-*4*-(trifluoromethoxy)phenylhydrazone (FCCP, C2920) (2 μM) and rotenone (R8875) (1 μM; all from Sigma-Aldrich). Metabolic parameters were then calculated as described in Supplementary Figure 1.

Glucose and amino acid uptake assays. For analysis of glucose and amino acid uptake, 2×10^5 CD4$^+$ T cells were resuspended in 200 µl Hank's balanced-salt solution (Gibco) containing either [^{3}H]2-deoxyglucose (NET549, Perkin Elmer, Schwerzenbach, Switzerland), [^{3}H]L-phenylalanine (MT1916) or [^{3}H]L-leucine (MT672) (both Hartmann Analytic, Germany), all at a final concentration of 1 µCi/rxn (5 µCi/ml). Unlabeled 2-deoxyglucose (10 mM, Sigma-Aldrich) or LAT1 inhibitor 2-aminobicyclo-(2,2,1)-heptane-2-carboxylic acid (BCH, 10 mM, Sigma-Aldrich) were included where indicated. Uptake was assayed for 6 min after which the cells were washed twice with ice-cold PBS. Cells were then lysed with 50 µl 0.1 % Sodium dodecyl sulfate (SDS) and the cell lysate (40 µl) combined with 160 µl MicroScint™ 40 scintillation fluid (Perkin Elmer, Waltham, MA). β-radioactivity was measured by liquid scintillation counting in a TopCount Scintillation Counter (Perkin Elmer).

Glucose uptake capacity was also measured in primary cells via uptake of the fluorescent 2-deoxy-D-glucose analog 2-[N-(7-nitrobenz-2-oxa-1,3-diazol-4-yl)amino]-2-deoxy-D-glucose (2-NBDG, Life Technologies, Zug, Switzerland). Briefly, 2 x 10^5 CD4$^+$ T cells were resuspended in 200 µl RPMI/10 % FCS containing 20 µM 2-NBDG. The cells were then incubated for 20 min at 37 °C and 5 % CO_2 prior to washing twice with FACS buffer. Fluorescence at 540 nm was then analysed using the Accuri Flow Cytometer (BD Biosciences).

Confocal Microscopy and colocalization analyses. Fixed and permeabilized cells were stained with the indicated primary antibodies overnight at 4 °C at the manufacturer's suggested dilutions. For mTOR, LAMTOR5 and LAMP1 or RAGC co-staining, cells were first stained with anti-LAMTOR5 or LAMP1 antibodies followed by appropriate secondary antibodies conjugated to Alexa Fluor 488 and 594. After two washes, anti-mTOR antibody conjugated to Alexa Fluor 647 or anti-RAGC conjugated to Alexa Fluor 488 was added for 1 h at room temperature. Where indicated, staining with secondary antibodies was performed for 1 h at room temperature. Cells were mounted using Vectashield mounting media containing DAPI (H-1500, Vector Laboratories, Peterborough, UK) and images were obtained in the KCL Nikon Imaging Centre by confocal fluorescence microscopy with A1R SI Confocal Microscope (360 objective) both from Nikon (Surrey, UK). Pearson's Correlation Coefficient was calculated with NIS Elements software version 4.03 (Nikon). At least ten layers in 3D plane were scanned for each sample and for all samples a cropped image of a minimum of 10 cells was used to determine a total of 5 colocalization coefficients. Median values for all layers and cells were calculated and used to plot Pearson's Correlation Coefficient. Experiments were performed at least five times with cells from a different healthy donor each time.

ImageStream analysis

Fixed and permeabilized cells were stained with 10 υg/ml of anti-CD46 cytoplasmic tails antibodies CYT1 (2F1) and CYT2 (13G10) directly labelled with DyLight488 and Fluoprobe647H, respectively (Lightning-Link Rapid kits, Innova Bioscience Ltd., Cambridge, UK) for 1 h at room temperature and washed twice. Cells were resuspended in 100 ul of FACS buffer and DAPI added prior to acquisition and analysis on ImageStream^{X} MARKII (using Inspire and Ideas Softwares, both Amnis Corporation, Seattle, WA). Quantitative analyses are based on the acquisition of 10,000 cell events, and MFIs were measured and calculated using an object mask to detect intracellular staining only.

Whole exome deep sequencing

Whole-exome sequencing was used to screen for mutations in candidate genes known to cause monogenic immune defects (list of genes in supplementary Table S2). We extracted genomic DNA from peripheral blood from two affected individuals (CD46-deficient patients CD46-2 [sibling of CD46-1] and CD46-3), performed whole-exome capture by using in-solution hybridization (Agilent All Exon Kit V5) and generated sequencing on the Illumina HiSeq 2000. Resulting reads were aligned to the reference human genome (UCSC Genome Browser hg19, GRCh37) with the Novoalign Software Package (Novocraft Technologies, Selangor, Malaysia). Duplicate reads, resulting from PCR clonality or optical duplicates, and reads mapping to multiple locations were excluded from downstream analysis. The only gene containing rare and damaging variants common to both subjects was CD46, as had been previously shown for both unrelated, Caucasian individuals. The respective mutations of patient CD46-2 were heterozygous variants c.175C>T (p.R59X) and c.G104G>A (p.C35Y), and patient CD46-3 had a homozygous variant in c.286+1G>C (RefSeq NM_172359) in exon 2. These mutations were confirmed by Sanger sequencing (not shown). Positions with normalized SIFT probabilities less than 0.05 are predicted to be deleterious, those greater than or equal to 0.05 are predicted to be tolerated. Also, columns EVS and 1KG indicate the minor allele frequencies in two large population genetics databases, and all positions observed are not rare and are thus not suggestive of causing an immune defect. The seemingly rare mutation in TARP 38299727 occurs very frequently in our in house database of normal donors and is therefore also not suggestive of being causal.

RT-PCR. Primers used to quantify *CD46* mRNA transcription patterns depicting all four isoforms in one reaction are as follows: (F) 5'-GTG GTC AAA TGT CGA TTT CCA GTA GTC

G-3’ and (R) 5’-CAA GCC ACA TTG CAA TAT TAG CTA AGC CAC A-3’ (Wang et al., 2000). *ACTB* (β-actin) control, (F) 5’-AGC ACA GAG CCT CGC CTT T-3’ and (R) 5’-CAC GCA GCT CAT TGT AGA AG-3’. PCR reactions were performed using the QIAGEN OneStep RT-PCR Kit (210212 Manchester, UK) according to the manufacturer’s protocol and with the specific annealing temperatures/times calculated for each primer pair.

Lentiviral transfection of T cells with CD46 CYT-1 or CYT-2. For CD46 cytoplasmic tails overexpression in human T cells, an appropriate Lentivirus was generated by cotransfecting HEK293T cells with packaging plasmid psPAX2 (Addgene, Cambridge, MA), envelope plasmid pMD2.G (Addgene), and pCDH-CMV-MCS-EF1-copGFP (System Biosciences, San Diego, CA) vector containing either exon 13 of CD46 (CYT1) or exon 14 of CD46 (CYT2) in presence of 0.25 M $CaCl_2$. After 48 h and 72 h incubation, media were harvested, filtered and viral particles were precipitated using PEG-it TM Virus Precipitation Solution (System Biosciences, Mountain View, CA), as per manufacturer’s protocol. Viral particles were then added in serial dilutions to $CD4^+$ T cells that had been isolated from Patient CD46-3 and cells were seeded at 1 x 10^5 cells/well into 96-well plates coated with antibodies to CD3 and CD28. Virus-infected cells were monitored by FACS analyses for GFP-positivity at x d post transfection and cytokines measured at indicated time points.

Gene arrays and array analyses. Transcriptome profiling was performed using Illumina HT12V4 microarrays (Illumina Inc., Great Chesterford, UK). RNA was isolated from 10^6 resting $CD4^+$ T cells, along with $CD4^+$ T cells treated with anti-CD3 and anti-CD46 for 2 h from CD46-deficient Patient CD46-3 and an age- and sex-matched healthy donor using a Qiagen Allprep Kit according to the manufacturer’s instructions (80004, Qiagen). Potential genomic DNA contamination was removed by Turbo DNase treatment (AM2238, Ambion/Life Technologies). Technical triplicates of gene expression analysis was conducted using 5 pg total RNA per sample which was labelled and amplified using Ovation Pico WTA System V2 and Encore BiotinIL Kits (NuGen Inc., San Carlos, CA) to generate cDNA. This cDNA was hybridized to Illumina HT12V4 Microarrays which were then washed and scanned using an Illumina iScan System and subjected to quantile normalization within the Genome Studio Suite v1.0 (Illumina).
Expression data were analyzed using Partek Genomics Suite (Partek Inc., St Louis, USA) version 6.6, Ingenuity Pathway Analysis (Qiagen) and Gene Set Enrichment Analysis, GSEA (Subramanian et al., 2005) (Broad Institute of MIT and Harvard). Differentially expressed genes were defined as those who expression was significantly different ($p<0.05$) between patient CD46-

3 and healthy donor (HD) by at lest 1.5 fold. GSEA compared RPKM values of HD to patient CD46-3 for enrichment of the Gene Ontology gene set “Regulation of Metabolic Process”, using log2 ratio of classes and 10,000 permutations.

SUPPLEMENTAL REFERENCES

Cardone, J., Le Friec, G., Vantourout, P., Roberts, A., Fuchs, A., Jackson, I., Suddason, T., Lord, G., Atkinson, J.P., Cope, A.*, et al.* (2010). Complement regulator CD46 temporally regulates cytokine production by conventional and unconventional T cells. Nat Immunol *11*, 862-871.

Kemper, C., Leung, M., Stephensen, C.B., Pinkert, C.A., Liszewski, M.K., Cattaneo, R., and Atkinson, J.P. (2001). Membrane cofactor protein (MCP; CD46) expression in transgenic mice. Clin Exp Immunol *124*, 180-189.

Mrkic, B., Pavlovic, J., Rülicke, T., Volpe, P., Buchholz, C.J., Hourcade, D., Atkinson, J.P., Aguzzi, A., and Cattaneo, R. (1998). Measles virus spread and pathogenesis in genetically modified mice. J Virol *72*, 7420-7427.

Subramanian, A., Tamayo, P., Mootha, V.K., Mukherjee, S., Ebert, B.L., Gillette, M.A., Paulovich, A., Pomeroy, S.L., Golub, T.R., Lander, E.S.*, et al.* (2005). Gene set enrichment analysis: a knowledge-based approach for interpreting genome-wide expression profiles. Proc Natl Acad Sci U S A *102*, 15545-15550.

Wang, G., Liszewski, M.K., Chan, A.C., and Atkinson, J.P. (2000). Membrane cofactor protein (MCP; CD46): isoform-specific tyrosine phosphorylation. J Immunol *164*, 1839-1846.

Weyand, N.J., Lee, S.W., Higashi, D.L., Cawley, D., Yoshihara, P., and So, M. (2006). Monoclonal antibody detection of CD46 clustering beneath Neisseria gonorrhoeae microcolonies. Infect Immun *74*, 2428-2435.
