# Supplementary material for: Complement Regulates Nutrient Influx and Metabolic Reprogramming during Th1 Cell Responses
Source: Immunity. 2015 Jun 16;42(6):1033–47. doi: 10.1016/j.immuni.2015.05.024 (PMC4518498; doi:10.1016/j.immuni.2015.05.024)
Supplement: Document S2. Article plus Supplemental Information [file mmc6.pdf]

Immunity

Article

Complement Regulates Nutrient Influx and Metabolic Reprogramming during Th1 Cell Responses

Graphical Abstract

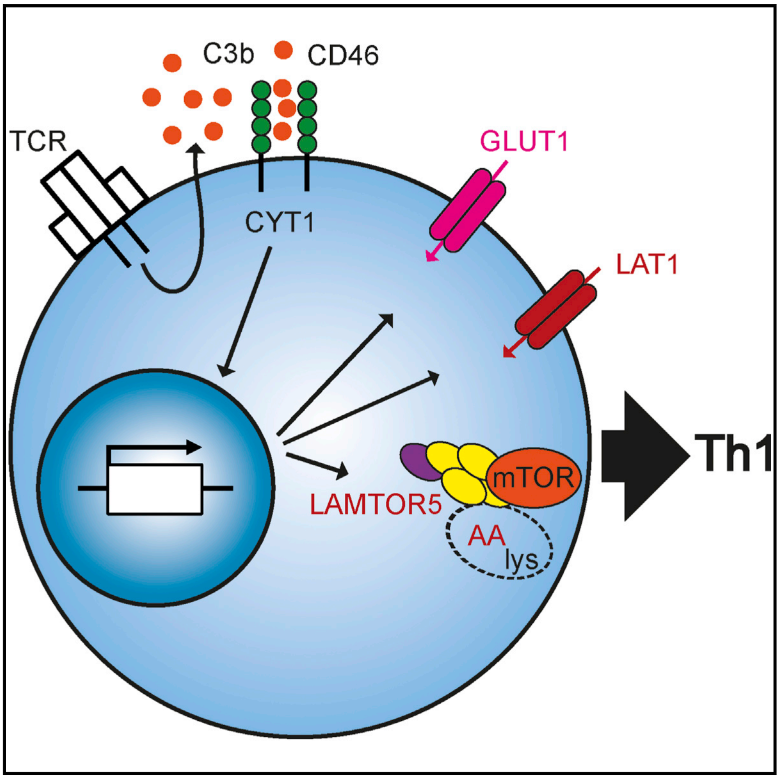

Highlights

- CD46 regulates GLUT1 and LAT1 and enhances glucose and AA uptake in T cells
- LAMTOR5 mediates Ragulator-Rag-mTORC1 assembly in activated T cells
- Complement drives glycolysis and oxidative phosphorylation critical to Th1 cell induction

Authors

Martin Kolev, Sarah Dimeloe, Gaelle Le Friec, ..., Paul Lavender, Christoph Hess, Claudia Kemper

Correspondence

chess@uhbs.ch (C.H.),
claudia.kemper@kcl.ac.uk (C.K.)

In Brief

The in vivo signals that drive metabolic reprograming of activated T cells remain poorly understood. Kemper and colleagues demonstrate that complement C3b enhances nutrient uptake and sensing in human T cells, enabling increased glycolysis and respiration required for Th1 responses.

Accession Numbers

GSE69090

Kolev et al., 2015, Immunity *42*, 1033–1047
June 16, 2015 ©2015 The Authors
http://dx.doi.org/10.1016/j.immuni.2015.05.024

Immunity
Article
Cell Press

Complement Regulates Nutrient Influx and Metabolic Reprogramming during Th1 Cell Responses

Martin Kolev,[1,8] Sarah Dimeloe,[2,8] Gaelle Le Friec,[1,8] Alexander Navarini,[3] Giuseppina Arbore,[1] Giovanni A. Povoleri,[1,4] Marco Fischer,[2] Réka Belle,[2] Jordan Loeliger,[2] Leyla Develioglu,[2] Glenn R. Bantug,[2] Julie Watson,[5] Lionel Couzi,[6] Behdad Afzali,[1,4,7] Paul Lavender,[5] Christoph Hess,[2,9,*] and Claudia Kemper[1,9,*]
[1]Division of Transplant Immunology and Mucosal Biology, MRC Centre for Transplantation, King's College London, Guy's Hospital, Great Maze Pond, London SE1 9RT, UK
[2]Department of Biomedicine, Immunobiology, University of Basel, 20 Hebelstrasse, 4031 Basel, Switzerland
[3]Department of Dermatology, University Hospital Zurich, 31 Gloriastrasse, 8091 Zürich, Switzerland
[4]Biomedical Research Centre, King's Health Partners, Guy's Hospital, Great Maze Pond, London SE1 9RT, UK
[5]MRC and Asthma UK Centre in Allergic Mechanisms of Asthma, King's College London, Guy's Hospital, Great Maze Pond, London SE1 9RT, UK
[6]Nephrology Transplantation, CHU Bordeaux, Hospital Pellegrin, CNRS UMR 1564, 146 rue Leo Saignat, 33076 Bordeaux, France
[7]Molecular Immunology and Inflammation Branch, National Institute of Arthritis and Musculoskeletal and Skin Diseases, National Institutes of Health, 10 Center Drive, Bethesda, MD 20892, USA
[8]Co-first author
[9]Co-senior author
*Correspondence: chess@uhbs.ch (C.H.), claudia.kemper@kcl.ac.uk (C.K.)
http://dx.doi.org/10.1016/j.immuni.2015.05.024
This is an open access article under the CC BY license (http://creativecommons.org/licenses/by/4.0/).

SUMMARY

Expansion and acquisition of Th1 cell effector function requires metabolic reprogramming; however, the signals instructing these adaptations remain poorly defined. Here we found that in activated human T cells, autocrine stimulation of the complement receptor CD46, and specifically its intracellular domain CYT-1, was required for induction of the amino acid (AA) transporter LAT1 and enhanced expression of the glucose transporter GLUT1. Furthermore, CD46 activation simultaneously drove expression of LAMTOR5, which mediated assembly of the AA-sensing Ragulator-Rag-mTORC1 complex and increased glycolysis and oxidative phosphorylation (OXPHOS), required for cytokine production. T cells from CD46-deficient patients, characterized by defective Th1 cell induction, failed to upregulate the molecular components of this metabolic program as well as glycolysis and OXPHOS, but IFN-γ production could be reinstated by retrovirus-mediated CD46-CYT-1 expression. These data establish a critical link between the complement system and immunometabolic adaptations driving human CD4$^+$ T cell effector function.

INTRODUCTION

Naive T cells are metabolically quiescent, primarily depending on oxidative phosphorylation (OXPHOS) for homeostatic adenosine triphosphate (ATP) generation (Gubser et al., 2013; Pearce et al., 2013; Rathmell, 2012; van der Windt et al., 2012, 2013). Ligation of the T cell receptor (TCR) and costimulatory molecules initiates significant changes in nutrient uptake and usage of metabolic pathways, jointly supporting bioenergetic and non-bioenergetic requirements of activated T cells (Gerriets and Rathmell, 2012; Jacobs et al., 2008; Pearce et al., 2013; Wang et al., 2011). Enhanced cellular uptake of amino acids (AA) is mediated by increased expression of several system L amino-acid transporters—particularly SLC7A5 (which together with SLC3A2 forms the neutral AA transporter LAT1). *SLC7A5*-deficient T cells, or T cells with compromised SLC3A2 expression, demonstrate broad metabolic defects and fail to proliferate and acquire effector functions (Sinclair et al., 2013; Wang et al., 2011). Likewise, upregulation of the glucose transporter GLUT1 (SLC2A1) and increased glycolytic flux (aerobic glycolysis) are needed for growth, expansion, and effector functionality (Macintyre et al., 2014) as is upregulation of OXPHOS, which supports T cell proliferation, interleukin (IL)-2 production, and enhanced glycolysis (Sena et al., 2013). Thus, activation of T cells is highly dependent on increasing AA-uptake, glycolytic flux, and OXPHOS.

The metabolic-checkpoint kinase mechanistic target of rapamycin (mTOR) senses and integrates environmental signals to regulate metabolic activity in cells. Activation of mTOR triggers glycolysis, OXPHOS, and lipid synthesis (Cunningham et al., 2007; Düvel et al., 2010; Shi et al., 2011) and mTOR-deficient CD4$^+$ T cells are unable to adjust metabolically and differentiate into effector cells (Delgoffe et al., 2009; Zheng et al., 2007). mTOR signals via two distinct complexes, mTOR complex 1 (mTORC1) and mTOR complex 2 (mTORC2), with mTORC1 activity inducing the enzymes needed for glycolysis and being specifically required for normal Th1 and Th17 cell induction (Pollizzi and Powell, 2014). Cytokines, availability of oxygen, and cellular energy levels all impact mTORC1 activity via the tuberous sclerosis 1 (TSC1)-TSC2 axis (Bar-Peled and Sabatini, 2014). By contrast, sensing sufficiency of AA by mTORC1 occurs via the RAS-related GTP-binding protein (Rag) family of small GTPases

CrossMark

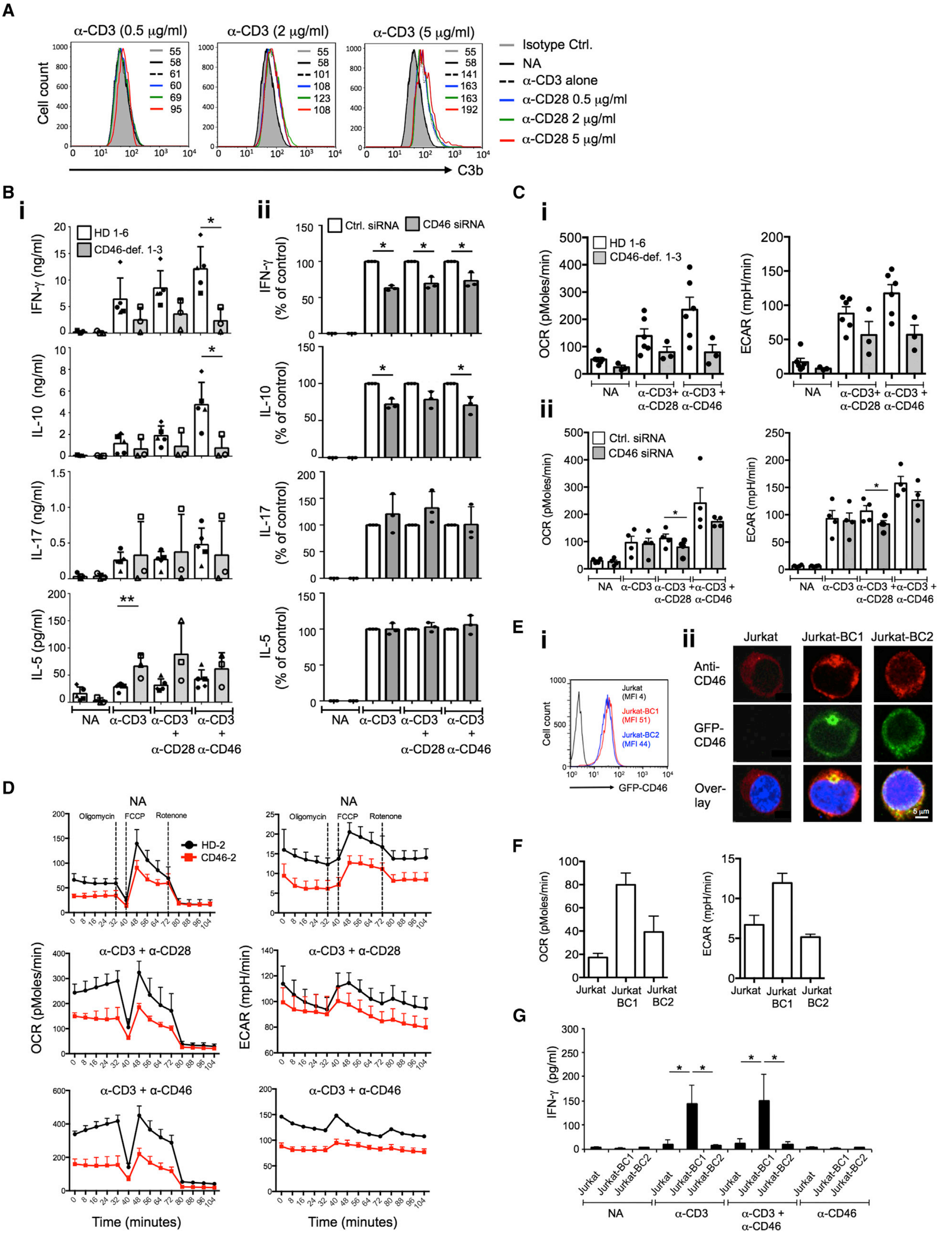

(legend on next page)

(Bar-Peled et al., 2012; Groenewoud and Zwartkruis, 2013; Long et al., 2005; Sancak et al., 2010). Specifically, in the presence of AA within the lysosomal lumen, the Rag A-B heterodimer becomes GTP-loaded and induces mTORC1 translocation to lysosomes, bringing it into close proximity of its activator Rheb (Groenewoud and Zwartkruis, 2013; Long et al., 2005; Sancak et al., 2010). The Rags are bound to lysosomes via the pentameric Ragulator complex, which consists of the "late endosomal or lysosomal adaptor and MAPK and mTOR activator" (LAMTOR 1–5) proteins (Sancak et al., 2010), and this LAMTOR-Rag-Rheb-mTORC1 complex then permits metabolic activation.

While the importance of metabolism in enabling T cell activation and differentiation is established, the in vivo pathways directing those events remain poorly defined. This is particularly true with regard to our understanding of metabolic reprogramming in human T cells. CD46, initially discovered as a complement regulator that binds and inactivates C3b and C4b (Liszewski et al., 1991), is also a key costimulatory molecule on human $CD4^+$ T cells (Astier et al., 2000; Cardone et al., 2010; Kemper et al., 2003; Le Friec et al., 2012). Importantly, CD46 is not expressed on hematopoietic cells in rodents and has no known functional homolog (Cope et al., 2011). In humans, CD46 is ubiquitously expressed in four distinct isoforms, with one of two alternatively spliced cytoplasmic tails, termed CYT-1 and CYT-2 (Liszewski and Atkinson, 1996), each mediating distinct signaling events (Cope et al., 2011). CD46-transduced signals are critically required for the induction of IFN-γ in human $CD4^+$ T cells, with indications that CYT-1 is driving Th1 cell polarization (Le Friec et al., 2012; Ni Choileain et al., 2011). Together with IL-2, CD46 also mediates IL-10 coexpression in expanded Th1 cells, and via this the switch toward a (self)regulatory contraction phase (Cardone et al., 2010). Unexpectedly, CD46-mediated activation of T cells is independent of systemic C3, but driven in an autocrine manner by the C3 activation fragment C3b generated by the T cell itself upon TCR activation (Cardone et al., 2010; Liszewski et al., 2013). Thus, lack of autocrine CD46 activation, such as in CD46- and C3-deficient patients, results in subnormal Th1 cell responses (Le Friec et al., 2012), whereas uncontrolled autocrine C3 activation and dysregulated CD46 engagement contribute to hyperactive Th1 cell responses in autoimmune pathologies (Astier et al., 2006; Cardone et al., 2010; Liszewski et al., 2013).

In this report we postulate a link between complement and key metabolic events regulating the human Th1 cell response. We characterize the dominant role of CD46 over CD28 in regulating GLUT1, LAT1, and nutrient uptake, define LAMTOR5 as part of the AA sensing machinery in human $CD4^+$ T cells, and develop a model interlinking metabolic reprogramming with CD46-mediated Th1 cell activation and contraction.

RESULTS

Autocrine CD46 Activation Is Required for Normal Glycolysis and OXPHOS

Autocrine CD46 activation by TCR-driven generation of C3b is an integral and non-redundant part of human Th1 cell induction and contraction (Liszewski et al., 2013). Activation of T cells with increasing amounts of monoclonal anti-CD3 and/or anti-CD28 mAbs was associated with larger surface deposition of C3b (Figure 1A), as well as IFN-γ production and IL-10 switching (Figure S1A). This suggested that modulation of Th1 cell cytokine production by varying TCR and costimulatory signal strength (Viola and Lanzavecchia, 1996) is impacted by CD46-mediated signaling. IFN-γ production by murine $CD4^+$ T cells is accompanied by specific metabolic changes (Chang et al., 2013), which led us to interrogate whether CD46 regulates human Th1 cell responses via modulation of key metabolic pathways. We used T cells from three patients with absent or severely reduced CD46 expression (CD46-1, CD46-2, and CD46-3 [Couzi et al., 2008; Fremeaux-Bacchi et al., 2006; and Figure S1B, legend]), T cells from healthy donors (HDs) in which CD46 protein expression was reduced by siRNA technique, and Jurkat T cell lines overexpressing specific CD46 isoforms. Whole-exome sequencing of DNA samples from patients CD46-2 (sibling of CD46-1) and CD46-3 confirmed the expected mutations in *CD46* but did not identify additional mutations in candidate genes mediating T cell function or genes known to cause monogenic immune defects (Table S1 and S2). While expression of CD3 and CD28 on T cells from all three patients was within normal range (Figure S1B), their $CD4^+$ T cells demonstrated impaired acquisition of Th1 cell effector function in response to TCR ligation and costimulation via either CD46 or CD28 (Cardone et al., 2010; Le Friec et al., 2012) (Figure 1Bi). The phenotype of T cells from HDs treated with CD46-specific siRNA (Figure S1Ci) was comparable, with a specific reduction in IFN-γ and IL-10, but normal IL-5 production (Figure 1Bii), and reduced upregulation of CD25 (Ni Choileain et al., 2011), but unaltered expression of CD69 (Figure S1Cii) (Le Friec et al., 2012). Only T cells from patient CD46-3, which lacked CD46 expression entirely (Figure S1B, legend), were unable to produce IL-17.

As upregulation of mitochondrial respiration (OXPHOS) and aerobic glycolysis is central to T cell effector function, we assessed the metabolic profile (mitochondrial respiration (oxygen

Figure 1. Autocrine CD46-CYT-1 Activation Drives Glycolysis and Oxidative Phosphorylation in $CD4^+$ T Cells

(A) TCR and CD28-induced Th1 cell cytokine production correlates with CD46 ligand C3b generation as assessed 1 hr post activation.

(B) Cytokines produced by (Bi) $CD4^+$ T cells from age- and sex-matched healthy donors (HD1 to HD6) and patients CD46-1 (open circle), CD46-2 (open square), and CD46-3 (open triangle) or by (Bii) T cells from HDs treated with CD46 siRNA (n = 3 with duplicate samples [mean]).

(C) Basal glycolysis (ECAR) and oxidative phosphorylation (OXPHOS, OCR) rates in resting and activated $CD4^+$ T cells (Ci) from CD46-deficient patients (n = 3) and HDs (n = 6) or from (Cii) HD T cells after CD46-specific siRNA treatment.

(D) Respiratory capacity and glycolysis in T cells from a HD and from patient CD46-2, basally and following mitochondrial perturbation.

(E) CD46 expression in Jurkat T cells transfected with GFP-tagged CD46-CYT1 (Jurkat-BC1) or CD46-CYT2 (Jurkat-BC2) isoforms. (Ei) FACS-assessed surface expression of GFP-tagged CD46 and (Eii) endogenous (red) and recombinantly overexpressed CD46 (green) by confocal microscopy (n = 3).

(F) Basal glycolysis and OXPHOS levels in Jurkat, Jurkat-BC1, and Jurkat-BC2 cells (n = 3).

(G) CD46-BC1 isoform overexpression restores IFN-γ upon TCR activation in Jurkat cells (n = 3, IFN-γ measured 3 days post activation).

Magnification (Eii) × 100. *$p < 0.05$; **$p < 0.01$. Error bars represent mean ± SEM. See also Figure S1.

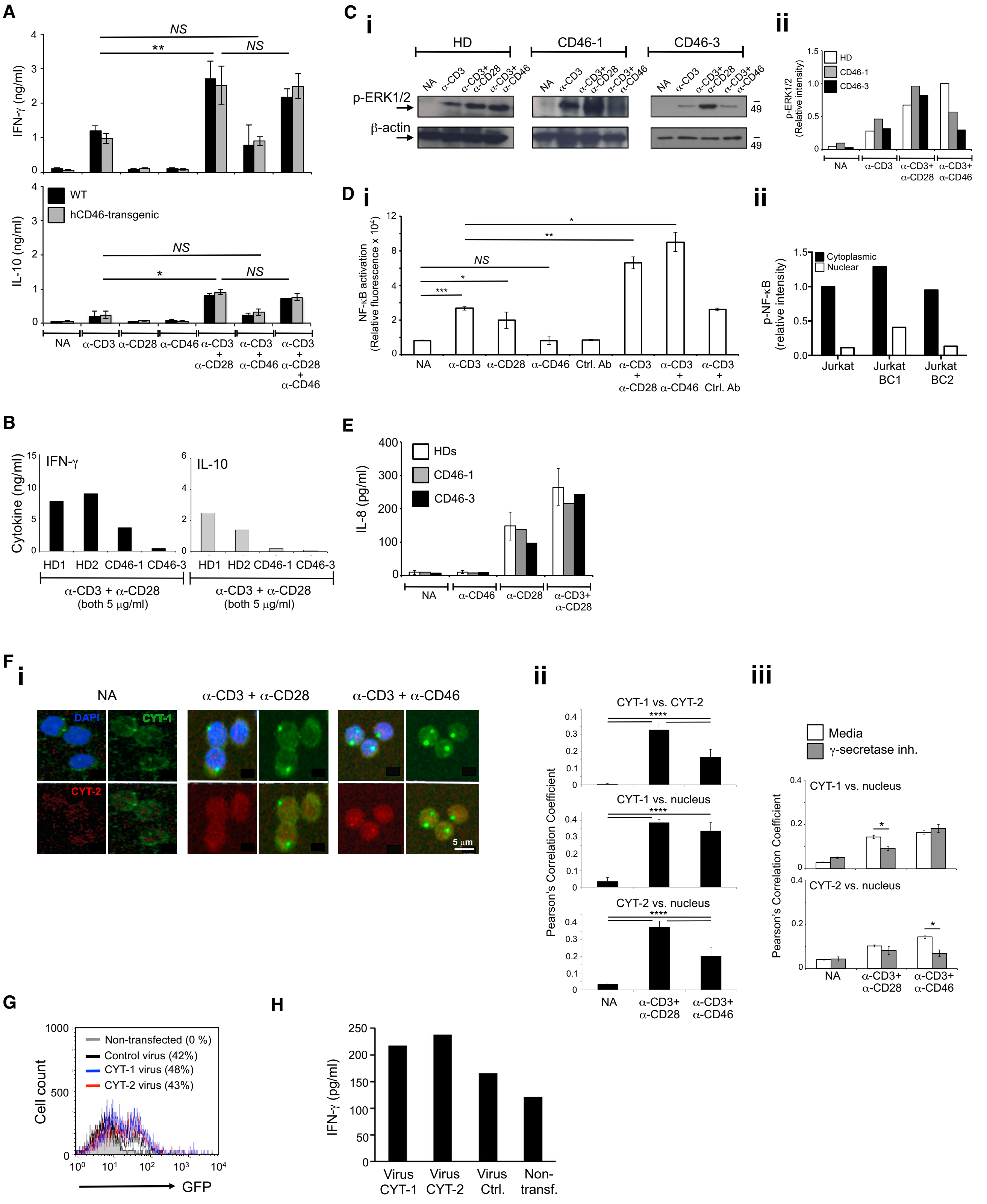

Figure 2. CD46 Costimulation Is Human Specific and Operates Differently from CD28

(A) CD4^{+} T cells from hCD46-transgenic or wild-type (WT) mice were stimulated with anti-mouse CD3 and CD28 and anti-human CD46 and cytokines measured 72 hr post activation (n = 3).

(legend continued on next page)

consumption rate – OCR)), and aerobic glycolysis (extracellular acidification rate – ECAR) of T cells from the CD46-deficient patients and six HDs (Figure 1Ci). Compared to controls, non-activated and CD3+CD28-activated CD4$^+$ T cells from patients showed a trend toward reduced respiration and glycolysis (Figure 1Ci), and the increase in OCR and ECAR levels upon CD3+CD46 activation—which was present in all HDs—was absent in CD46-deficient individuals (Figure 1Ci). Notably, by 36 hr of activation, similar OCR and ECAR levels were observed in CD3 and CD3+CD28-activated HD CD4$^+$ T cells (Figures S1Ei and S1Eii). A comparable reduction in OCR and glycolysis was observed using HD-derived T cells in which CD46 expression was knocked down (Figure 1Cii), with the reduction of respective CD46 expression corresponding with reduction in OCR and ECAR (not shown). We also observed a parallel reduction in ATP-coupled and maximal respiration, as well as maximal glycolytic rate of activated cells, when comparing CD46-deficient CD4$^+$ T cells to those from HDs (Figure 1D, patient CD46-2; Figure S1F, summary for all three patients; and Figure S1G for HD T cells after CD46-specific siRNA treatment).

CD46 is expressed in distinct isoforms with two potential intracellular domains, CYT-1 or CYT-2 (Figure S1H), and CYT-1 is required for IFN-γ production in CD4$^+$ T cells (Le Friec et al., 2012). To assess whether CYT-1 also mediated the metabolic changes observed during T cell activation, we used non-manipulated Jurkat T cells (which mostly express CD46 CYT-2-bearing isoforms and do not produce IFN-γ upon activation), or Jurkat cells stably overexpressing either a GFP-tagged CD46 isoform bearing CYT-1 (Jurkat-BC1) or CYT-2 (Jurkat-BC2) (Le Friec et al., 2012) at equivalent levels (Figure 1Ei), and with normal cellular distribution (Figure 1Eii). Jurkat-BC1 cells indeed had increased basal OCR and ECAR compared to non-transfected or Jurkat-BC2 cells (Figure 1F) and, importantly, this increase was accompanied by the induction of IFN-γ production upon activation (Figure 1G).

Thus, autocrine C3b-driven activation of CD46 induced the metabolic changes in human CD4$^+$ T cells that drive Th1 cell induction.

CD46 Costimulation Is Non-redundant and Operates Differently from CD28

Mice lack CD46 expression on lymphocytes (Cope et al., 2011), and CD4$^+$ T cells isolated from transgenic mice expressing human CD46 in a human-like pattern (Kemper et al., 2001) did not increase IFN-γ or IL-10 production upon activation (Figure 2A). Thus, mouse T cells are not equipped with the machinery for CD46-induced signal transduction, and CD28 is the critical costimulatory molecule in this species.

Defective Th1 cell induction in CD46-deficient patients could not be overcome by increasing TCR and CD28 signal strength (Figure 2B), while TCR- and CD28-driven phosphorylation of extracellular signal-regulated kinase 1/2 (ERK1/2) was unaffected (Figures 2Ci and 2Cii). Furthermore, although CD46 stimulation, and specifically CYT-1, potentiated TCR-induced NF-κB activation (Figures 2Di and Dii), it did not induce NK-κB activation on its own (Figure 2Di) and failed to induce IL-8 secretion (Figure 2E)—two events that are driven by TCR-independent CD28 signals (Marinari et al., 2004) and that similarly occurred in T cells from HDs and CD46-deficient patients (Figure 2E). These data demonstrated that TCR- and CD28-mediated signals did function properly in CD46-deficient patients but that these signals were not sufficient for normal Th1 cell induction.

Upon CD46 activation, CYT-1 and CYT-2 are cleaved and released intracellularly by γ-secretase (Ni Choileain et al., 2011), and inhibition of γ-secretase activity prevents CD46-driven Th1 cell induction (Figure S2A) (Le Friec et al., 2012). Because both CYT-1 and CYT-2 of CD46 contain nuclear targeting signals (Figure 1H) we assessed whether the tails translocate into the nucleus upon activation. Using confocal microscopy (Figures 2Fi and 2Fii) and Image Stream (Figure S2B), we indeed observed nuclear translocation of both CYT-1 and CYT-2 in activated T cells. CYT-1 translocation was significantly inhibited by γ-secretase inhibitor treatment in CD3+CD28-activated T cells, while this treatment prevented CYT-2 nuclear translocation in CD3+CD46-activated cells (Figure 2Fiii), suggesting that the coordinated CD46 cytoplasmic domain processing and/or nuclear translocation may be impacted by both CD28 and CD46 stimulation. To mimic CD46 cytoplasmic domain release in CD46-deficient T cells, we transfected T cells from patient CD46-3 with retroviruses expressing either CYT-1 or CYT-2 only (Figure 2G), which induced substantially increased IFN-γ production (but not IL-4 or IL-5 production, data not shown) in these cells (Figure 2H). The unexpected observation that CYT-2 transfection also rescued IFN-γ production in CD46-deficient T cells was likely due to the fact that cleaved CYT-2 positively regulated CYT-1 expression, as demonstrated by assessment of both CYT-1 and CYT-2 protein expression of parental Jurkat T cells after transfection with each virus alone (Figure S2C).

Together these data demonstrated that CD46 costimulation in human CD4$^+$ T cells was non-redundant and required nuclear translocation of its cytoplasmic tails to the nucleus.

(B) Increased TCR and CD28 activation cannot rescue defective Th1 cell induction in CD46-deficient T cells. Cells from HD1 and HD2 and patients CD46-1 and CD46-3 were activated as indicated and cytokines measured at 36 hr.

(C) TCR and CD28-driven ERK1/2 phosphorylation occurs optimally in CD46-deficient T cells as assessed by (Ci) western blot and (Cii) densitometric analyses 30 min post activation.

(D) CD46 induced canonical NF-κB activation utilizing (Di) T cells transfected with a NF-kB luciferase reporter plasmid and NF-κB activation measured at 1 hr post activation and measuring (Dii) NF-κB activation in Jurkat, Jurkat-BC1, and Jurkat-BC2 cells (n = 4).

(E) CD28 induces normal IL-8 secretion in T cells from patients at 36 hr post activation.

(F) CD46 CYT-1 and CYT-2 translocate to the nucleus upon cleavage by γ-secretase as assessed by (Fi) confocal microscopy using CYT-1 and CYT-2-specific antibodies with analyses of colocalization events in (Fii) the absence or (Fiii) presence of γ-secretase inhibition (n = 3).

(G and H) Transfection of CD46 intracellular domains rescues IFN-γ production in T cells from patient CD46-3.

(G and H) Transfection efficiency (G) of T cells isolated from patient CD46-3 transfected with retroviruses expressing either CYT-1 or CYT-2 (or the GFP reporter gene) and (H) IFN-γ production by CD4$^+$ T cells from patient CD46-3 after retroviral transfection at 24 hr post CD3+CD28 activation.

*p < 0.05; **p < 0.01; ***; p < 0.005; ****p < 0.001; NS, statistically not significant. Error bars represent mean ± SEM. See also Figure S2.

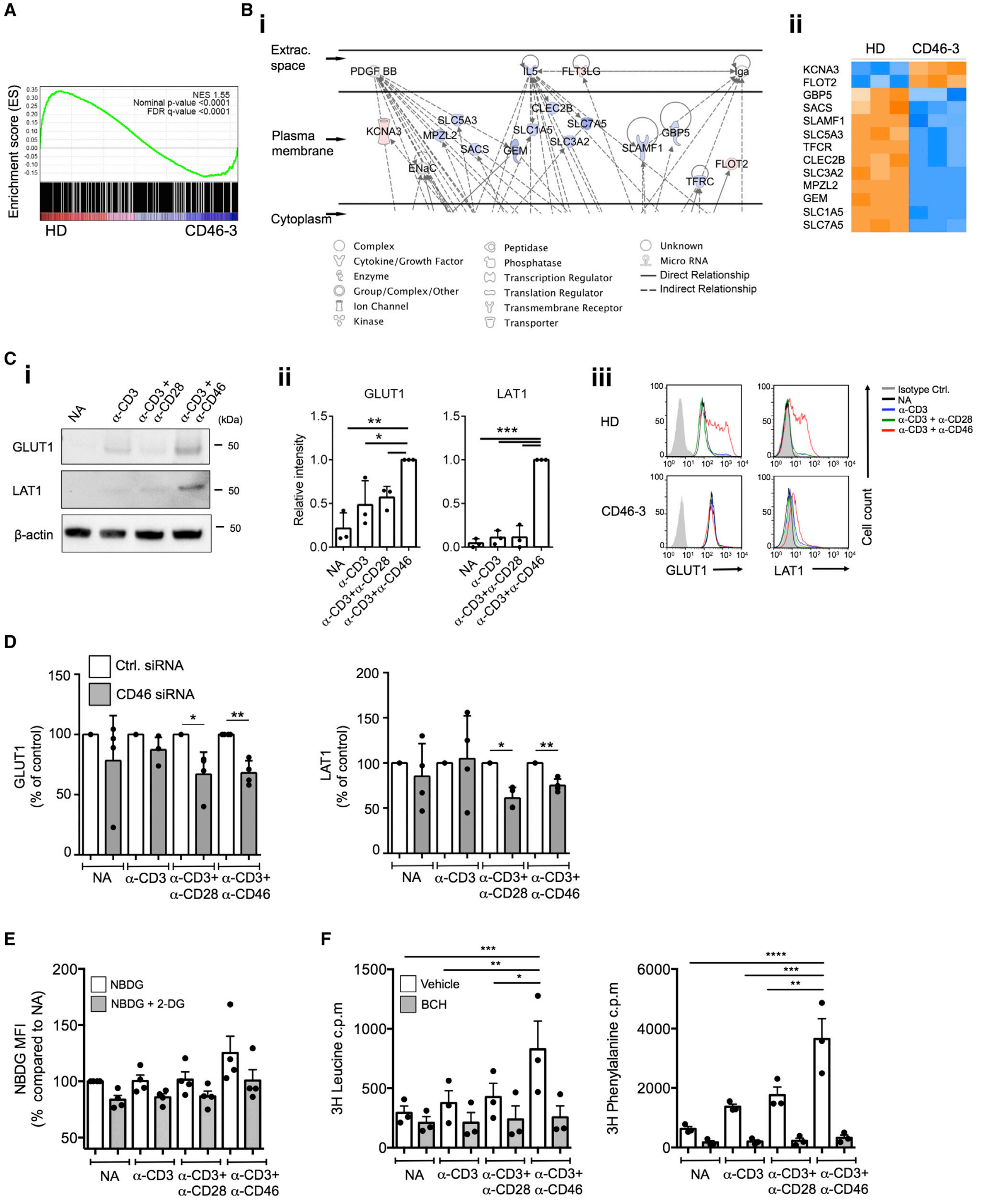

(legend on next page)

CD46 Induces Nutrient Influx via Glucose and Amino Acid Transporter Upregulation

To identify the CD46-induced molecular pathways driving the observed metabolic events, we performed gene expression arrays on mRNA isolated from CD3+CD46-activated T cells from patient CD46-3. Aligning with the functional T cell phenotype (severely reduced glycolysis and OXPHOS, Figure 1Ci), genes associated with regulation of metabolic processes were significantly enriched in the HD compared to the patient by gene set enrichment analysis (GSEA) (Subramanian et al., 2005) (Figure 3A). Similarly, Gene Ontology analysis of the 403 transcripts differentially expressed between CD3+CD46-activated T cells of the HD and patient CD46-3 (Table S3) revealed that almost 30% of these genes (118 genes, Table S4) were functionally involved in the regulation of metabolic processes (Figures S3Ai and S3Aii). Furthermore, Ingenuity Pathway Analysis suggested that T cells from patient CD46-3 lacked activation-induced expression of several key AA transporters. Specifically, of the top five gene networks modeled by the software, two were functionally involved in AA metabolism (Figures 3Bi and 3Bii and Figures S3Bi and S3Bii). Upregulation of the glucose transporter GLUT1 (Macintyre et al., 2014) and L-type AA transporter LAT1 (SLC7A5; reduced in T cells from patient CD46-3 [Figure 3Bii]) are required for successful CD4$^+$ T cell activation in mice (Sinclair et al., 2013) and humans (Hayashi et al., 2013). When assessing activated T cells 36 hr post stimulation for the expression of GLUT1 and LAT1, we found that CD3+CD46 activation induced strongest expression of these channels when compared to CD3 or CD3+CD28-activated cells (Figures 3Ci and 3Cii), while CD46 activation alone had no effect on GLUT1 or LAT1 expression (data not shown). Accordingly, T cells isolated from patient CD46-3, and T cells from HDs in which CD46 expression was decreased by siRNA treatment, had impaired GLUT1 and LAT1 upregulation (Figures 3Ciii and 3D). Whereas CD46 potentiated TCR-driven GLUT1 expression, LAT1 upregulation seemed to require CD46-driven signals, as only CD46 co-engagement induced significant LAT1 expression in T cells (Figure 3Cii). This aligns with the observation that CD3+CD46 activation moderately increased glucose uptake over CD3 and CD3+CD28-activated T cells (Figure 3E), but that LAT1-dependent uptake of the AAs Leucine and Phenylalanine at 36 hr post activation was significantly enhanced by CD46 costimulation (Figure 3F, and Figure S3C for a time course of GLUT1 and LAT1 expression and glucose and AA uptake). Consistent with the role of CD46 CYT-1 in driving glycolysis and OXPHOS, CYT-1, but not CYT-2, induced GLUT1 and LAT1 upregulation as demonstrated by the increased steady-state expression specifically in Jurkat-BC1 cells (Figures S3Di–S3Diii), and their concurrently increased basal uptake of glucose, Leucine, and Phenylalanine (Figures S3E and S3F).

These data demonstrated that CD46 CYT-1 costimulation during TCR activation was required for GLUT1 and LAT1 expression, upregulation, and subsequent glucose and AA uptake in human CD4$^+$ T cells.

CD46 Is Required for mTORC1 Activity

Since glucose and AA uptake induces mTORC1 activation (Bachar et al., 2009; Sancak et al., 2010), we assessed whether CD46 impacts mTORC1 activity by measuring the phosphorylation of the mTORC1 downstream target p70S6K (position T389) (Matheny and Adamo, 2009). At 1 hr post activation, CD3, CD3+CD28, and CD3+CD46 activation each induced significant increases in p70S6K phosphorylation when compared to resting cells. Notably, CD46 costimulation not only resulted in the highest levels of p-p70S6K at 1 hr post activation, but also sustained p70S6K phosphorylation consistently up to at least 36 hr post activation (Figures 4Ai and 4Aii). The observed CD46-mediated p70S6K phosphorylation was dependent on mTORC1, as the mTORC1 inhibitor Rapamycin abrogated CD46-mediated p70S6K phosphorylation (Figures 4Bi and 4Bii). Furthermore, mTORC1 activation supports upregulation of GLUT1 (Bhaskar et al., 2009) and LAT1 (Roos et al., 2009), and in accordance with these data, the addition of Rapamycin during CD3+CD46 stimulation reduced the expression of these nutrient transporters (Figure 4C). The dependence of CD4$^+$ T cells on CD46 costimulation for normal mTORC1 function was further underscored by the inability of T cells from patient CD46-3 to induce either mTOR or p70S6K phosphorylation at substantial levels under any activation condition tested (Figures 4D and S4), and by a significant reduction in mTOR and p70S6K phosphorylation in T cells from HDs treated with CD46-specific siRNA (not shown). In keeping with the fact that CD46 CYT-1 was the driver of nutrient influx, glycolysis, and OXPHOS in T cells, Jurkat-BC1 cells had higher mTOR and p70S6K phosphorylation levels compared to Jukat-BC2 cells or the parental Jurkat line (Figure 4E).

CD46 Activation Supports LAMTOR5-Driven Assembly of Ragulator-Rag-mTORC1

The nature of the Ragulator complex activating mTORC1 in human CD4$^+$ T cells is undefined. LAMTOR5 is a recently discovered member of the Ragulator complex (Bar-Peled et al., 2012) and, although LAMTOR5 has previously not been described in T cells, the corresponding mRNA was induced in gene arrays performed using non-activated and CD3+CD46-activated CD4$^+$ T cells from HDs (data not shown). Indeed, measurement of LAMTOR5 protein in purified healthy CD4$^+$ T cells established

Figure 3. CD46 Mediates Glucose and AA Channel Expression and Nutrient Influx in CD4$^+$ T Cells

(A and B) Gene expression array and Ingenuity Pathway Analysis (IAP) comparison of CD46-sufficient and -deficient CD4$^+$ T cells activated for 2 hr with anti-CD3+CD46 mAb with (A) gene set enrichment analysis (GSEA) and (B) extract of IPA output showing (Bi) membrane-associated genes involved in AA metabolism (full figure in Figure S2B) and (Bii) a heatmap of those genes.

(C) GLUT1 and LAT1 expression on T cells from HDs 36 hr post activation assessed by (Ci) western blotting with (Cii) the corresponding statistical analyses via densitometric measurement, and (Ciii) from patient CD46-3 measured by FACS (n = 3).

(D) CD46 silencing prevents normal GLUT1 and LAT1 expression (n = 4; 72 hr post activation).

(E and F) Glucose and AA uptake upon CD46 activation with (E) glucose uptake assessed with or without addition of competing unlabeled 2-DG and (F) AA uptake measured with or without addition of a LAT1 inhibitor (BCH) (n = 3).

*$p < 0.05$; **$p < 0.01$; ***$p < 0.005$; ****$p < 0.001$. Error bars represent mean ± SEM. See also Figure S3.

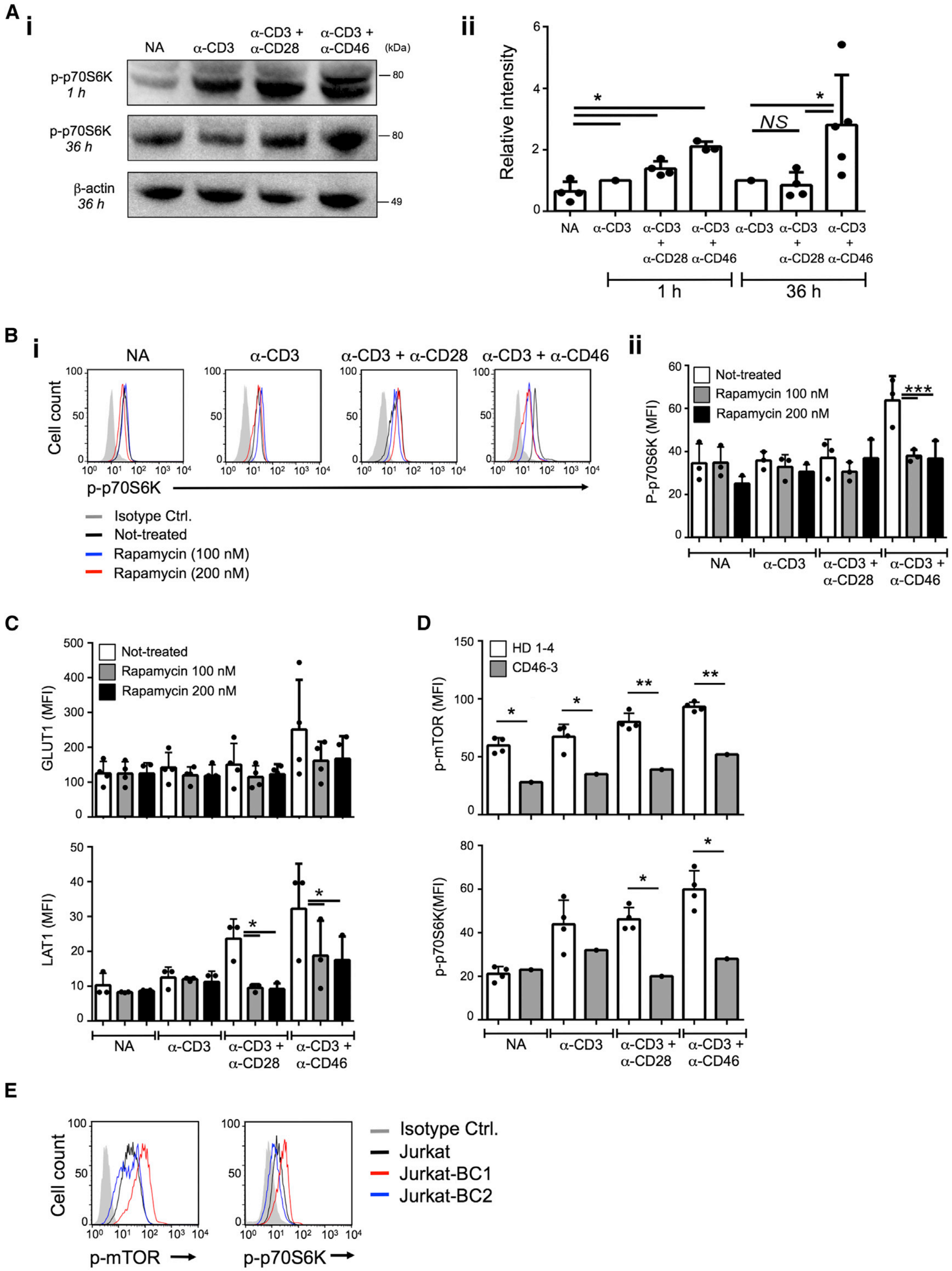

Figure 4. CD46 Regulates mTORC1 Activity in CD4+ T Cells

(A) CD46 activation sustains p70S6K phosphorylation as assessed by (Ai) western blotting with (Aii) the corresponding statistical analyses via densitometric measurement of band intensities (n = 4–5). (B) Effect of rapamycin on p70S6K phosphorylation (p-p70S6K) at 36 hr with (Bi) a representative FACS analysis of n = 3, and (Bii) depicting their statistical analysis. (C) Effect of Rapamycin on GLUT1 and LAT1 expression. T cells were activated as under (B) and expression of GLUT1 (upper panel) and LAT1 (lower panel) measured (n = 4).

(D) mTOR (p-mTOR) and p70S6K phosphorylation in T cells from HD1-4 and patient CD46-3 36 hr post activation.

(E) p-mTOR and p70S6K levels in Jurkat, Jurkat-BC1, and Jurkat-BC2 cells (n = 3).

*$p < 0.05$, **$p < 0.01$; ***$p < 0.005$. Error bars represent mean ± SEM. See also Figure S4.

that CD46 costimulation induced a significant increase in LAMTOR5 (Figures 5A and S5A for a time-course), while this increase was absent in T cells from patient CD46-3 (Figure 5B), and in T cells from HDs where CD46 expression was decreased by siRNA technique (Figure 5C). Confocal microscopy studies with analyses of protein colocalization coefficients demonstrated that TCR activation, specifically with CD46 costimulation, drove colocalization of LAMTOR5, the Ragulator complex partner GTPase RagC, and mTOR on lysosomes (LAMP1) (Figures 5D and 5E), with reduction of these events in T cells from patient CD46-3 (Figure 5D). Results obtained from CD46 isoform-transfected Jurkat cells confirmed that LAMTOR5 expression was increased by CYT-1 (Figures S5Bi and S5Bii), while the expression of RagC remained largely unaffected by CD46 signaling (Figure S5C).

The central role for LAMTOR5 in mTORC1 lysosomal translocation was further confirmed by the finding that knockdown of LAMTOR5 protein in T cells from HDs reduced colocalization for all assessed proteins of this complex (Figures 5F and 5G) and led to a significant decrease in LAT1 expression (by about 50%, Figure S5D) and p70S6K phosphorylation by about 75% in CD3+CD46-activated T cells (Figure S5E).

These results identified Ragulator LAMTOR5 as a critical mTORC1 assembly platform in human $CD4^+$ T cells.

CD46 Isoform Expression Correlates with Metabolic Changes in the Th1 Cell Life Cycle

CD46 costimulation is not only key for IFN-γ production in human Th1 cells but also for the induction of IL-10 coproduction and the switch toward their (self)regulating and contracting "life cycle" phase (Cardone et al., 2010). Thus, following T cell activation via CD3+CD46 four populations of cells are sequentially induced with distinct cytokine profiles: IFN-γ^+, IFN-γ^+IL-10^+, IL-10^+, and IFN-γ^-IL-10^-. Accordingly, T cells from CD46-deficient patients have impaired production of both IFN-γ and IL-10 but have no defect in Th2 responses (IL-4 and IL-5) (Figure 1B and Le Friec et al., 2012). In line with the requirement of glucose and AA uptake for early IFN-γ production (Pearce et al., 2013; Sinclair et al., 2013), when we activated human $CD4^+$ T cells from healthy donors via CD3, CD3+CD28, or CD3+CD46, we found that the presence of 2-DG (which inhibits glycolysis) or the LAT1 inhibitor BCH reduced production of IFN-γ and IL-10 under all activation conditions (Figures 6A and 6B, upper and lower panels, respectively). Also, inhibition of mTORC1 with Rapamycin and LAMTOR5 protein knockdown through mRNA silencing reduced both IFN-γ and IL-10 production (Figures 6C and 6D)—but did not significantly affect IL-4 and IL-17 secretion (data not shown).

Resting $CD4^+$ T cells expressed all four isoforms of CD46: BC1, C1, BC2, and C2 (Figure S1H). Upon activation, however, the isoform expression pattern changed, with an increase in CYT-1-bearing forms (i.e., BC1 and C1) (Liszewski et al., 2013) (Figure 7Ai). Furthermore, several studies have implicated CYT-1 of CD46 as a Th1 cell "driver" (Le Friec et al., 2012; Ni Choileain et al., 2011). Because this parallels our observation that specifically CYT-1 of CD46 was required for mTORC1 activation, we hypothesized that CYT-1 versus CYT-2 expression is different in IFN-γ^+, IFN-γ^+IL-10^+, IL-10^+, and IFN-γ^-IL-10^- subpopulations and that expression changes with their progression through the Th1 cell life cycle. To address this, we sorted the Th1 cell subpopulations resulting from CD3+CD46 activation and assessed respective CD46 mRNA CYT-1 versus CYT-2 expression patterns. CYT-1 expression increased over CYT-2 expression in IFN-γ^+ and IFN-γ^+IL-10^+ populations, while IL-10^+ T cells switched back toward a CYT-2 predominant profile (Figures 7Ai and 7Aii). Moreover, the expression of GLUT1, LAT1, and LAMTOR5 and the phosphorylation of mTOR and p70S6K, as well as OXPHOS and glycolysis levels, all paralleled the expression kinetics of CYT-1—with all being also increased in IFN-γ^+ and IFN-γ^+IL-10^+ subsets, while returning to basal levels in IL-10^+ and IFN-γ^-IL-10^- cells (Figures 7B–7D).

These results demonstrated that the temporal changes in CD46 isoform expression induced upon T cell activation mediated the metabolic events specific to the induction, effector function, and contraction phases of Th1 cells and, thus, demarcated the human Th1 cell life cycle phases (Figures S6A and S6B).

DISCUSSION

Glucose metabolism, OXPHOS, AA influx, and differential activation of the metabolic checkpoint kinase mTOR each play important roles in enabling successful T cell immunity (Jones and Thompson, 2007; MacIver et al., 2013; Pollizzi and Powell, 2014). However, in humans the receptor(s) triggering, and the molecular events mediating, distinct immune-metabolic activities in vivo are not well defined. Here we provide a molecular framework that integrates the complement receptor CD46 with metabolic reprogramming required for human Th1 cell induction, and we demonstrate dysregulation of this key metabolic program in patients with reduced CD46 expression. We suggest a model in which TCR activation, which induces the local generation of CD46 ligand C3b (Liszewski et al., 2013), increases expression of CD46 isoforms bearing CYT-1. CD46 CYT-1-driven signals then mediate upregulation of GLUT1 and, more importantly, LAT1, allowing for increased glucose and AA influx into the cell. Increased expression of LAMTOR5 and assembly of the lysosome-based machinery simultaneously enables AA sensing via mTORC1. mTORC1 activation and downstream events, including further induction of glycolysis and OXPHOS, then support Th1 cell maturation and IFN-γ production. During Th1 cell contraction and induction of IL-10 coexpression, CD46 isoform expression of $CD4^+$ T cells reverts to a CYT-2 predominant pattern, accompanied by reduced expression of GLUT1 and LAT1, and downregulation of OXPHOS and glycolysis. This model aligns with the facts that the *SLC2A1* gene is hypermethylated in patient CD46-3; that high GLUT1 expression is selectively required for effector but not regulatory T (Treg) cell responses (Macintyre et al., 2014); that Treg cells do not upregulate CD46 CYT-1 upon activation, and that Treg cell numbers and functions are normal in CD46-deficient patients (Liszewski et al., 2013).

A complement receptor or regulator serving as the murine "CD46 homolog" with regard to Th1 cell regulation has not been identified, and published work indicates that CD28 drives glycolysis and OXPHOS as required for effector T cells function in mice. CD28 can also regulate GLUT1 expression and glycolysis (Frauwirth et al., 2002; Jacobs et al., 2008) and LAT1 induction (Hayashi et al., 2013) in activated human $CD4^+$ T cells. However, because costimulation via CD28 potentiates TCR-induced autocrine generation of C3b, and CD28 signals are not sufficient

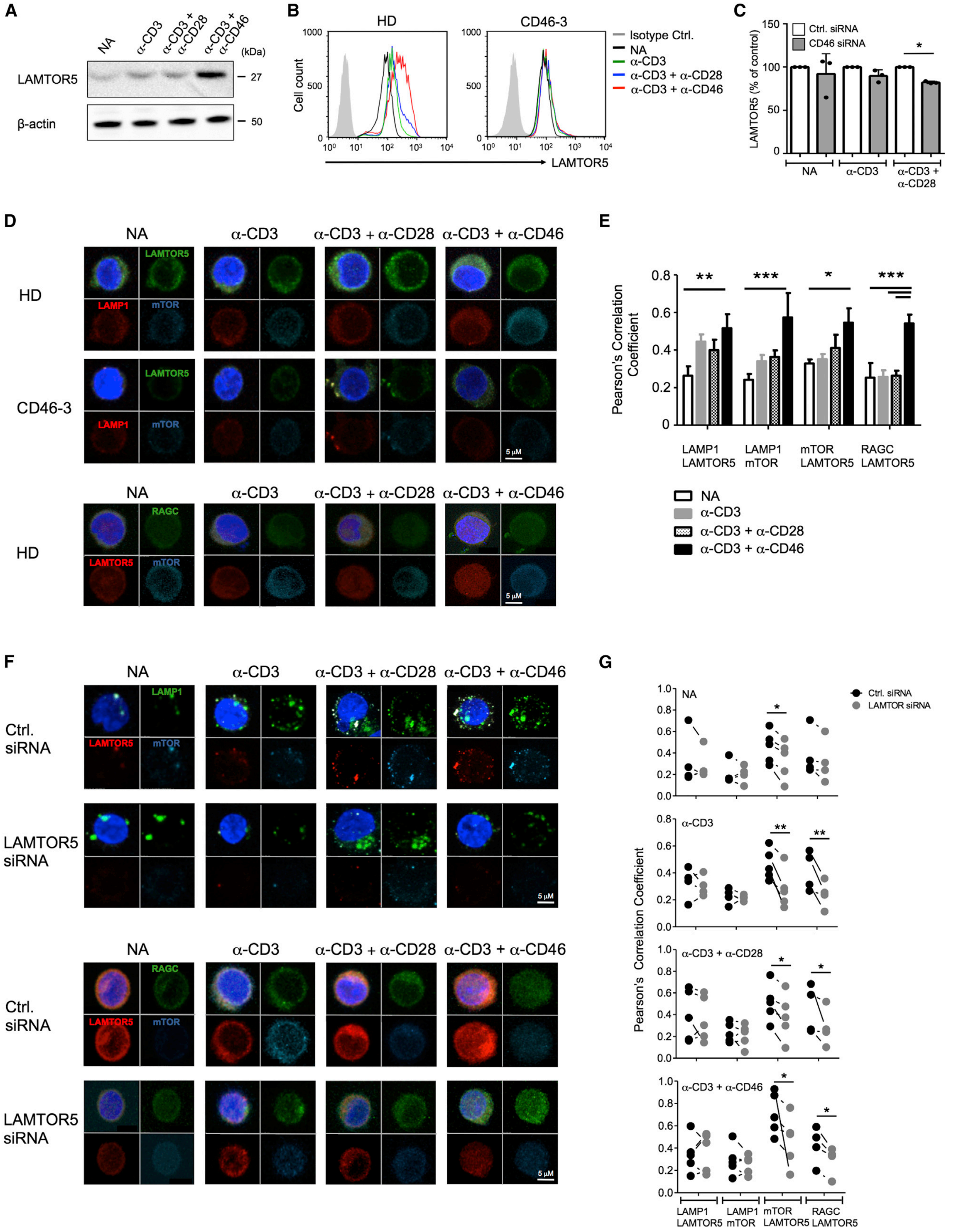

(legend on next page)

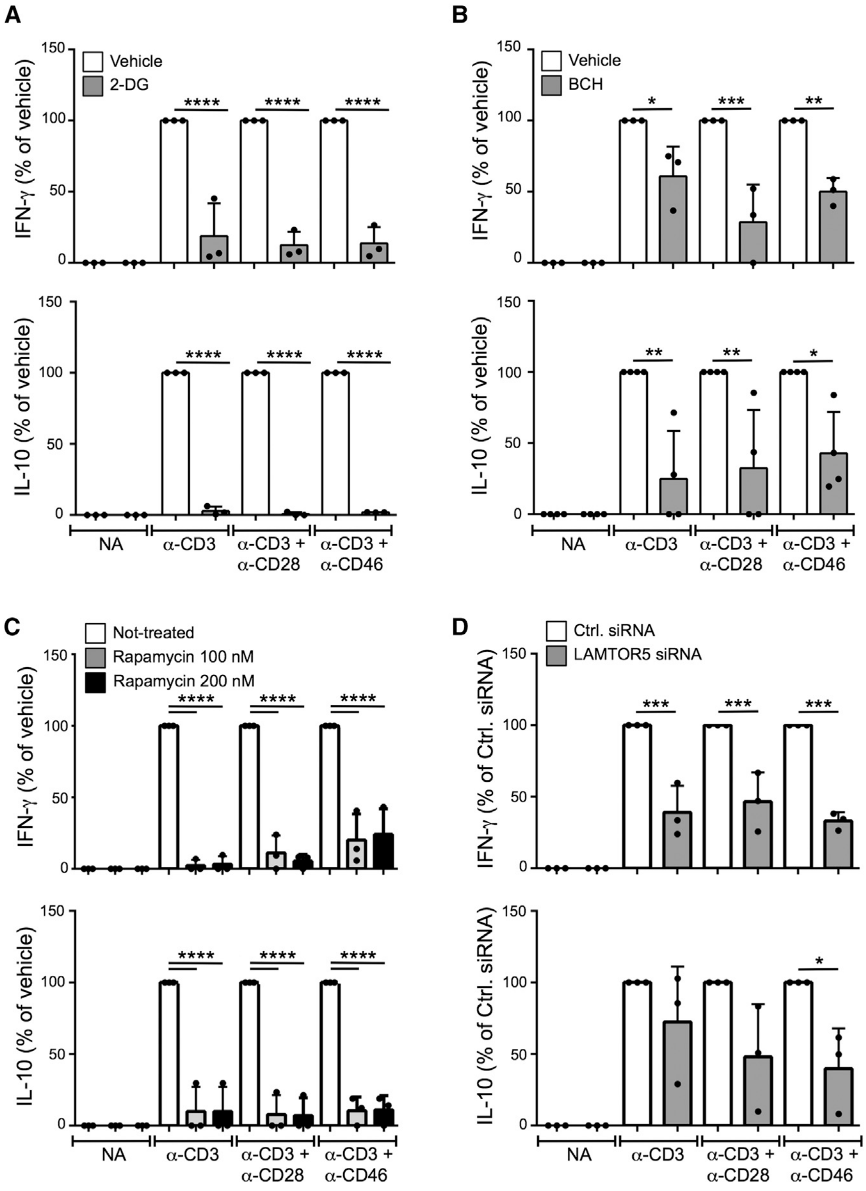

Figure 6. CD46-Driven Glucose and Amino Acid Influx and mTORC1-Activity Are Critical to Human Th1 Cell Induction

(A and B) Th1 cell induction (upper panels) and IL-10 switching (lower panels) assessed at 36 hr post activation in the presence of 2-deoxyglucose (2-DG) and BCH.

(C) Effect of mTORC1 inhibition on Th1 cell induction at 36 hr post activation.

(D) Impact of LAMTOR5-silencing on Th1 cell induction. $CD4^+$ T cells transfected with siRNAs as shown were activated as depicted for 36 hr and analyzed for IFN-γ and IL-10 production.

Data shown in (A)–(D) are n = 3. NA, non-activated. $^*p < 0.05$; $^{**}p < 0.01$; $^{***}p < 0.005$; $^{****}p < 0.001$. Error bars represent mean ± SEM.

to induce Th1 cell responses in CD46-deficient T cells, increased autocrine CD46 engagement plays a key role in the CD28-driven nutrient uptake in CD46-sufficient cells. Nonetheless, a cooperation between CD28- and CD46-intrinsic molecular events jointly supporting optimal Th1 cell induction remains an important possibility, perhaps with CD28 as an upstream regulator of the autocrine "C3-CD46" axis in T cells.

$CD4^+$ T cell subsets have distinct metabolic requirements. Effector T cells demand high levels of glycolysis, whereas Treg cells are more dependent on OXPHOS, and mTORC1 activity is required for Th1 and Th17 cell responses, whereas mTORC2 drives Th2 cell function (Delgoffe et al., 2009; Michalek et al., 2011; Shi et al., 2011). Congruent with this, decreased expression of CD46 impacted proportionally on mTORC1 activity and Th1 cell induction, whereas Th2 cell responses remained unaffected. Interestingly, only complete absence of CD46 led to failure of Th17 cell induction, suggesting metabolic threshold differences between induction of Th1 and Th17 cell effector populations. As T cells from CD46-deficient patients proliferate normally (not shown), these differences likely relate to non-bioenergetic aspects of subset-specific metabolic reprogramming. Indeed, recent work shows that expression of the pyruvate dehydrogenase (PDH) kinase 1 in Th17, but not Th1, cells is a hallmark of their distinct metabolic programs (Gerriets et al., 2015). Our observation that cleaved cytoplasmic domains of CD46 translocated to the nucleus makes it a possibility that they function within transcription factor- or regulator-complexes, directly controlling metabolism.

Complement is among the evolutionary oldest effector immune systems and preceded the appearance of B and T cells

Figure 5. LAMTOR5 Is Required for mTORC1 Complex Assembly in Human $CD4^+$ T Cells

(A–C) LAMTOR5 expression in T cells from (A) a healthy donor (HD) assessed by western blotting, (B) in patient CD46-3 and a HD by FACS analysis, and in (C) HD T cells treated with CD46-specific siRNA at 72 hr post activation.

(D) CD46 activation increases LAMTOR5-dependent assembly of the lysosome-based machinery enabling amino acid sensing via mTORC1 as assessed at 36 hr post activation by confocal microscopy. For the HDs, one representative example is shown for n = 7. Staining of RAGC could not be performed on cells from patient CD46-3.

(E) Statistical analysis for the colocalization events in HD T cells of the proteins assessed under (D) (n = 7).

(F) Reduction of LAMTOR5 expression prevents normal mTORC1 assembly measured at 36 hr post activation by (F) confocal microscopy, and (G) colocalization of proteins measured with the Pearson's Correlation Coefficient method. Results shown in (F) and (G) are representative n = 5.

Magnification (C and E) × 100. $^*p < 0.05$; $^{**}p < 0.01$; $^{***}p < 0.005$. Error bars represent mean ± SEM. See also Figure S5.

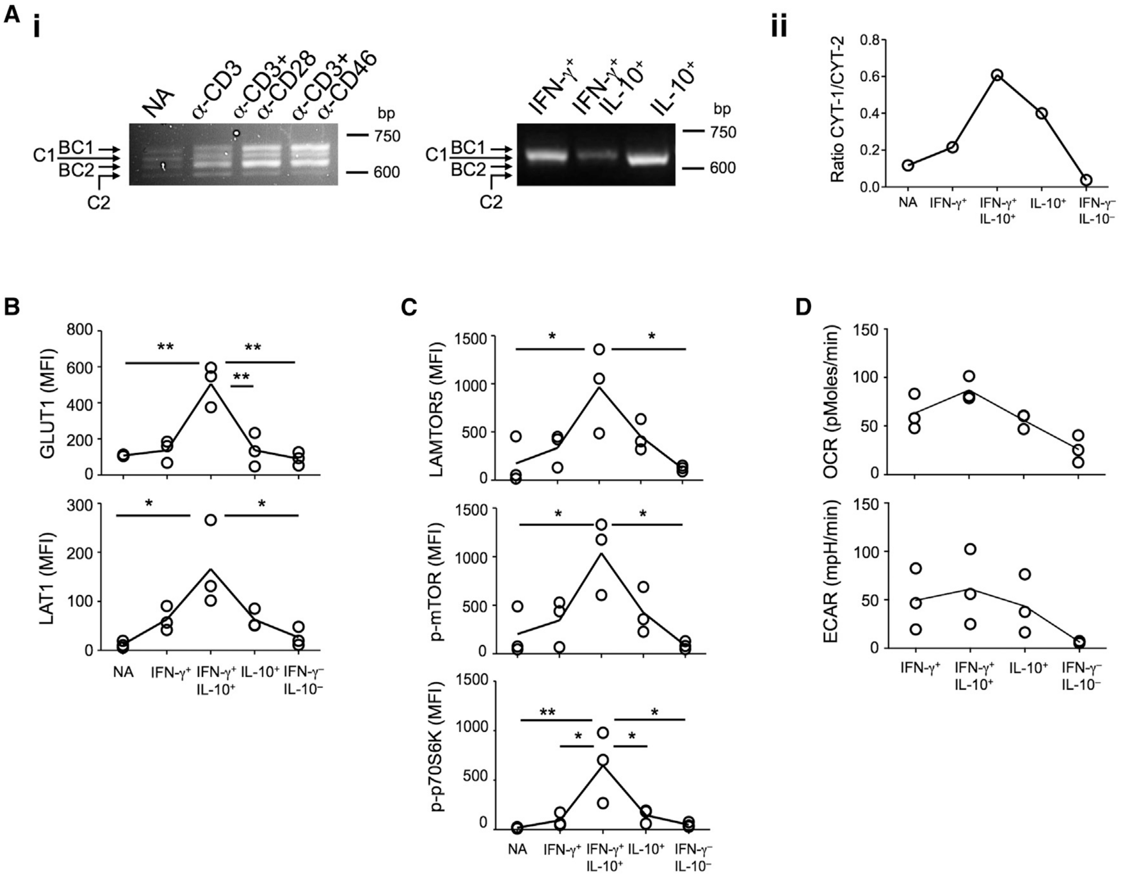

Figure 7. Switches in CD46 Isoform-Expression Correlate with Expected Metabolic Changes during the Th1 Cell Life Cycle

(A) CD46 isoform mRNA levels in (Ai) non-activated (NA) and activated T cells (36 hr, left panel) and in sorted IFN-γ^+, IFN-γ^+IL-10$^+$, and IL-10$^+$ Th1 cell subpopulations (activated for 36 hr, right panel), and (Aii) ratio of CYT-1 to CYT-2 tail mRNA expression.

(B–D) Nutrient channel expression, mTORC1 activity and glycolysis and OXPHOS levels in IFN-γ^+, IFN-γ^+IL-10$^+$, and IL-10$^+$ Th1 cell subpopulations.

Data in (A)–(D) are derived from n = 3. $^*p < 0.05$; $^{**}p < 0.01$. See also Figure S6.

(Le Friec and Kemper, 2009). The recent discovery that complement activation and function also occurs within cells evokes the possibility that complement evolved initially as an intracellular stress detection system (Kolev et al., 2014). A link between complement and metabolic checkpoint kinases, such as mTOR, is thus plausible. The possibility for such a functional cooperation is further supported by the fact that CD46 is a key constituent of a sensory network integrating signals to respond to variable nutrient environments. For example, Notch regulates OXPHOS and glycolysis in cancer cells, pre-T cells, and memory cells (Ciofani and Zúñiga-Pflücker, 2005; Landor et al., 2011; Maekawa et al., 2015), and we have previously shown that interaction of CD46 and the Notch-family member Jagged1 regulates Th1 cell activation (Le Friec et al., 2012). Furthermore, CD46 facilitates the assembly of the IL-2 and IL-7 receptor complexes in T cells (Le Friec et al., 2012), which contributes to increased GLUT1 expression (Wofford et al., 2008) and integrates "high environmental IL-2" signals into a Th1 cell shutdown program.

Our results may also suggest reevaluating the role of CD46 in infection. CD46 serves as receptor for several pathogens (Cattaneo, 2004) and dogma states that pathogens binding CD46 abuse the receptor's ability to promote IL-10 switching, thus furthering an infection-promoting environment (Cope et al., 2011). However, recent work demonstrates that, in epithelial cells, adenoviruses induce glycolysis, thereby supporting the metabolic demands of viral replication (Thai et al., 2014). Thus, the interplay of pathogens and CD46 may have a "metabolic dimension," and understanding the signals regulating *CD46* mRNA splicing and/or autocrine C3b generation may also deliver novel tools to therapeutically exploit CD4$^+$ T cell metabolic reprogramming.

EXPERIMENTAL PROCEDURES

Donors and Patients

Blood samples were obtained with ethical and institutional approvals (Wandsworth Research Ethics Committee, REC number 09/H0803/154). T cells were purified from buffy coats (NHSBT, Tooting, UK; Blood Donor Centre, Basel, Switzerland) or blood samples from healthy volunteers after informed consent. Three adult CD46-deficient patients with confirmed diagnosis of hemolytic uremic syndrome (HUS) and with clinically low Ig (IgG1 and IgG2) levels and recurrent chest infections (CD46-1 and CD46-3) were recruited in France and blood samples were obtained with local ethical approval (Couzi et al., 2008; Fremeaux-Bacchi et al., 2006; Le Friec et al., 2012). The patients had neither infection nor active hemolytic uremic syndrome at the time of blood sampling. In all experiments that involved T cells from CD46-deficient patients, T cells from age- and sex-matched healthy volunteers were used as controls.

For details on T cell isolation, activation, and cytokine measurements, see Supplemental Experimental Procedures.

Antibodies, Proteins, and Inhibitors

Details are included in the Supplemental Experimental Procedures.

OCR and ECAR Measurements

For analysis of the OCR (in pMoles/min) and ECAR (in mpH/min), the Seahorse XF^{e}-96 (primary cells) or Seahorse XF-24 (Jurkat cell lines) metabolic extracellular flux analyzers were used (Seahorse Bioscience, North Billerica, MA) with detailed instructions in Supplemental Experimental Procedures and metabolic parameters calculated as described in Figure S1.

Glucose and Amino Acid Uptake Assays

Details are included in the Supplemental Experimental Procedures.

Confocal Microscopy and Colocalization Analyses

Assays were performed as previously described (Liszewski et al., 2013), with further details in Supplemental Experimental Procedures.

mRNA Silencing

siRNA targeting human LAMTOR5 (SR307168), CD46 (SR302841), and negative control scrambled siRNA were purchased from Origene (Rockville, MD) and delivered into primary human CD4^{+} T cells by transfection using Lipofectamine RNAiMAX (Life Technologies, Paisley, UK) according to the manufacturer-provided protocol. LAMTOR5 and CD46 protein knockdown were consistently about 70% and between 50%–35%, respectively.

CD46 Isoform-Specific RT-PCR and Lentiviral Transfection of T Cells with CD46 CYT-1 or CYT-2

Details are included in the Supplemental Experimental Procedures.

Gene Arrays and Array Analyses

Transcriptome profiling was performed using Illumina HT12V4 microarrays (Illumina, Great Chesterford, UK) on technical triplicates using CD3+CD46-activated T cells isolated from patient CD46-3 and an age- and sex-matched healthy donor. Expression data were analyzed using Partek Genomics Suite (Partek, St. Louis, USA) version 6.6, Ingenuity Pathway Analysis (QIAGEN) and Gene Set Enrichment Analysis, GSEA (Subramanian et al., 2005) (Broad Institute of MIT and Harvard). For details, see Supplemental Experimental Procedures. The raw data of all arrays are deposited with the Gene Expression Omnibus (GEO) repository under the accession number GEO: GSE69090.

Statistical Analysis

Analyses were performed on GraphPad Prism (La Jolla, CA). Data are presented as mean ± SD or median (interquartile range, IQR) for parametric and non-parametric data, respectively, and compared using paired t tests with Bonferroni correction for multiple comparisons, Wilcoxon signed rank tests, the two-tailed Mann-Whitney test, one-way or two-way ANOVA with a Tukey multiple comparison post hoc test, as appropriate. p values < 0.05 denoted statistical significance throughout.

ACCESSION NUMBERS

The raw data of all arrays are deposited with the Gene Expression Omnibus (GEO) repository under the accession number GEO: GSE69090.

SUPPLEMENTAL INFORMATION

Supplemental Information includes four tables, six figures, and Supplemental Experimental Procedures and can be found with this article online at http://dx.doi.org/10.1016/j.immuni.2015.05.024.

AUTHOR CONTRIBUTIONS

M.K., S.D., and G.L.F. designed and performed GLUT1, LAT1, and LAMTOR5 expression assays, metabolic flux experiments and nuclear translocation experiments and wrote and edited the manuscript. G.A.P. helped with the retroviral transfection "rescue" experiments. A.N. performed the exome sequencing, and G.A. analyzed CD46 and cytokine expression in Jurkat T cells. P.L., G.A., and J.W. performed the gene arrays and P.L. and B.A. analyzed the data and edited the manuscript. M.F., R.B., J.L., L.R., and G.R.B. contributed to metabolic assays. L.C. provided the blood samples from the CD46-deficient patients and discussed the data. C.K and C.H. perceived, designed, and coordinated the study and wrote the manuscript.

ACKNOWLEDGMENTS

We thank the donors and the patients for their support, Margaret So (University of Arizona) for the anti-CD46 CYT-1 and CYT-2 antibodies and Erez Dror (University of Basel) for help with nutrient uptake studies. This work was supported by the MRC grants G1002165 (C.K.) and MR/J006742/1, an EU-funded Innovative Medicines Initiative BTCURE (C.K.), a Wellcome Trust Investigator Award (C.K), a Wellcome Trust Intermediate Research Fellowship (B.A.), the National Institute for Health Research (NIHR) Biomedical Research Centre at Guy's and St Thomas' NHS Foundation Trust, and King's College London. S.D. was supported by the Roche Postdoctoral Fellowship Program, C.H. by the Gebert Rüf Foundation (GRS–058/14), and both C.H. and M.F. by the Swiss National Science Foundation (310030-153059 and 323530-139181, respectively).

Received: July 31, 2014
Revised: March 24, 2015
Accepted: April 10, 2015
Published: June 16, 2015

REFERENCES

Astier, A., Trescol-Biémont, M.C., Azocar, O., Lamouille, B., and Rabourdin-Combe, C. (2000). Cutting edge: CD46, a new costimulatory molecule for T cells, that induces p120CBL and LAT phosphorylation. J. Immunol. *164*, 6091–6095.

Astier, A.L., Meiffren, G., Freeman, S., and Hafler, D.A. (2006). Alterations in CD46-mediated Tr1 regulatory T cells in patients with multiple sclerosis. J. Clin. Invest. *116*, 3252–3257.

Bachar, E., Ariav, Y., Ketzinel-Gilad, M., Cerasi, E., Kaiser, N., and Leibowitz, G. (2009). Glucose amplifies fatty acid-induced endoplasmic reticulum stress in pancreatic beta-cells via activation of mTORC1. PLoS ONE *4*, e4954.

Bar-Peled, L., and Sabatini, D.M. (2014). Regulation of mTORC1 by amino acids. Trends Cell Biol. *24*, 400–406.

Bar-Peled, L., Schweitzer, L.D., Zoncu, R., and Sabatini, D.M. (2012). Ragulator is a GEF for the rag GTPases that signal amino acid levels to mTORC1. Cell *150*, 1196–1208.

Bhaskar, P.T., Nogueira, V., Patra, K.C., Jeon, S.M., Park, Y., Robey, R.B., and Hay, N. (2009). mTORC1 hyperactivity inhibits serum deprivation-induced apoptosis via increased hexokinase II and GLUT1 expression, sustained Mcl-1 expression, and glycogen synthase kinase 3beta inhibition. Mol. Cell. Biol. *29*, 5136–5147.

Cardone, J., Le Friec, G., Vantourout, P., Roberts, A., Fuchs, A., Jackson, I., Suddason, T., Lord, G., Atkinson, J.P., Cope, A., et al. (2010). Complement regulator CD46 temporally regulates cytokine production by conventional and unconventional T cells. Nat. Immunol. *11*, 862–871.

Cattaneo, R. (2004). Four viruses, two bacteria, and one receptor: membrane cofactor protein (CD46) as pathogens' magnet. J. Virol. *78*, 4385–4388.

Chang, C.H., Curtis, J.D., Maggi, L.B., Jr., Faubert, B., Villarino, A.V., O'Sullivan, D., Huang, S.C., van der Windt, G.J., Blagih, J., Qiu, J., et al. (2013). Posttranscriptional control of T cell effector function by aerobic glycolysis. Cell *153*, 1239–1251.

Ciofani, M., and Zúñiga-Pflücker, J.C. (2005). Notch promotes survival of pre-T cells at the beta-selection checkpoint by regulating cellular metabolism. Nat. Immunol. *6*, 881–888.

Cope, A., Le Friec, G., Cardone, J., and Kemper, C. (2011). The Th1 life cycle: molecular control of IFN-γ to IL-10 switching. Trends Immunol. *32*, 278–286.

CellPress

Couzi, L., Contin-Bordes, C., Marliot, F., Sarrat, A., Grimal, P., Moreau, J.F., Merville, P., and Fremeaux-Bacchi, V. (2008). Inherited deficiency of membrane cofactor protein expression and varying manifestations of recurrent atypical hemolytic uremic syndrome in a sibling pair. Am. J. Kidney Dis. *52*, e5–e9.

Cunningham, J.T., Rodgers, J.T., Arlow, D.H., Vazquez, F., Mootha, V.K., and Puigserver, P. (2007). mTOR controls mitochondrial oxidative function through a YY1-PGC-1alpha transcriptional complex. Nature *450*, 736–740.

Delgoffe, G.M., Kole, T.P., Zheng, Y., Zarek, P.E., Matthews, K.L., Xiao, B., Worley, P.F., Kozma, S.C., and Powell, J.D. (2009). The mTOR kinase differentially regulates effector and regulatory T cell lineage commitment. Immunity *30*, 832–844.

Düvel, K., Yecies, J.L., Menon, S., Raman, P., Lipovsky, A.I., Souza, A.L., Triantafellow, E., Ma, Q., Gorski, R., Cleaver, S., et al. (2010). Activation of a metabolic gene regulatory network downstream of mTOR complex 1. Mol. Cell *39*, 171–183.

Frauwirth, K.A., Riley, J.L., Harris, M.H., Parry, R.V., Rathmell, J.C., Plas, D.R., Elstrom, R.L., June, C.H., and Thompson, C.B. (2002). The CD28 signaling pathway regulates glucose metabolism. Immunity *16*, 769–777.

Fremeaux-Bacchi, V., Moulton, E.A., Kavanagh, D., Dragon-Durey, M.A., Blouin, J., Caudy, A., Arzouk, N., Cleper, R., Francois, M., Guest, G., et al. (2006). Genetic and functional analyses of membrane cofactor protein (CD46) mutations in atypical hemolytic uremic syndrome. J. Am. Soc. Nephrol. *17*, 2017–2025.

Gerriets, V.A., and Rathmell, J.C. (2012). Metabolic pathways in T cell fate and function. Trends Immunol. *33*, 168–173.

Gerriets, V.A., Kishton, R.J., Nichols, A.G., Macintyre, A.N., Inoue, M., Ilkayeva, O., Winter, P.S., Liu, X., Priyadharshini, B., Slawinska, M.E., et al. (2015). Metabolic programming and PDHK1 control CD4+ T cell subsets and inflammation. J. Clin. Invest. *125*, 194–207.

Groenewoud, M.J., and Zwartkruis, F.J. (2013). Rheb and Rags come together at the lysosome to activate mTORC1. Biochem. Soc. Trans. *41*, 951–955.

Gubser, P.M., Bantug, G.R., Razik, L., Fischer, M., Dimeloe, S., Hoenger, G., Durovic, B., Jauch, A., and Hess, C. (2013). Rapid effector function of memory CD8+ T cells requires an immediate-early glycolytic switch. Nat. Immunol. *14*, 1064–1072.

Hayashi, K., Jutabha, P., Endou, H., Sagara, H., and Anzai, N. (2013). LAT1 is a critical transporter of essential amino acids for immune reactions in activated human T cells. J. Immunol. *191*, 4080–4085.

Jacobs, S.R., Herman, C.E., Maciver, N.J., Wofford, J.A., Wieman, H.L., Hammen, J.J., and Rathmell, J.C. (2008). Glucose uptake is limiting in T cell activation and requires CD28-mediated Akt-dependent and independent pathways. J. Immunol. *180*, 4476–4486.

Jones, R.G., and Thompson, C.B. (2007). Revving the engine: signal transduction fuels T cell activation. Immunity *27*, 173–178.

Kemper, C., Leung, M., Stephensen, C.B., Pinkert, C.A., Liszewski, M.K., Cattaneo, R., and Atkinson, J.P. (2001). Membrane cofactor protein (MCP; CD46) expression in transgenic mice. Clin. Exp. Immunol. *124*, 180–189.

Kemper, C., Chan, A.C., Green, J.M., Brett, K.A., Murphy, K.M., and Atkinson, J.P. (2003). Activation of human CD4+ cells with CD3 and CD46 induces a T-regulatory cell 1 phenotype. Nature *421*, 388–392.

Kolev, M., Le Friec, G., and Kemper, C. (2014). Complement—tapping into new sites and effector systems. Nat. Rev. Immunol. *14*, 811–820.

Landor, S.K., Mutvei, A.P., Mamaeva, V., Jin, S., Busk, M., Borra, R., Grönroos, T.J., Kronqvist, P., Lendahl, U., and Sahlgren, C.M. (2011). Hypo- and hyperactivated Notch signaling induce a glycolytic switch through distinct mechanisms. Proc. Natl. Acad. Sci. USA *108*, 18814–18819.

Le Friec, G., and Kemper, C. (2009). Complement: coming full circle. Arch. Immunol. Ther. Exp. (Warsz.) *57*, 393–407.

Le Friec, G., Sheppard, D., Whiteman, P., Karsten, C.M., Shamoun, S.A., Laing, A., Bugeon, L., Dallman, M.J., Melchionna, T., Chillakuri, C., et al. (2012). The CD46-Jagged1 interaction is critical for human TH1 immunity. Nat. Immunol. *13*, 1213–1221.

Liszewski, M.K., and Atkinson, J.P. (1996). Membrane cofactor protein (MCP; CD46). Isoforms differ in protection against the classical pathway of complement. J. Immunol. *156*, 4415–4421.

Liszewski, M.K., Post, T.W., and Atkinson, J.P. (1991). Membrane cofactor protein (MCP or CD46): newest member of the regulators of complement activation gene cluster. Annu. Rev. Immunol. *9*, 431–455.

Liszewski, M.K., Kolev, M., Le Friec, G., Leung, M., Bertram, P.G., Fara, A.F., Subias, M., Pickering, M.C., Drouet, C., Meri, S., et al. (2013). Intracellular complement activation sustains T cell homeostasis and mediates effector differentiation. Immunity *39*, 1143–1157.

Long, X., Ortiz-Vega, S., Lin, Y., and Avruch, J. (2005). Rheb binding to mammalian target of rapamycin (mTOR) is regulated by amino acid sufficiency. J. Biol. Chem. *280*, 23433–23436.

Macintyre, A.N., Gerriets, V.A., Nichols, A.G., Michalek, R.D., Rudolph, M.C., Deoliveira, D., Anderson, S.M., Abel, E.D., Chen, B.J., Hale, L.P., and Rathmell, J.C. (2014). The glucose transporter Glut1 is selectively essential for CD4 T cell activation and effector function. Cell Metab. *20*, 61–72.

MacIver, N.J., Michalek, R.D., and Rathmell, J.C. (2013). Metabolic regulation of T lymphocytes. Annu. Rev. Immunol. *31*, 259–283.

Maekawa, Y., Ishifune, C., Tsukumo, S., Hozumi, K., Yagita, H., and Yasutomo, K. (2015). Notch controls the survival of memory CD4+ T cells by regulating glucose uptake. Nat. Med. *21*, 55–61.

Marinari, B., Costanzo, A., Marzano, V., Piccolella, E., and Tuosto, L. (2004). CD28 delivers a unique signal leading to the selective recruitment of RelA and p52 NF-kappaB subunits on IL-8 and Bcl-xL gene promoters. Proc. Natl. Acad. Sci. USA *101*, 6098–6103.

Matheny, R.W., Jr., and Adamo, M.L. (2009). Effects of PI3K catalytic subunit and Akt isoform deficiency on mTOR and p70S6K activation in myoblasts. Biochem. Biophys. Res. Commun. *390*, 252–257.

Michalek, R.D., Gerriets, V.A., Jacobs, S.R., Macintyre, A.N., MacIver, N.J., Mason, E.F., Sullivan, S.A., Nichols, A.G., and Rathmell, J.C. (2011). Cutting edge: distinct glycolytic and lipid oxidative metabolic programs are essential for effector and regulatory CD4+ T cell subsets. J. Immunol. *186*, 3299–3303.

Ni Choileain, S., Weyand, N.J., Neumann, C., Thomas, J., So, M., and Astier, A.L. (2011). The dynamic processing of CD46 intracellular domains provides a molecular rheostat for T cell activation. PLoS ONE *6*, e16287.

Pearce, E.L., Poffenberger, M.C., Chang, C.H., and Jones, R.G. (2013). Fueling immunity: insights into metabolism and lymphocyte function. Science *342*, 1242454.

Pollizzi, K.N., and Powell, J.D. (2014). Integrating canonical and metabolic signalling programmes in the regulation of T cell responses. Nat. Rev. Immunol. *14*, 435–446.

Rathmell, J.C. (2012). Metabolism and autophagy in the immune system: immunometabolism comes of age. Immunol. Rev. *249*, 5–13.

Roos, S., Kanai, Y., Prasad, P.D., Powell, T.L., and Jansson, T. (2009). Regulation of placental amino acid transporter activity by mammalian target of rapamycin. Am. J. Physiol. Cell Physiol. *296*, C142–C150.

Sancak, Y., Bar-Peled, L., Zoncu, R., Markhard, A.L., Nada, S., and Sabatini, D.M. (2010). Ragulator-Rag complex targets mTORC1 to the lysosomal surface and is necessary for its activation by amino acids. Cell *141*, 290–303.

Sena, L.A., Li, S., Jairaman, A., Prakriya, M., Ezponda, T., Hildeman, D.A., Wang, C.R., Schumacker, P.T., Licht, J.D., Perlman, H., et al. (2013). Mitochondria are required for antigen-specific T cell activation through reactive oxygen species signaling. Immunity *38*, 225–236.

Shi, L.Z., Wang, R., Huang, G., Vogel, P., Neale, G., Green, D.R., and Chi, H. (2011). HIF1alpha-dependent glycolytic pathway orchestrates a metabolic checkpoint for the differentiation of TH17 and Treg cells. J. Exp. Med. *208*, 1367–1376.

Sinclair, L.V., Rolf, J., Emslie, E., Shi, Y.B., Taylor, P.M., and Cantrell, D.A. (2013). Control of amino-acid transport by antigen receptors coordinates the metabolic reprogramming essential for T cell differentiation. Nat. Immunol. *14*, 500–508.

Subramanian, A., Tamayo, P., Mootha, V.K., Mukherjee, S., Ebert, B.L., Gillette, M.A., Paulovich, A., Pomeroy, S.L., Golub, T.R., Lander, E.S., and Mesirov, J.P. (2005). Gene set enrichment analysis: a knowledge-based approach for interpreting genome-wide expression profiles. Proc. Natl. Acad. Sci. USA *102*, 15545–15550.

Thai, M., Graham, N.A., Braas, D., Nehil, M., Komisopoulou, E., Kurdistani, S.K., McCormick, F., Graeber, T.G., and Christofk, H.R. (2014). Adenovirus E4ORF1-induced MYC activation promotes host cell anabolic glucose metabolism and virus replication. Cell Metab. *19*, 694–701.

van der Windt, G.J., Everts, B., Chang, C.H., Curtis, J.D., Freitas, T.C., Amiel, E., Pearce, E.J., and Pearce, E.L. (2012). Mitochondrial respiratory capacity is a critical regulator of CD8+ T cell memory development. Immunity *36*, 68–78.

van der Windt, G.J., O'Sullivan, D., Everts, B., Huang, S.C., Buck, M.D., Curtis, J.D., Chang, C.H., Smith, A.M., Ai, T., Faubert, B., et al. (2013). CD8 memory T cells have a bioenergetic advantage that underlies their rapid recall ability. Proc. Natl. Acad. Sci. USA *110*, 14336–14341.

Viola, A., and Lanzavecchia, A. (1996). T cell activation determined by T cell receptor number and tunable thresholds. Science *273*, 104–106.

Wang, R., Dillon, C.P., Shi, L.Z., Milasta, S., Carter, R., Finkelstein, D., McCormick, L.L., Fitzgerald, P., Chi, H., Munger, J., and Green, D.R. (2011). The transcription factor Myc controls metabolic reprogramming upon T lymphocyte activation. Immunity *35*, 871–882.

Wofford, J.A., Wieman, H.L., Jacobs, S.R., Zhao, Y., and Rathmell, J.C. (2008). IL-7 promotes Glut1 trafficking and glucose uptake via STAT5-mediated activation of Akt to support T-cell survival. Blood *111*, 2101–2111.

Zheng, Y., Collins, S.L., Lutz, M.A., Allen, A.N., Kole, T.P., Zarek, P.E., and Powell, J.D. (2007). A role for mammalian target of rapamycin in regulating T cell activation versus anergy. J. Immunol. *178*, 2163–2170.

Immunity

Supplemental Information

Complement Regulates Nutrient Influx and Metabolic Reprogramming during Th1 Cell Responses

Martin Kolev, Sarah Dimeloe, Gaelle Le Friec, Alexander Navarini, Giuseppina Arbore, Giovanni A. Povoleri, Marco Fischer, Reka Belle, Jordan Loeliger, Leyla Razik, Glenn R. Bantug, Julie Watson, Lionel Couzi, Behdad Afzali, Paul Lavender, Christoph Hess, and Claudia Kemper

Figure S1, related to Figure 1

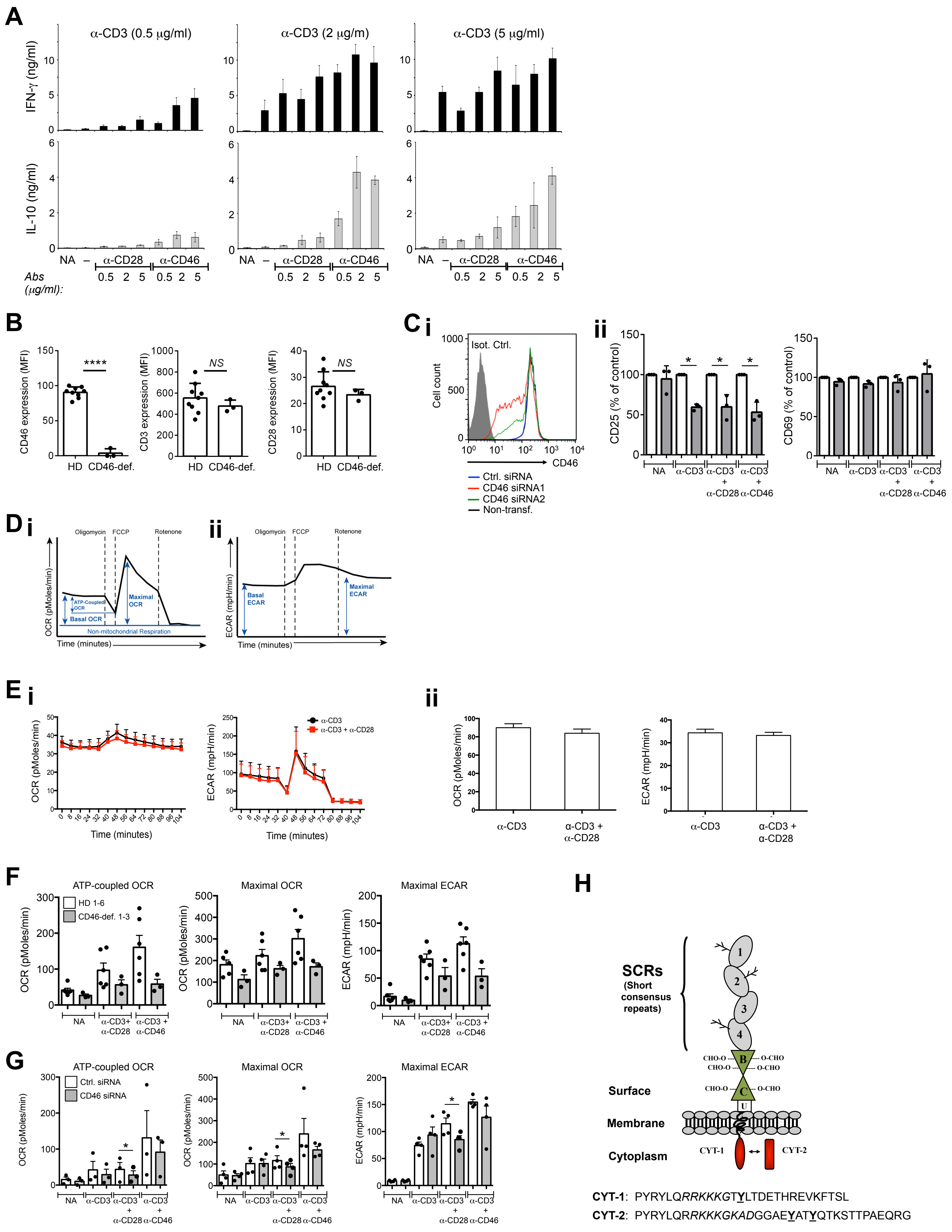

Figure S1, related to Figure 1. CD46 isoforms bearing cytoplasmic domain 1 are required for Th1 induction (IFN-γ secretion) in $CD4^+$ T cells. (A) TCR and CD28-induced Th1 cytokine production correlates with CD46 ligand generation (Figure 1A). Effects of anti-CD3 and anti-CD28 antibody titration on IFN-γ production and IL-10 switching in T cells from healthy donors. Freshly purified $CD4^+$ T cells were either left non-activated (NA) or activated by plate immobilized Abs as depicted and cytokine production measured at 36 h post activation. Shown are results ± SD obtained in n = 3 independently performed experiments using a different donor each time. (B) TCR (CD3), CD28 and CD46 expression levels on T cells isolated from nine age- and sex-matched healthy donors (HD) and from CD46-deficient patients CD46-1, -2 and -3. Patient CD46-1 and CD46-2 had 10% of normal CD46 expression levels on T cells and surface CD46 expression was undetectable in patient CD46-3. (C) Reduced CD46 protein expression in T cells from HDs leads to a significant decrease in CD25 expression upon activation. Purified T cells were (Ci) transfected with a CD46-specific siRNA or a scrambled control siRNA and (Cii) CD25 and CD69 expression measured at 36 h post activation. Shown are results ± SD obtained in n = 4 independently performed experiments. (D) Schematic diagram of oxygen consumption rate (OCR) and extracellular acidification rate (ECAR) profiles as generated by the Seahorse extracellular flux analyzer. (D, left panel) Schematic OCR time course under basal conditions and following perturbation of mitochondrial respiration with oligomycin, FCCP and rotenone. Using this perturbation profiling technique, four OCR rates are directly measured: the non-corrected basal OCR [OCR(basal-nc)], the rate following inhibition of ATP synthase [OCR(oligomycin)], the peak rate following mitochondrial uncoupling [OCR(peak-FCCP)], and the rate following inhibition of mitochondrial respiration [OCR(rotenone)]. The following respiratory parameters (indicated by blue double-ended arrows in the diagram) are calculated using the formulas below:

(1) basal respiration = [OCR(basal-nc)] – [OCR(rotenone)]

(2) ATP coupled respiration = [OCR(basal-nc)] – [OCR(oligomycin)]

(3) maximal respiratory capacity = [OCR(peak-FCCP)] – [OCR(rotenone)]

(D, right panle) Schematic ECAR time course under basal conditions and following inhibition of mitochondrial respiration. Basal ECAR is the initial rate measured by the extracellular flux analyzer. The maximal ECAR is the rate following addition of rotenone. Blue double-ended arrows in the diagram indicate the respective glycolytic parameters. (E) Oxidative phosphorylation (OCR) and glycolysis (ECAR) rates in T cells activated with anti-CD3 or anti-CD3 and anti-CD28 mAb for 36 h. The right panel depicts basal OCR and ECAR. Data shown

are mean ± SD derived from three donors (n = 3). (F) ATP coupled and maximal OCR and maximal ECAR in non-activated and activated $CD4^+$ T cells isolated from CD46-deficient patients CD46-1, -2, and -3 compared to T cells from six HDs, as assessed using the XF Seahorse Analyzer and calculated as described in S1D. (G) Experiments performed as under (F) but using T cells from four different HDs treated with either control siRNA or CD46-specific siRNA. (H) Schematic of the CD46 protein structure. The four major isoforms of CD46 are generated by alternative splicing of the extracellular 'BC' region (either 'C' or 'BC') and the intracellular domains (CYT-1 or CYT-2) of an mRNA transcribed from a single gene. The amino acid sequence for CYT-1 and CYT-2 is shown below with putative nuclear targeting signals depicted with italic letters and tyrosine phosphorylation sites within CYT-1 and CYT-2, 'Y', marked in bold. Candidate kinases include casein kinase 2 (CK-2) and protein kinase C (PKC) for CYT-1, and Src kinases and CK-2 for CYT-2. *, $p < 0.01$; ****, $p < 0.001$; NS, not statistically significant.

Figure S2, related to Figure 2

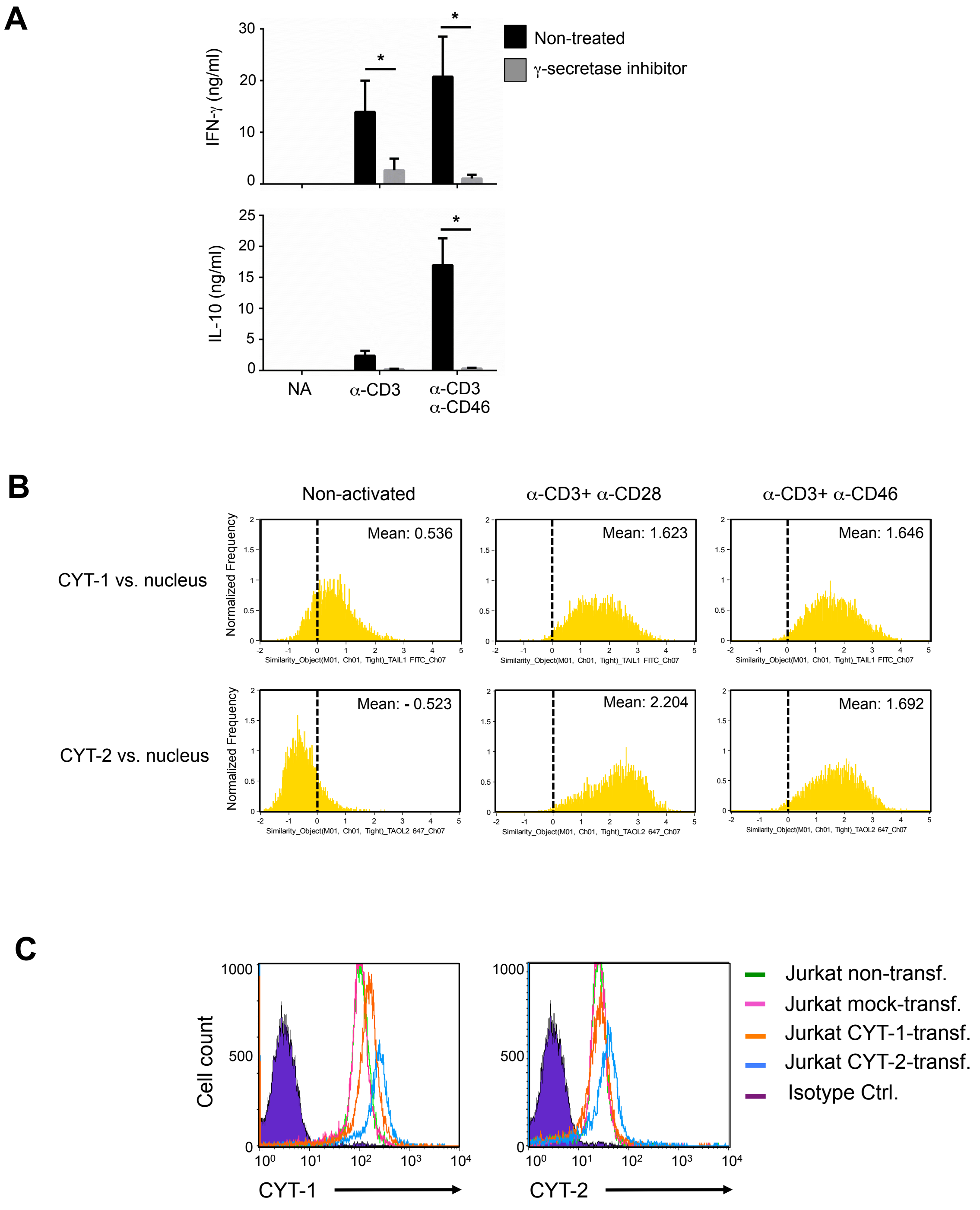

Figure S2, related to Figure 2. CD46 function requires processing and nuclear translocation of its intracellular domains. (A) Inhibition of γ-secretase activity prevents Th1induction. $CD4^{+}$ T cells were activated as depicted with or without addition of a γ-secretase inhibitor (100 nM) and cytokine production measured 36 h post activation. Shown are data ± SD derived from three (n = 3) independently performed experiments. (B) CD46 nuclear domains CYT-1 and CYT-2

translocate into the nucleus upon T cell activation. T cells were left non-activated (NA) or activated as indicated for 2 hrs, permeabilized and stained with CYT-1- and CYT-2-specific antibodies and nuclear translocation of the CD46 tails assessed using Image Stream. FACS plots present correlations between CYT-1 and CYT-2 pixel intensity and intensity of nuclei as measure of colocalization. Shown is one representative data set of n = 3 similarly performed experiments. (C) CYT-1 and CYT-2 expression in non-activated Jurkat T cells 48 h post transfection with retrovirus expressing either CYT-1 or CYT-2 (n = 3). *, $p < 0.05$.

Figure S3, related to Figure 3

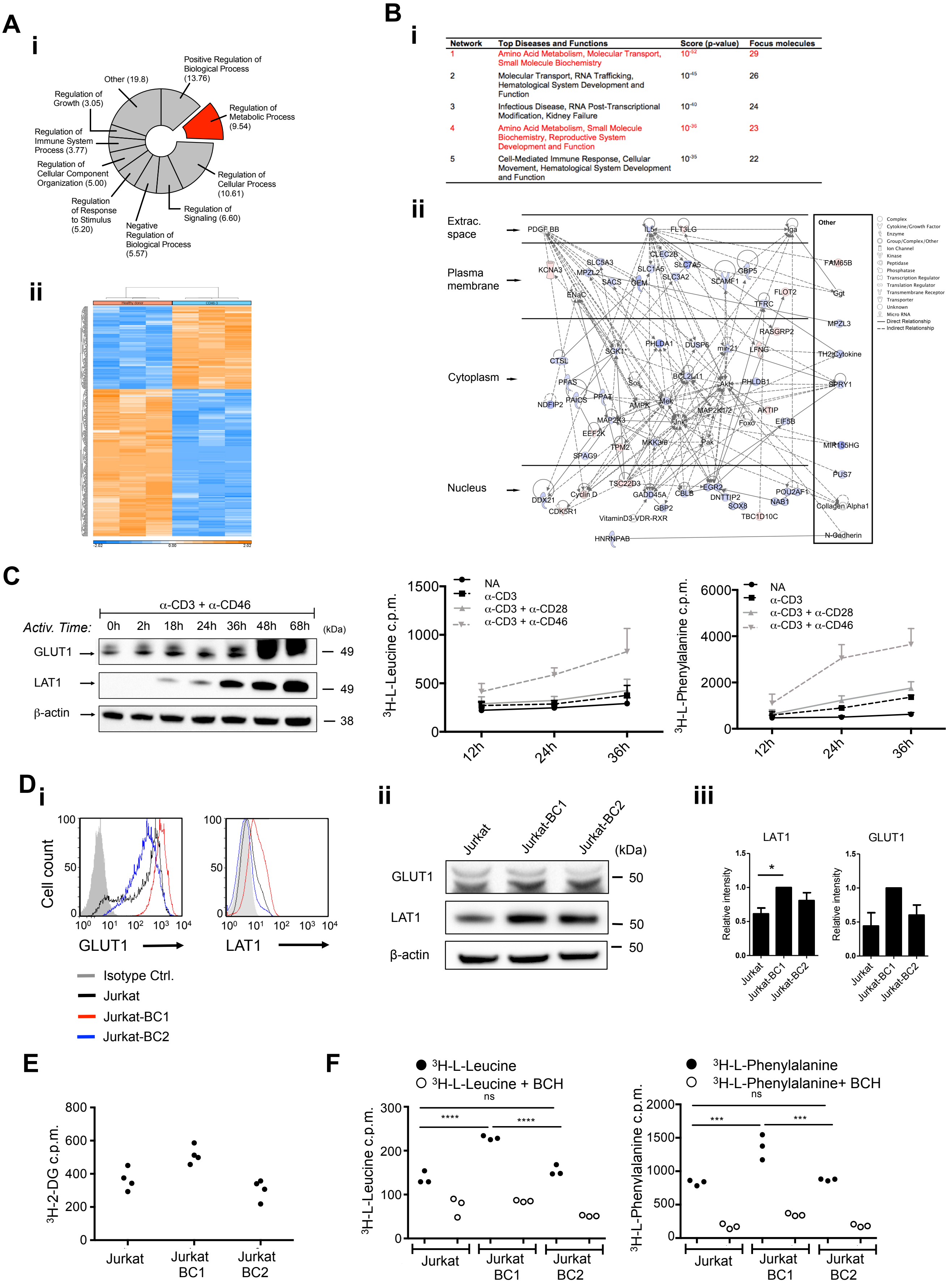

Figure S3, related to Figure 3. CD46 CYT-1 mediates glucose and amino acid influx in $CD4^+$ T cells via GLUT1 and LAT1. (A-B) Analysis of 403 differentially expressed genes between healthy donor (HD) and patient CD46-3, showing (Ai) GO enrichment scores for GO terms under "Regulation of Biological Process" (GO ID 50789) - highlighted is "Regulation of Metabolic Process" (GO ID 19222; p<0.01), (Aii) heatmap of genes in the highlighted segment of (Ai) and (B) Ingenuity Pathway Analysis (IPA) output showing (Bi) the top 5 gene networks and (Bii) schematic of merged gene networks 1 and 4, which are functionally associated with amino acid metabolism (Bi). (C) Time course of GLUT1 and LAT1 expression and amino acid uptake in activated T cells. Purified T cells were activated with antibodies to CD3 and CD46. At indicated time points, expression of GLUT1 and LAT1 was measured by Western blotting (left panel) and amino acid uptake was assessed (right panel). The Western blot is representative of three independently performed experiments and data shown in the lower panel are mean ± SD derived from n = 3 different donors. (D) Expression of GLUT1 and LAT1 in resting Jurkat, Jurkat-BC1 and Jurkat-BC2 cells assessed by (Di) FACS analysis and by (Dii) Western blotting, with (Diii) densitometric analyses of the band intensities obtained in (Dii). Data shown are representative of n = 3 independently performed experiments. (E and F) CD46-BC1 over-expression increases glucose and amino acid uptake in Jurkat T cells. Jurkat cells, Jurkat-BC1, and Jurkat-BC2 cells were incubated with (E) tritium labeled 2-deoxyglucose (^{3}H 2-DG) or (F) L-Leucine (^{3}H L-Leucine) or L-Phenylalanine (^{3}H L-Phenylalanine) in the presence or absence of the LAT1 inhibitor BCH and glucose and amino acid uptake measured, respectively. Data shown are from four (E, n = 4) or three (F, n = 3) experiments. *, $p < 0.05$; *** $p < 0.005$; ****, $p < 0.001$; ns, statistically not significant.

Figure S4, related to Figure 4

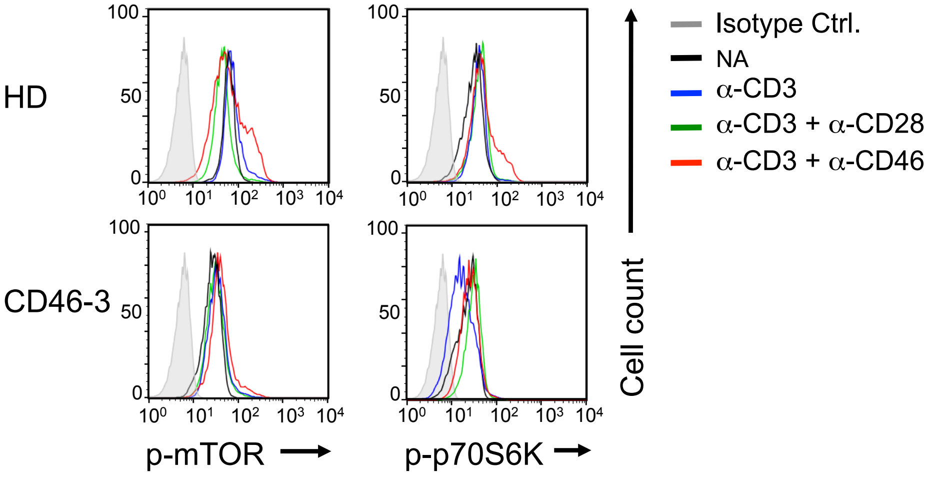

Figure S4, related to Figure 4. CD46 is required for normal p70S6K phosphorylation in activated T cells. Abundance of phosphorylated mTOR (p-mTOR) and p70S6K (p-p70S6K) in non-activated (NA) and activated T cells isolated from four different healthy donors (HD1-4) and from patient CD46-3 at 36 h post activation.

Figure S5, related to Figure 5

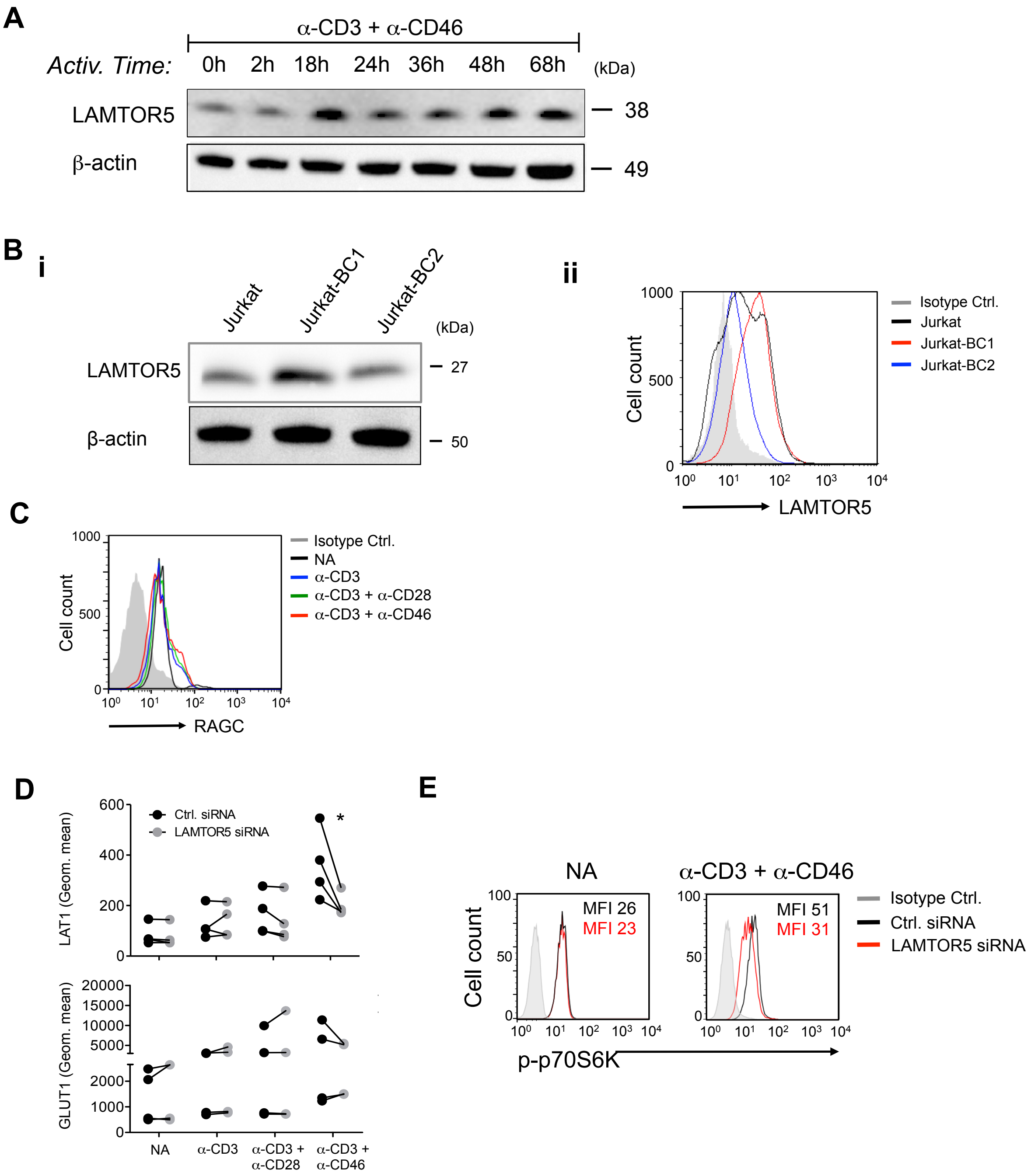

Figure S5, related to Figure 5. Activation-induced LAMTOR5 expression is required for TORC1-mediated p70S6K phosphorylation. (A) Time course of LAMTOR5 expression in activated CD4$^+$ T cells. Purified human CD4$^+$ T cells were activated with antibodies to CD3 and CD46, and LAMTOR5 expression analyzed by Western blotting at indicated time points. Shown is one representative result of n = 3 similarly performed experiments. (B) Comparison of LAMTOR5 expression in resting Jurkat, Jurkat-BC1 and Jurkat-BC2 cells assessed by (Bi)

Western blotting, and by (Bii) FACS analysis. Data shown are representative of n = 3 independently performed experiments. (C) Expression of RAGC in activated T cells at 36 h post activation. Shown is one representative result of n = 2 similarly performed experiments. (D and E) Effect of reduction of LAMTOR5 protein expression by RNA silencing technique on (D) GLUT1 and LAT1 expression and (E) p70S6K phosphorylation in CD3+CD46-activated primary T cells 36 h post activation Data shown are representative of n = 3 or n = 4 independently performed experiments.

Figure S6, related to Figure 7

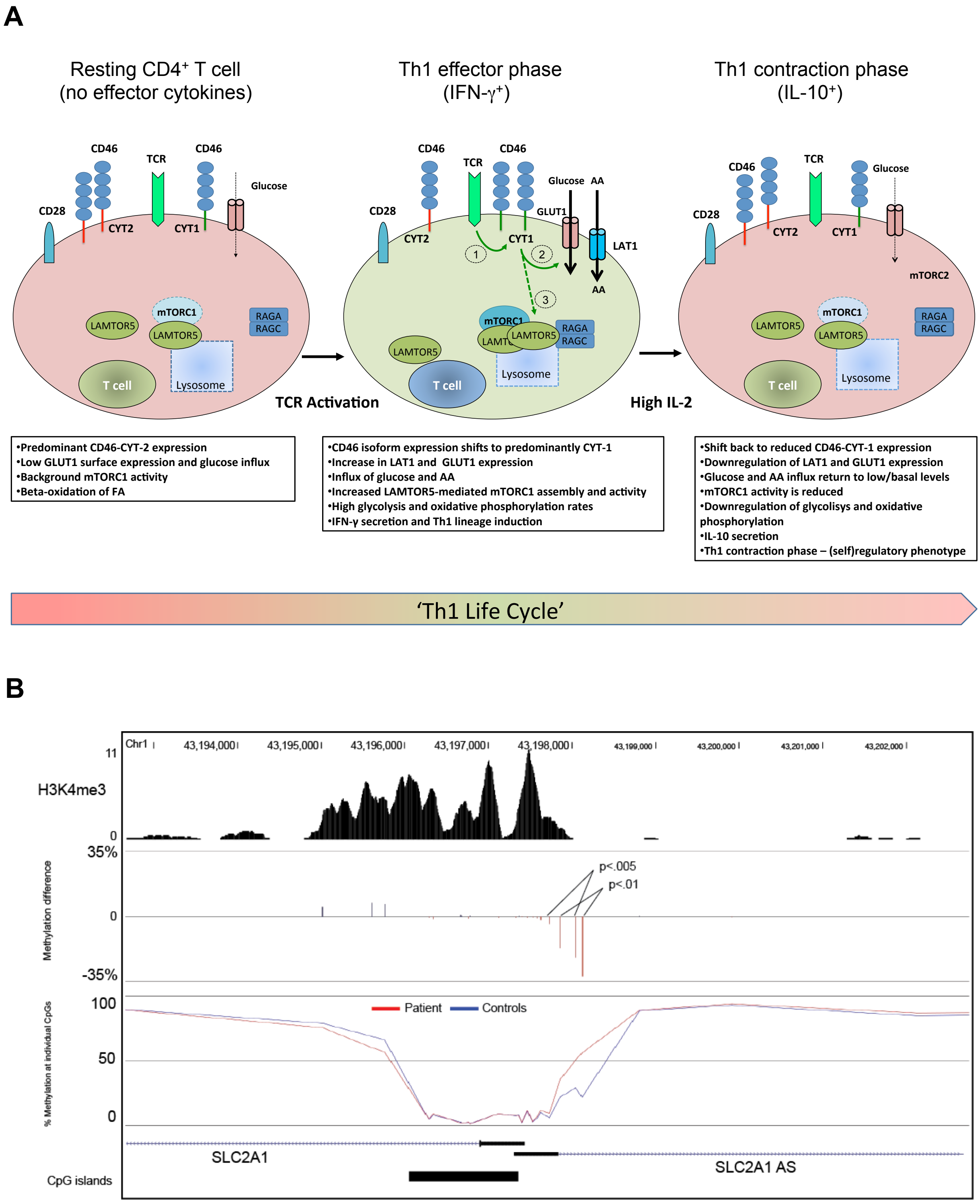

Figure S6, related to Figure 7. The role of CD46 isoforms in the metabolic switch during the Th1 life cycle. (A) Model of the CD46-mediated contributions to key metabolic events required for normal Th1 effector induction. T cell receptor activation of resting T cells induces the local generation of CD46 ligand C3b (not shown) as well as (1) increased expression of CD46 isoforms bearing CYT-1. CD46 CYT-1-driven signals then lead to (2) upregulation of the

glucose transporter GLUT1 and, more importantly, the amino acid channel LAT1, allowing for increased influx of glucose and amino acids into the cell. In parallel, CD46-activated T cells induce increased expression of LAMTOR5 either via CD46-mediated direct or indirect signals (indicated by dotted arriws), and via this assembly of the lysosome-based machinery enabling amino acid sensing via mTORC1, which then leads to key downstream events such as the phosphorylation of p70S6K and induction of glycolysis and oxidative phosphorylation (OXPHOS) required for IFN-γ production. During 'Th1 contraction' and induction of IL-10 co-expression, CD46 isoform expression reverts to a CYT-2 predominant pattern, accompanied by reduced expression of GLUT1 and LAT1, and downregulation of glycolysis and OXPHOS. (B) Increased DNA methylation around the SLC2A1/SLC2A1-AS (GLUT1) bidirectional promoter in CD46-deficient patient CD46-3. DNA methylation was analyzed using bisulphite conversion of cytosine residues within genomic DNA isolated from resting T effector cells from three healthy controls and from T cells collected from CD46-3 at two different times (one year apart). Methylation was quantified on the Illumina Infinium 450K platform. A cluster of CpG dinucleotides showed differential methylation (cg06792911, 7% Control and 11% Patient, $p<0.005$; cg12656391, 18% Control and 35% Patient, $p<0.01$; cg00102166, 27% Control and 50% Patient, $p<0.005$; cg09824328, 22% Control and 54% Patient, $p<0.01$) and coincided with the SLC2A1/SLC2A1-AS bidirectional promoter, the extent of which was marked with H3K4me3 in ChIPseq experiments using mononucleosomal chromatin from polarized primary T cells.

SUPPLEMENTAL EXPERIMENTAL PROCEDURES

Human CD46-transgenic mice

Mice transgenic for human CD46 were previously generated using a YAC containing the human *CD46* gene (Mrkic et al., 1998) with confirmed human CD46-like expression on all assessed nucleated cells including lymphocytes (Kemper et al., 2001).

Antibodies, proteins and inhibitors. Cell-stimulating mAbs were bought from BD Biosciences, San Diego, CA (anti-CD28, CD28.2), purified from a specific hybridoma (anti-CD3; OKT-3) or generated in-house (anti-CD46; TRA-2-10 (Wang et al., 2000). The antibodies to CD46 CYT-1 and CYT-2 were a gift from Dr. Maggie So from the University of Arizona (Weyand et al., 2006), the rabbit polyclonal anti-CD46 serum used in confocal microscopy studies was generated in-house, the PE-anti CD46 (12-0469-42) from eBiosciences (San Diego, CA) and anti-CD69 (561932), anti p-ERK1/2 (612359) and anti-CD25 (555434) from BD. Monoclonal antibodies to LAMP1 (ab24170), GLUT1 (ab115730), HBXIP/LAMTOR5 (ab157480) and β-actin (ab8226) were purchased from Abcam (Cambridge, UK), as was the polyclonal anti-serum to the green fluorescence protein (GFP, ab290). Antibodies recognizing phosphorylated mTOR (2971S), phosphorylated p70S6 Kinase (9205S), human RAGC (3360), Akt phosphorylated at serine 473 (4058), LAT1 (5347) and the mTOR Regulation Antibody Sampler Kit (9864) and the NF-κB Antibody Sampler Kit (9936S) were bought from Cell Signaling Technology (Danvers, MA). mTOR (7C10) conjugated to Alexa Fluor 647 used in confocal microscopy experiments was obtained from Cell Signaling (5048), while the anti-RAGC conjugated to Alexa Fluor 488 (ABIN914033) was from Antibodies Online (Aachen, Germany). Anti-Rabbit IgG (H+L chain Alexa Fluor 594) was from Abcam (ab150076), while anti-mouse IgG Alexa Fluor 488 (A11001), anti-mouse IgG Alexa Fluor 594 (A21125), anti-Rabbit IgG (H+L) Alexa Fluor 488 (A11034), anti-Rabbit IgG (H+L) Alexa Fluor 594 (A11037) and RPE-conjugated anti-Rabbit F(ab')2 fragment (A10542) were obtained from Molecular Probes/Life Sciences (Paisley, UK). Rapamycin was a gift from Dr Cristiano Scotta, King's College London, UK. The amino acid transport inhibitor BCH (2-Amino-2-norbornanecarboxylic acid, A7902), the 2-DG glucose analogue (2-Deoxy-D-glucose, D6134) and the γ-secretase inhibitor (L-685,458) were purchased from Sigma-Aldrich (Saint Louis, MO).

NF-κB reporter system

Jurkat T cells were cotransfected with a luciferase NF-κB reporter plasmid and a renilla control plasmid (E8491; Promega, Madison, WI) for normalization of transfection efficiency between samples. Cells were activated with different stimulating antibodies and luciferase activity assessed in cell lysates 1 - 3 h post activation using the Dual-Luciferase Reporter Assay System (E1919) from Promega.

T cell isolation and activation. PBMCs were separated to $CD4^+$ T cells using the MACS human $CD4^+$ Positive T cell Isolation Kit (Miltenyi Biotech Ltd, Bisley, UK) according to manufacturer's instructions. Purity of isolated lymphocyte fractions was typically >97 %. $CD4^+$ T cells were activated in 48-well culture plates (2.5 – 3.0 x 10^5 cells/well) coated with mAbs to CD3, CD28 or CD46 (2.0 μg/ml PBS each) and addition of 25 U/ml rhIL-2. Cell viability and/or apoptosis was monitored by FACS analysis using Annexin V-APC and Propidium Iodide (both from BD Biosciences, Oxford, UK) staining. The Jurkat T cell lines stably transfected with either the CD46 CYT-1 or CYT-2-bearing isoform have been described previously (Cardone et al., 2010).

Cytokine measurements. Cytokine production by T cells was measured using either the Th1/Th2/Th17 Cytokine Bead Array (560484, BD Biosciences) or the the Human Cytokine Secretion Assay Kits for IFN-γ (130-090-433) and IL-10 (130-090-761) from Miltenyi (Surrey, UK) in combination. IL-8 was measured using the Human IL-8 ELISA Kit (555244) from BD.

OCR and ECAR measurements. For analysis of the OCR (in pMoles/min) and ECAR (in mpH/min), the Seahorse XF-96 (primary cells) or Seahorse XF-24 (Jurkat cell lines) metabolic extracellular flux analyzers were used (Seahorse Bioscience, North Billerica, MA). $CD4^+$ T cells were resuspended in serum-free unbuffered RPMI-1640 medium (R1383, Sigma-Aldrich) and were plated onto Seahorse cell plates (Primary Cells: 2.5 × 10^5 cells/well; Jurkat cell lines: 1.0 x 10^5 cells/well) coated with Cell-Tak (CB-40241, Corning, Reinach, Switzerland) to enhance T cell attachment. Perturbation profiling of the use of metabolic pathways by $CD4^+$ T cells was achieved by the addition of oligomycin (O4876) (1 μM), Carbonyl cyanide-*4*-(trifluoromethoxy)phenylhydrazone (FCCP, C2920) (2 μM) and rotenone (R8875) (1 μM; all from Sigma-Aldrich). Metabolic parameters were then calculated as described in Supplementary Figure 1.

Glucose and amino acid uptake assays. For analysis of glucose and amino acid uptake, 2 × 10^5 $CD4^+$ T cells were resuspended in 200 µl Hank's balanced-salt solution (Gibco) containing either [^{3}H]2-deoxyglucose (NET549, Perkin Elmer, Schwerzenbach, Switzerland), [^{3}H]L-phenylalanine (MT1916) or [^{3}H]L-leucine (MT672) (both Hartmann Analytic, Germany), all at a final concentration of 1 µCi/rxn (5 µCi/ml). Unlabeled 2-deoxyglucose (10 mM, Sigma-Aldrich) or LAT1 inhibitor 2-aminobicyclo-(2,2,1)-heptane-2-carboxylic acid (BCH, 10 mM, Sigma-Aldrich) were included where indicated. Uptake was assayed for 6 min after which the cells were washed twice with ice-cold PBS. Cells were then lysed with 50 µl 0.1 % Sodium dodecyl sulfate (SDS) and the cell lysate (40 µl) combined with 160 µl MicroScint™ 40 scintillation fluid (Perkin Elmer, Waltham, MA). β-radioactivity was measured by liquid scintillation counting in a TopCount Scintillation Counter (Perkin Elmer).

Glucose uptake capacity was also measured in primary cells via uptake of the fluorescent 2-deoxy-D-glucose analog 2-[N-(7-nitrobenz-2-oxa-1,3-diazol-4-yl)amino]-2-deoxy-D-glucose (2-NBDG, Life Technologies, Zug, Switzerland). Briefly, 2 x 10^5 $CD4^+$ T cells were resuspended in 200 µl RPMI/10 % FCS containing 20 µM 2-NBDG. The cells were then incubated for 20 min at 37 °C and 5 % CO_2 prior to washing twice with FACS buffer. Fluorescence at 540 nm was then analysed using the Accuri Flow Cytometer (BD Biosciences).

Confocal Microscopy and colocalization analyses. Fixed and permeabilized cells were stained with the indicated primary antibodies overnight at 4 °C at the manufacturer's suggested dilutions. For mTOR, LAMTOR5 and LAMP1 or RAGC co-staining, cells were first stained with anti-LAMTOR5 or LAMP1 antibodies followed by appropriate secondary antibodies conjugated to Alexa Fluor 488 and 594. After two washes, anti-mTOR antibody conjugated to Alexa Fluor 647 or anti-RAGC conjugated to Alexa Fluor 488 was added for 1 h at room temperature. Where indicated, staining with secondary antibodies was performed for 1 h at room temperature. Cells were mounted using Vectashield mounting media containing DAPI (H-1500, Vector Laboratories, Peterborough, UK) and images were obtained in the KCL Nikon Imaging Centre by confocal fluorescence microscopy with A1R SI Confocal Microscope (360 objective) both from Nikon (Surrey, UK). Pearson's Correlation Coefficient was calculated with NIS Elements software version 4.03 (Nikon). At least ten layers in 3D plane were scanned for each sample and for all samples a cropped image of a minimum of 10 cells was used to determine a total of 5 colocalization coefficients. Median values for all layers and cells were calculated and used to plot Pearson's Correlation Coefficient. Experiments were performed at least five times with cells from a different healthy donor each time.

ImageStream analysis

Fixed and permeabilized cells were stained with 10 υg/ml of anti-CD46 cytoplasmic tails antibodies CYT1 (2F1) and CYT2 (13G10) directly labelled with DyLight488 and Fluoprobe647H, respectively (Lightning-Link Rapid kits, Innova Bioscience Ltd., Cambridge, UK) for 1 h at room temperature and washed twice. Cells were resuspended in 100 ul of FACS buffer and DAPI added prior to acquisition and analysis on ImageStream^{X} MARKII (using Inspire and Ideas Softwares, both Amnis Corporation, Seattle, WA). Quantitative analyses are based on the acquisition of 10,000 cell events, and MFIs were measured and calculated using an object mask to detect intracellular staining only.

Whole exome deep sequencing

Whole-exome sequencing was used to screen for mutations in candidate genes known to cause monogenic immune defects (list of genes in supplementary Table S2). We extracted genomic DNA from peripheral blood from two affected individuals (CD46-deficient patients CD46-2 [sibling of CD46-1] and CD46-3), performed whole-exome capture by using in-solution hybridization (Agilent All Exon Kit V5) and generated sequencing on the Illumina HiSeq 2000. Resulting reads were aligned to the reference human genome (UCSC Genome Browser hg19, GRCh37) with the Novoalign Software Package (Novocraft Technologies, Selangor, Malaysia). Duplicate reads, resulting from PCR clonality or optical duplicates, and reads mapping to multiple locations were excluded from downstream analysis. The only gene containing rare and damaging variants common to both subjects was CD46, as had been previously shown for both unrelated, Caucasian individuals. The respective mutations of patient CD46-2 were heterozygous variants c.175C>T (p.R59X) and c.G104G>A (p.C35Y), and patient CD46-3 had a homozygous variant in c.286+1G>C (RefSeq NM_172359) in exon 2. These mutations were confirmed by Sanger sequencing (not shown). Positions with normalized SIFT probabilities less than 0.05 are predicted to be deleterious, those greater than or equal to 0.05 are predicted to be tolerated. Also, columns EVS and 1KG indicate the minor allele frequencies in two large population genetics databases, and all positions observed are not rare and are thus not suggestive of causing an immune defect. The seemingly rare mutation in TARP 38299727 occurs very frequently in our in house database of normal donors and is therefore also not suggestive of being causal.

RT-PCR. Primers used to quantify *CD46* mRNA transcription patterns depicting all four isoforms in one reaction are as follows: (F) 5’-GTG GTC AAA TGT CGA TTT CCA GTA GTC

G-3’ and (R) 5’-CAA GCC ACA TTG CAA TAT TAG CTA AGC CAC A-3’ (Wang et al., 2000). *ACTB* (β-actin) control, (F) 5’-AGC ACA GAG CCT CGC CTT T-3’ and (R) 5’-CAC GCA GCT CAT TGT AGA AG-3’. PCR reactions were performed using the QIAGEN OneStep RT-PCR Kit (210212 Manchester, UK) according to the manufacturer’s protocol and with the specific annealing temperatures/times calculated for each primer pair.

Lentiviral transfection of T cells with CD46 CYT-1 or CYT-2. For CD46 cytoplasmic tails overexpression in human T cells, an appropriate Lentivirus was generated by cotransfecting HEK293T cells with packaging plasmid psPAX2 (Addgene, Cambridge, MA), envelope plasmid pMD2.G (Addgene), and pCDH-CMV-MCS-EF1-copGFP (System Biosciences, San Diego, CA) vector containing either exon 13 of CD46 (CYT1) or exon 14 of CD46 (CYT2) in presence of 0.25 M $CaCl_2$. After 48 h and 72 h incubation, media were harvested, filtered and viral particles were precipitated using PEG-it TM Virus Precipitation Solution (System Biosciences, Mountain View, CA), as per manufacturer’s protocol. Viral particles were then added in serial dilutions to $CD4^+$ T cells that had been isolated from Patient CD46-3 and cells were seeded at 1 x 10^5 cells/well into 96-well plates coated with antibodies to CD3 and CD28. Virus-infected cells were monitored by FACS analyses for GFP-positivity at x d post transfection and cytokines measured at indicated time points.

Gene arrays and array analyses. Transcriptome profiling was performed using Illumina HT12V4 microarrays (Illumina Inc., Great Chesterford, UK). RNA was isolated from 10^6 resting $CD4^+$ T cells, along with $CD4^+$ T cells treated with anti-CD3 and anti-CD46 for 2 h from CD46-deficient Patient CD46-3 and an age- and sex-matched healthy donor using a Qiagen Allprep Kit according to the manufacturer’s instructions (80004, Qiagen). Potential genomic DNA contamination was removed by Turbo DNase treatment (AM2238, Ambion/Life Technologies). Technical triplicates of gene expression analysis was conducted using 5 pg total RNA per sample which was labelled and amplified using Ovation Pico WTA System V2 and Encore BiotinIL Kits (NuGen Inc., San Carlos, CA) to generate cDNA. This cDNA was hybridized to Illumina HT12V4 Microarrays which were then washed and scanned using an Illumina iScan System and subjected to quantile normalization within the Genome Studio Suite v1.0 (Illumina).

Expression data were analyzed using Partek Genomics Suite (Partek Inc., St Louis, USA) version 6.6, Ingenuity Pathway Analysis (Qiagen) and Gene Set Enrichment Analysis, GSEA (Subramanian et al., 2005) (Broad Institute of MIT and Harvard). Differentially expressed genes were defined as those who expression was significantly different ($p<0.05$) between patient CD46-

3 and healthy donor (HD) by at lest 1.5 fold. GSEA compared RPKM values of HD to patient CD46-3 for enrichment of the Gene Ontology gene set "Regulation of Metabolic Process", using log2 ratio of classes and 10,000 permutations.

SUPPLEMENTAL REFERENCES

Cardone, J., Le Friec, G., Vantourout, P., Roberts, A., Fuchs, A., Jackson, I., Suddason, T., Lord, G., Atkinson, J.P., Cope, A., *et al.* (2010). Complement regulator CD46 temporally regulates cytokine production by conventional and unconventional T cells. Nat Immunol *11*, 862-871.

Kemper, C., Leung, M., Stephensen, C.B., Pinkert, C.A., Liszewski, M.K., Cattaneo, R., and Atkinson, J.P. (2001). Membrane cofactor protein (MCP; CD46) expression in transgenic mice. Clin Exp Immunol *124*, 180-189.

Mrkic, B., Pavlovic, J., Rülicke, T., Volpe, P., Buchholz, C.J., Hourcade, D., Atkinson, J.P., Aguzzi, A., and Cattaneo, R. (1998). Measles virus spread and pathogenesis in genetically modified mice. J Virol *72*, 7420-7427.

Subramanian, A., Tamayo, P., Mootha, V.K., Mukherjee, S., Ebert, B.L., Gillette, M.A., Paulovich, A., Pomeroy, S.L., Golub, T.R., Lander, E.S., *et al.* (2005). Gene set enrichment analysis: a knowledge-based approach for interpreting genome-wide expression profiles. Proc Natl Acad Sci U S A *102*, 15545-15550.

Wang, G., Liszewski, M.K., Chan, A.C., and Atkinson, J.P. (2000). Membrane cofactor protein (MCP; CD46): isoform-specific tyrosine phosphorylation. J Immunol *164*, 1839-1846.

Weyand, N.J., Lee, S.W., Higashi, D.L., Cawley, D., Yoshihara, P., and So, M. (2006). Monoclonal antibody detection of CD46 clustering beneath Neisseria gonorrhoeae microcolonies. Infect Immun *74*, 2428-2435.
